# DNA Aptamer-Based Staining and Fluorescence Microscopy for Rapid Detection of *Cyclospora Cayetanensis* Oocysts

**DOI:** 10.1007/s10895-023-03533-4

**Published:** 2023-12-18

**Authors:** John G. Bruno, Jeffrey Sivils, Mohan Natarajan, Sumathy Mohan

**Affiliations:** 1 Nanohmics Inc., 6201 E. Oltorf Street Suite 400, Austin, TX 78741 USA; 2 Department of Pathology and Laboratory Medicine, University of Texas Health Science Center at San Antonio, 7703 Floyd Curl Drive, San Antonio, TX 78229 USA

**Keywords:** Aptamer, *Cyclospora*, Oocyst, Food safety, Fluorescence microscopy, Rapid method, Parasite

## Abstract

There are no commercial antibodies for detection of *Cyclospora cayetanensis,* only a relatively slow polymerase chain reaction (PCR) test developed by the U.S. Food and Drug Administration (FDA). However, DNA aptamers have recently been developed by our group against known proteins and whole oocysts of *C. cayetanensis* and shown to specifically detect the oocysts when attached on their 5’ ends to red-emitting fluorophores and used as probes for fluorescence microscopy. Aptamers developed against recombinant wall protein 2 and TA4 antigen-like protein as well as whole oocysts specifically stained *C. cayetanensis* oocysts while exhibiting little, if any, staining of numerous other waterborne parasite species. Interestingly, the aptamers stained both exterior cell wall moieties and internal structures, suggesting that the aptamers penetrate the oocysts even without added detergents.

**Supplementary Information:**

The online version contains supplementary material available at 10.1007/s10895-023-03533-4.

## Introduction

Although not lethal, *Cyclospora cayetanensis* causes long-term diarrhea and other significant gastrointestinal issues in humans [1, 2]. This coccidian protozoan is typically ingested on fresh produce including various berries and leafy green vegetables after being deposited on the plants via contaminated irrigation water [3–5]. As such, there is a desire to detect *C. cayetanensis* oocysts on fresh produce surfaces from swab or rinsate samples and from large volumes (50-100 L) of pre-concentrated and filtered irrigation water [6–8].

A polymerase chain reaction (PCR) test developed by the U.S. FDA is the current standard method for detection of *C. cayetanensis* [7, 9]. Unfortunately, PCR is relatively slow compared to rapid screening methods such as fluorescence microscopy on filters or other rapid assays and cannot detect single *Cyclospora* oocysts like microscopy can. There are no commercial antibodies available for *Cyclospora* detection [2, 10] making rapid assay methods nearly impossible to this point in time. Thus, PCR has been the front line detection method. Recently, however, our group has developed highly specific DNA aptamers against known recombinant TA4 antigen-like protein and a wall protein from *C. cayetanensis* that are acknowledged in the literature [11, 12] as well as whole oocysts obtained from the U.S. Centers for Disease Control (CDC) which are described and demonstrated in this report. By using aptamers conjugated to red fluorophores (Texas Red in this work), a new complementary preliminary rapid screening method for subsequent confirmatory PCR for *Cyclospora* detection has been established.

Not only does microscopy enable single oocyst presumptive detection, especially when coupled to a red-emitting fluorophore, but it also enables preliminary identification of the rather large 7.5 to 10 μm diameter *Cyclospora* oocysts that can appear unsporulated or contain 2–4 sporocysts or sporozoites when existing outside of a human host versus closely-related, but smaller 4.2 to 5.4 μm *Cryptosporidium* oocysts containing 4 internal sporozoites [13–16] or other waterborne parasites. And because *Cyclospora* exhibits strong autofluorescence in the blue-green region of the spectrum when excited in the ultraviolet range, it can also be partially identified even without red fluorescing aptamer staining [13–17].

In the present work, we demonstrate that the aptamer probes we developed provide highly sensitive (potentially detecting one oocyst) and specific detection of *C. cayetanensis* oocysts versus the diverse morphologies of other waterborne parasites. Therefore, artificial intelligence or machine learning could be used to rapidly detect and identify *C. cayetanensis* from filter microscopy images with even greater confidence in the results to compete with or augment PCR by providing rapid initial screening.

Additionally, the screening could be coupled to processing of large volumes of rinsate or agricultural irrigation water especially after the approved EPA 1623, FDA or Bacteriological Analytical Manual (BAM) 19c filter pre-concentrating methods are employed. This publication describes the major steps already taken toward this goal to augment traditional confirmatory PCR using preliminary aptamer-based fluorescence staining intended primarily for affordable microscopic screening on filter membranes.

## Materials and Methods

### Parasites and Recombinant Protein Targets

*Cyclospora cayetanensis* oocysts were obtained from Dr. Michael Arrowood and Dr. Ana Peterson at the CDC. *Acanthamoeba* spp., *Balamuthia mandrillaris* and *Naegleria fowleri* were obtained from Dr. Ali Ibne at the CDC. *Eimaria acervulina*, *E. maxima* and *E. tenella* were obtained from Dr. Mark Jenkins at the USDA Agricultural Research Center in Beltsville, MD. *Toxoplasma gondii* was purchased from BEI Resources/ATCC, Manassas, VA. *Encephalitozoon intestinalis* and *Giardia lamblia* were purchased from Waterborne, Inc., New Orleans, LA and *Cryptosporidium parvum* oocysts were purchased from the Native Antigen Company, Kidlington, United Kingdom.

The recombinant protein targets TA4 antigen-like protein (NCBI reference sequence XP_026191783.1) and Wall Protein-2 (WP-2; GenBank OEH79762.1) were deduced from publications [11, 12] with the amino acid sequences shown in Table 1 below and custom synthesized in transfected *E. coli* and purified by Nickel affinity chromatography with 6X histidine tags by BioClone Inc. (San Diego, CA).

**Table 1:** Amino acid sequences of recombinant protein targets synthesized by BioClone

**Sporulated oocyst TA4 antigen-like** [***Cyclospora cayetanensis***] **NCBI Reference Sequence: XP_026191783.1 (294 amino acids; Collected by Jeevan Sherchand and Ynes Ortega)**
1 MRFFGDLKKR RGNGNSDSMK GEGNEAYLYS TGAAGHAGHL AGMSFCGSLG QGSHKYLYCS 61 PNRLQKMAPF SLLSLASASL LLAHGAFAED TSVGTEVDCT TAMNALRKKA GLEAFTIHTS 121 VDAYHLPVGT HLAGDKVTTD KKEEVKELCT KILGNTADTS KRVDGDKVNL VAVQEGLSAD 181 CSAAVDYWRG AFPTFTGKPQ KFTGNQYDGK QVSFLGLLNP KSGASVNCAY YNCAKESGEG 241 SFNGLICRTQ MNVNDGELFT DEQWDKIVQA FDSGAAALPT MMAIGAAFVG LFVY
**Oocyst wall protein [Cyclospora cayetanensis] (hereby called Wall Protein-2 or WP-2) GenBank: OEH78485.1 (379 amino acids; Liu S., et al. BMC Genomics 17:316, 2016):**
1 MVDGLCITPA APVLECPNGY INICKAKDRA ESPCCAKGHT AEKIARCREG MQSQDGYCTS 61 IVAHEAVTEC PAGYALINHG LQCIKQERGQ AAAACVAPDV LSAEGDSCLR TMQQGYEYIC 121 PDEYQCIAYA HTKKKYSPVC SACAKTTEMQ PLCGCPEGQI EVQGYCFEED VYGVCQRHQG 181 MPRKQAPSKR QPVKPTKKGE EAPEPSCSPV GRVSCSCEES YTLHCTSNIC TCINREIIPV 241 VPICRGELDE SGKCMAQVKT PLLYTCAEGF TCDVVNKKGR CHCVRVAIAE PSARCAAGEP 301 HKGKCMEVVR EQKIVECPQG YSETCCDNHC SCTKTHLATR EVKCASGAVS IQGECVYVSQV 361 PSPGCEVVSL AIDLVALSA

### Systematic Evolution of Ligands by EXponential (SELEX) Enrichment DNA Aptamer Development

Two different SELEX methods were used: **(1)** a method involving recombinant parasite proteins displayed on magnetic microbeads (MBs) and **(2)** whole oocyst SELEX. For the first method, 100 µg of each of the recombinant proteins (TA4-like antigen and WP-2) were dissolved in 1 ml of sterile phosphate buffered saline (PBS, pH 7.2) and separately immobilized on 100 µl of stock 2.8 micron Dynal (M280) tosyl- coated-magnetic beads (MBs, Invitrogen Corp., Carlsbad, CA) for 2 h at 37^o^C. Recombinant protein- conjugated MBs were then collected using a Dynal MPC-S magnetic rack and washed three times in 1 ml of sterile PBS. Protein-MBs were next blocked for 2 h at 37^o^C in sterile PBS plus 2% ethanolamine and washed three times as before in 1 ml aliquots of sterile PBS. DNA aptamers were developed against each of the protein-conjugated MBs through ten rounds of selection and PCR amplification as previously described in the literature [18]. In the second whole oocyst method, aptamers were selected and developed against approximately 10^5^* C. cayetanensis* oocysts (designated whole cell or WC) per round of aptamer selection in which case the oocysts were centrifuged at 13,000 x g for 5 min and the unbound oligonucleotides were siphoned off and discarded. Then 95˚C heat-eluted aptamers in sterile deionized water were siphoned from the supernate leaving the oocyst pellet undisturbed as previously described in the literature [19, 20]. The following 72 base single-stranded SELEX aptamer template with 36 randomized (designated N36) bases in the central region, forward and reverse primers were synthesized by Integrated DNA Technologies (IDT; Coralville, Iowa) and used for SELEX:

#### SELEX Template:

5’-ATCCGTCACACCTGCTCT-N36-TGGTGTTGGCTCCCGTAT-3’

#### Forward Primer:

3’-ACCACAACCGAGGGCATA-5’

#### Reverse Primer:

5’-ATCCGTCACACCTGCTCT-3’

The presence of 72 bp PCR aptamer amplicon products were verified after each round of selection by ethidium bromide-stained 2% agarose gel electrophoresis against standard DNA ladders. Aptamers from the tenth round of selection and amplification pool were sent to Base Pair, Inc., Pearland, TX for Illumina-based next generation sequencing (NGS). The top 10 most frequent consensus aptamer DNA sequences were screened and ranked by absorbance at 405 nm by an ELISA-like enzyme-linked aptamer sorbent microplate assay (ELASA) to determine which candidate aptamer sequences against TA4-like antigen, WP-2 and whole cell (WC) *Cyclospora* oocysts exhibited the highest affinity to move forward in assay development. Due to the potential commercial value of these selected aptamers, their DNA sequences cannot be divulged.

### ELASA Method

To evaluate relative affinity rankings for each of the candidate aptamers, an enzyme-linked aptamer sorbent assay (ELASA) was conducted by first immobilizing serial two-fold dilutions of TA4 Antigen-like and WP-2 recombinant proteins beginning at 1,000 ng per well in 100 µl of 0.1 M NaHCO_3_ (pH 8.5) overnight at 4^o^C in covered flat-bottom polystyrene 96-well plates (Greiner Bio-One GmbH, Frickenhausen, Germany). The plates were decanted and washed 3 times in 200 µl of PBS. Wells were then blocked with 150 µl of 10% ethanolamine in 0.1 M NaHCO_3_ for 1 h at 37^o^C followed by 3 more washes with 200 µl of PBS as before. The 4 most common aptamer DNA sequences from NGS for both TA4 Antigen-like and WP-2 recombinant proteins were synthesized with 5’-biotin linkers by IDT and rehydrated in 100 µl of PBS for 1 h with gentle mixing on an orbital rotary mixer and applied to their corresponding microplate wells at 1 nanomole per well for 1 h at room temperature with gentle mixing. The plates were decanted and washed 3 times in 200 µl of PBS for at least 5 min per wash with gentle mixing. One hundred µl of a 1:5,000 dilution of streptavidin-peroxidase from a 1 mg/ml stock solution (Thermo Fisher Scientific, Product No. 21,126) in PBS was added per well for 30 min at room temperature with gentle mixing. The plates were decanted and washed 3 times with 200 µl of PBS per well as before. One hundred µl of One-Component® ABTS substrate (Kirkegaard Perry Laboratories, Inc., Gaithersburg, MD) which had been equilibrated to room temperature was added to each well and incubated for 15 min at room temperature. Reactions were halted by addition of 100 µl of 1% SDS as the strongest reactions approached an absorbance of 2.0 at 405 nm using a Thermo Electron MultiSkan^™^ microplate reader (Thermo Fisher Scientific; Waltham, MA).

### Aptamer-Fluorophore Conjugate Staining Protocol and Confocal Microscopy

Purified *Cyclospora* oocysts obtained from the CDC and other parasites were pelleted by spinning 1 ml (~ 10^6^ oocysts) of each sample on a microfuge for 2 minutes at 13,000 x g. Each of the 5’-biotinylated candidate DNA aptamers from IDT was completely dissolved in 1 ml of sterile PBS (pH 7.2–7.4) at ~ 1.5 mg/ml. Next, 100 µl of each 5’-biotinylated aptamer was added to the pellet of oocysts and gently mixed up and down using a pipette tip and orbital shaker for 30 min at room temperature to allow the aptamers to bind. The oocysts were pelleted by centrifugation on the microfuge again and the supernate was carefully siphoned off and discarded. One ml of sterile PBS was added and the oocyst pellet was resuspended. The oocysts were again pelleted by centrifugation and resuspended in 1 ml of sterile PBS containing a 1:100 dilution of streptavidin-Texas Red conjugate from Millipore Sigma (Cat. No. 189738) which was allowed to bind the 5’-biotin on any bound aptamers on or inside of the oocysts for 30 min. Oocysts were then washed twice as before in 1 ml of PBS, added to glass slides with coverslips and examined under a confocal fluorescence microscope.

## Results

**Fig. 1:**
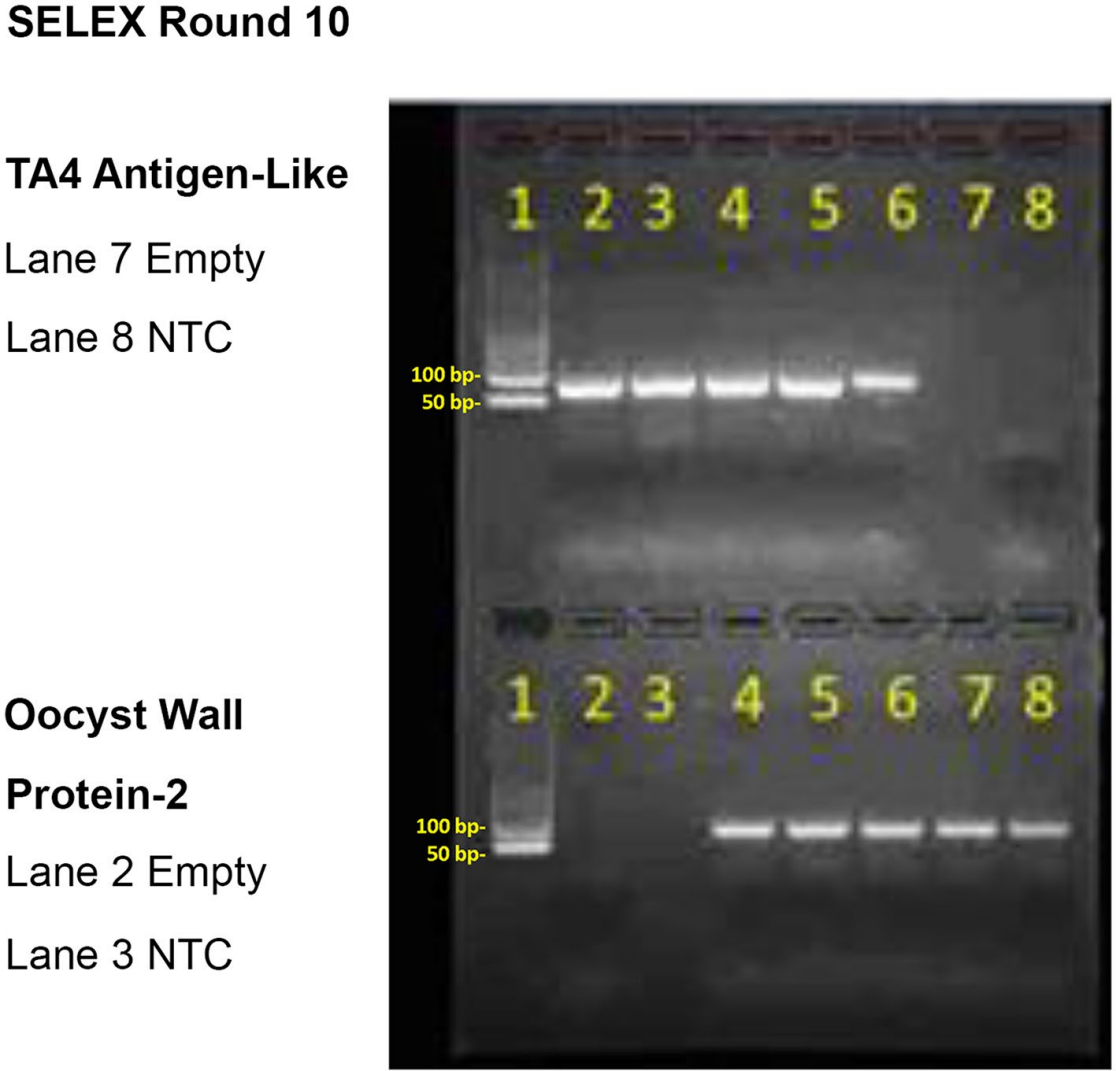
Ethidium bromide (EtBr)- stained 2% agarose electrophoretic gel showing PCR amplicons of several aliquots of round 10 SELEX DNA aptamers to prove successful aptamer development. Aptamer bands at 72 bp fall between the 50 and 100 bp standard bands in the DNA ladders in lane 1. NTC; no template (negative) control and empty lanes with no bands are identified by the adjacent key

Figure 1 illustrates the very clean sharp band appearance of PCR amplicons for 5 separate tubes of the TA4 antigen-like aptamers (top gel half) and Wall Protein (WP)-2 aptamers following round 10 of MB-SELEX. Similar sharp PCR amplicon bands were obtained for the whole cell (WC) *C. cayetanensis* oocyst aptamers following 10 rounds of whole cell SELEX (not shown).

**Fig. 2:**
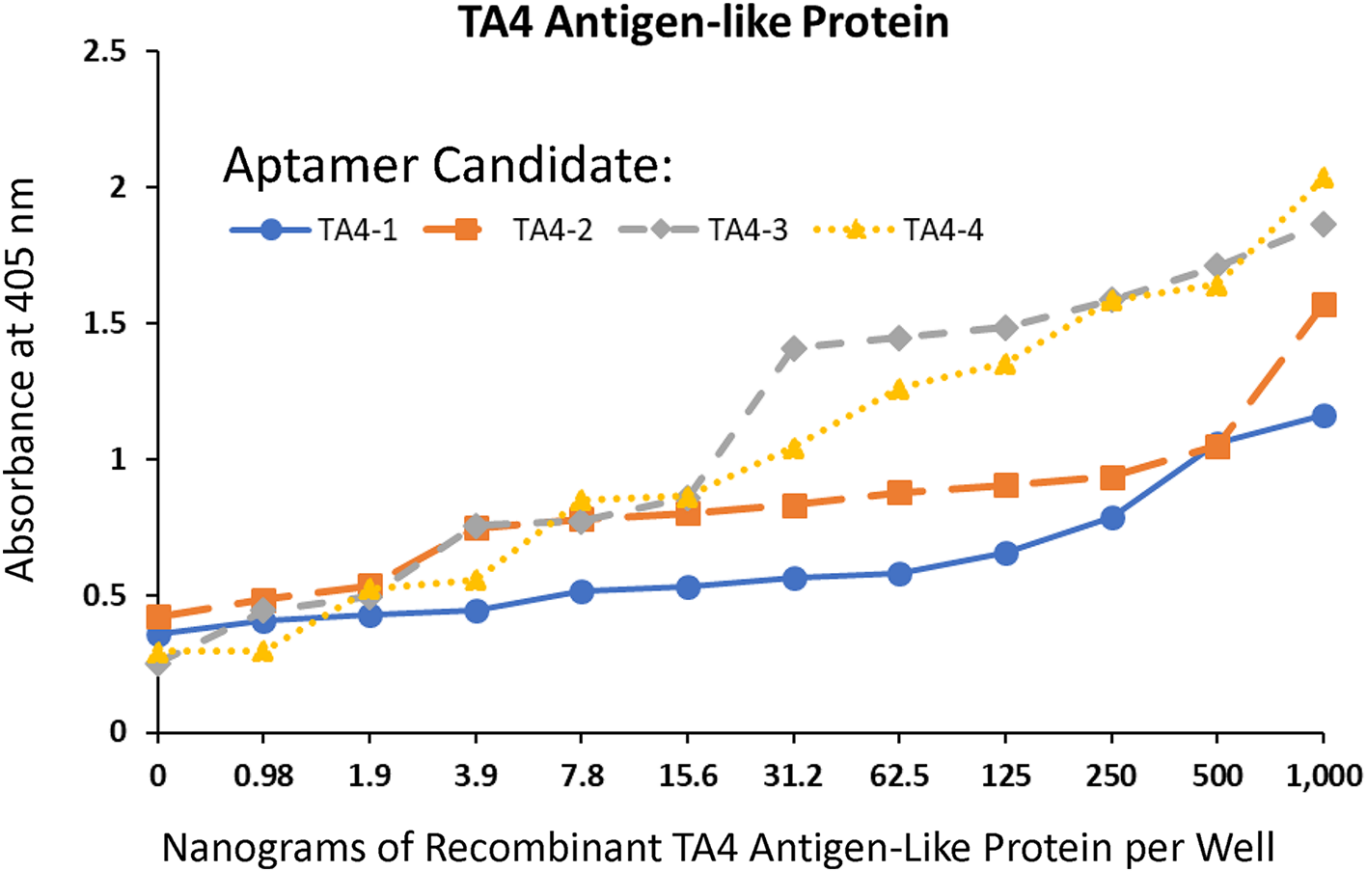
ELISA-like aptamer (ELASA) titrations with the top 4 most frequent aptamers against TA4 antigen-like protein from NGS

**Fig. 3:**
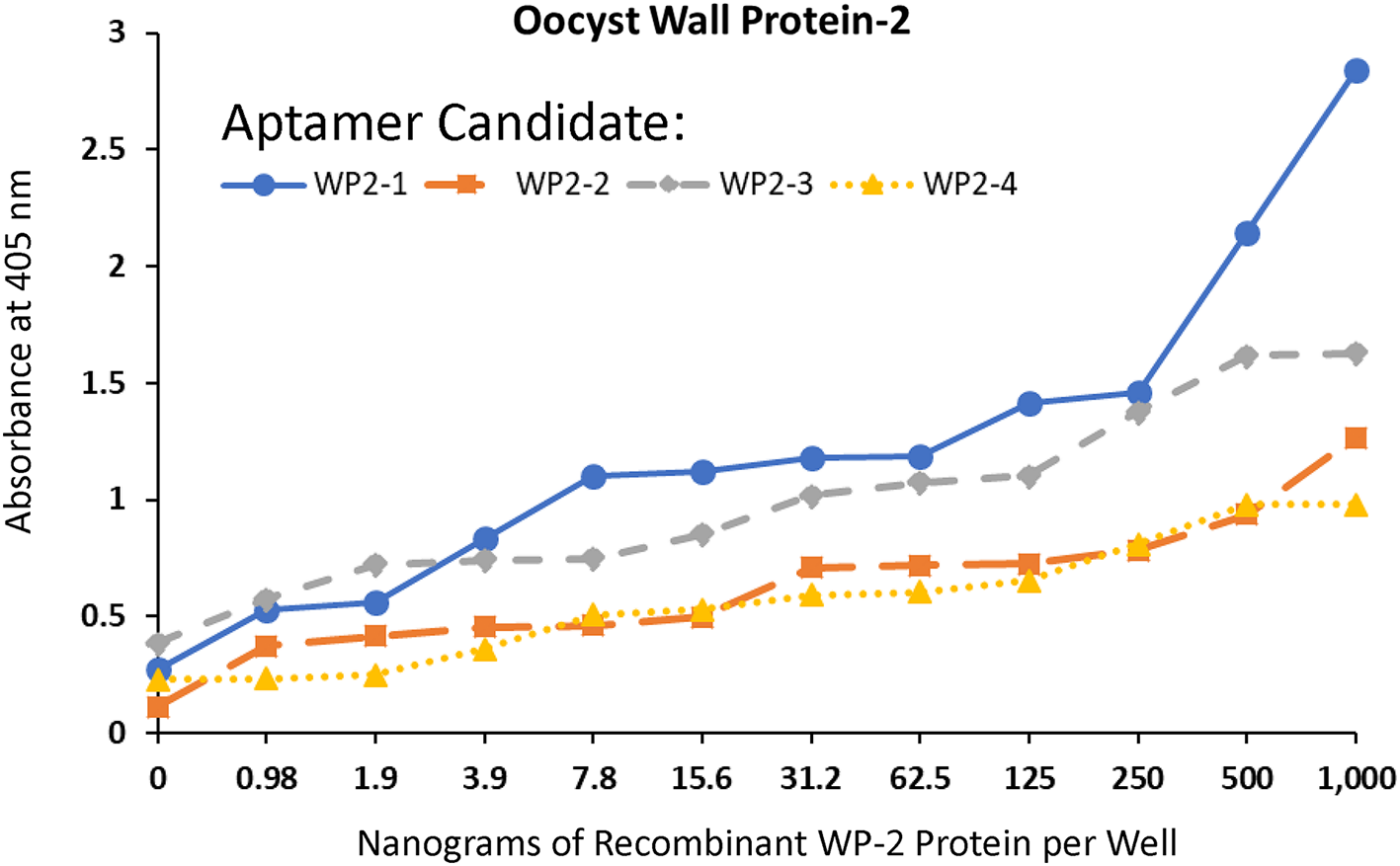
ELASA titrations with the top four most frequent aptamers against Wall Protein (WP)-2 from NGS

The ELASA titration results against two-fold serial dilutions of each recombinant *Cyclospora* protein exhibited some degree of variability on binding affinities, however, all of the candidate TA4 antigen-like and WP2 aptamers yielded low ng limits of detection as noted in Figs. 2 and 3.

**Fig. 4:**
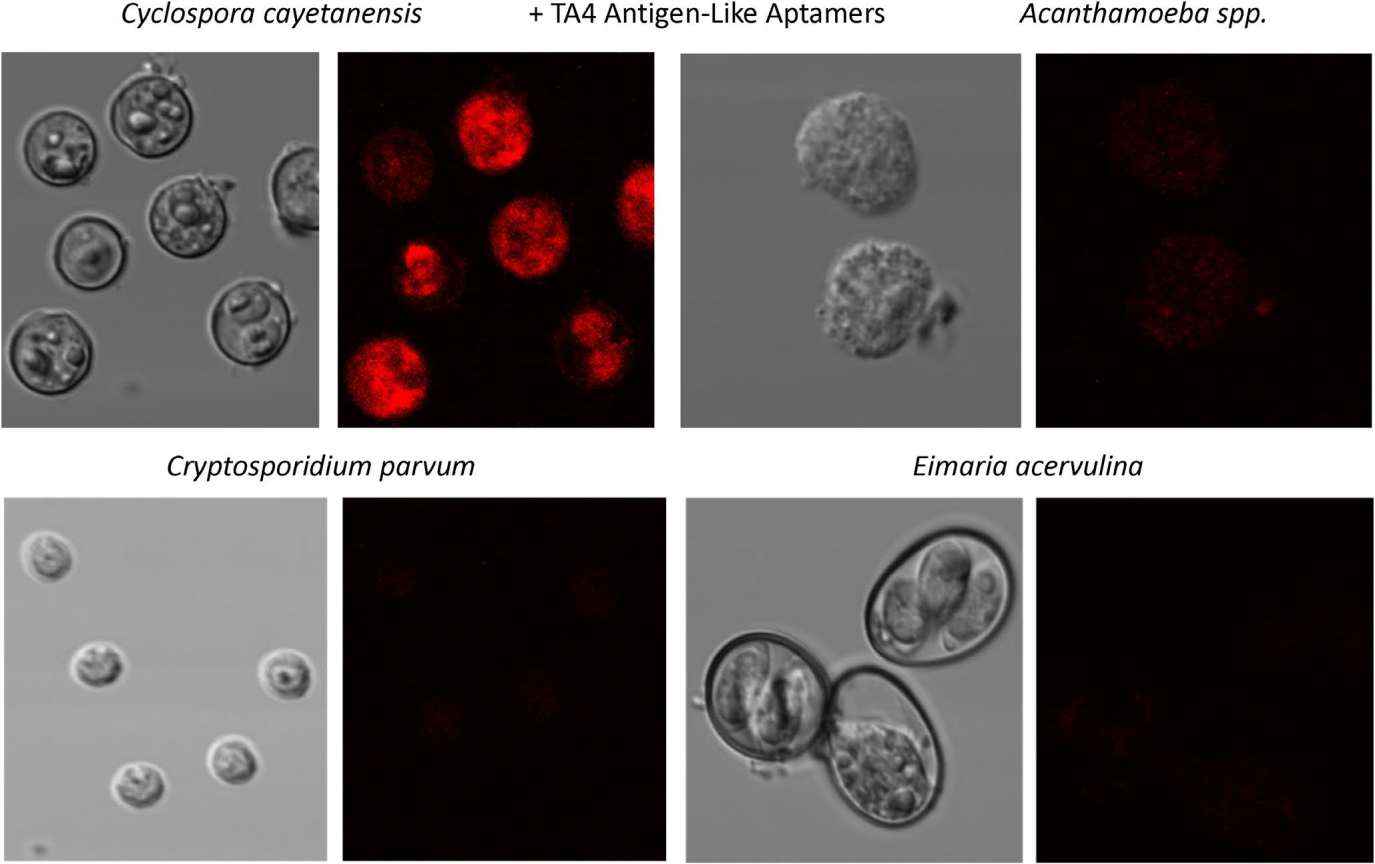
Confocal fluorescence microscopy summary results showing clear detection of *Cyclospora cayetanensis* using the most common TA4 Antigen-like aptamer sequence versus 3 other very common waterborne parasites. The Supplemental file expands greatly on these summary results by adding numerous photomicrographs of many other waterborne parasites stained with the TA4-like, WP-2 and whole cell oocyst (WC; designated S3 and S16) aptamers that strongly support the high degree of aptamer specificity for binding to only *C. cayetanensis* oocysts

Figure 4 illustrates the high degree of specific staining for *C. cayetanensis* oocysts by confocal fluorescence microscopy with a variety of the aptamer candidates developed against TA4 Antigen-like protein across 4 different waterborne parasite species with strong red staining from the Texas Red-streptavidin-biotin-aptamer complexes on *C. cayetanensis* oocysts and virtually no staining of *Acanthamoeba*, *Crytosporidium* or *Eimaria*.

**Fig. 5:**
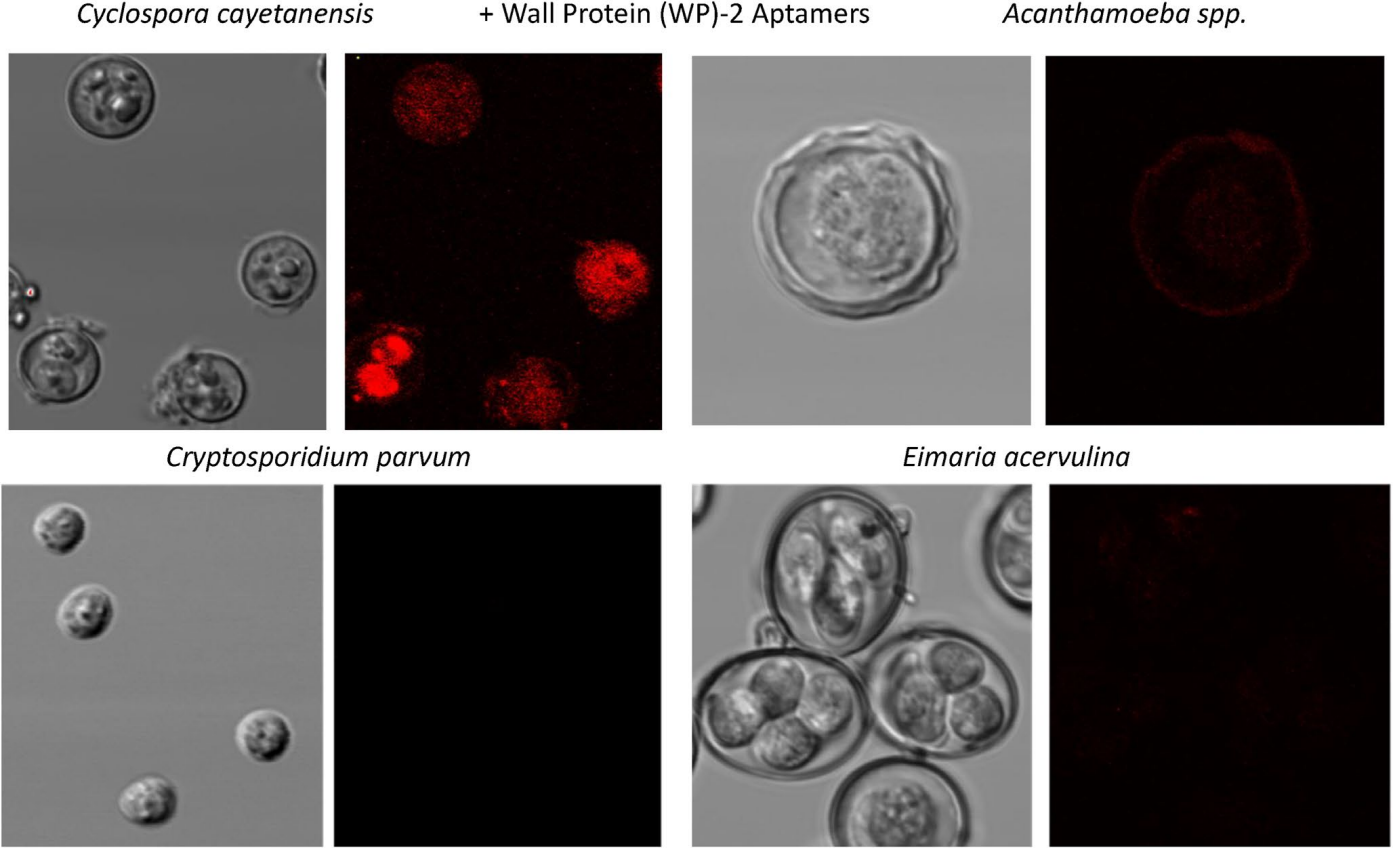
Confocal fluorescence microscopy summary results showing clear detection of *Cyclospora cayetanensis* using the most common Wall Protein (WP)-2 aptamer sequence versus 3 other very common waterborne parasites

**Fig. 6:**
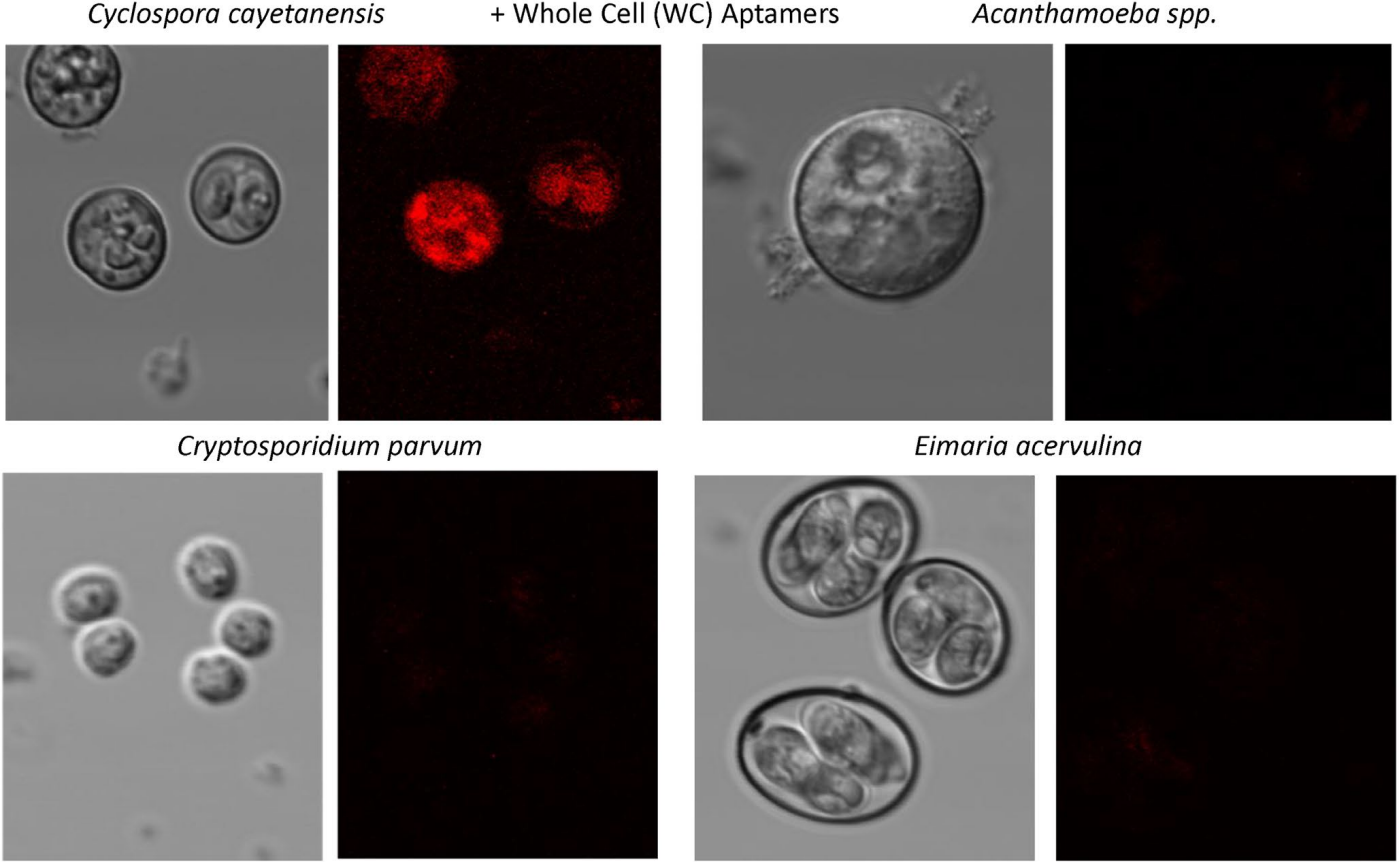
Confocal fluorescence microscopy summary results showing clear detection of *Cyclospora cayetanensis* using the most common whole oocyst or whole cell (WC) aptamer sequence versus 3 other very common waterborne parasites

Figures 5 and 6 are similar to Fig. 4 in that they illustrate the high degree of specificity of the top aptamers for detection of WP-2 and whole oocysts (WC; S3 and S16) respectively. These Figs. (5 and 6) again illustrate the very low level of fluorescence staining or no staining of *Acanthamoeba*, *Crytosporidium* or *Eimaria* with the WP-2 and WC aptamers, thus attesting to the high degree of specificity of these *C. cayetanensis* aptamer sequences.

We could not possibly present all of the specificity data in one figure or even a few figures within the body of this publication. Therefore, the supplemental file presents all of the expansive, but salient, results from our studies in one file to bolster our claim of a highly specific family of *C. cayetanensis* oocyst aptamers including all of the top 4 TA4 antigen-like and WP-2 aptamers as well as WC S3 and S16 from the whole cell oocyst SELEX aptamer development efforts. It warrants mention that while some of the waterborne pathogen parasite samples appear to show cellular damage, which may have occurred during shipping, there are enough intact cells or large cell fragments to allow us to confidently conclude that the top 10 aptamers described herein are highly specific probes for Cyclospora cayetanensis oocysts.

## Discussion

All aptamers DNA sequences developed against recombinant *Cyclospora* proteins or whole oocysts during this project worked well as probes for *C. cayetanensis* with apparently high affinity as judged by the limits of ELASA detection in the low ng range (Figs. 2 and 3) and very low cross-reactivity with numerous other waterborne parasites as seen by the lack of fluorescence staining of non-*Cyclospora* species in Figs. 4, 5 and 6 and the supplemental file. One rather curious observation about aptamer staining of the *Cyclospora* oocysts with their cognate aptamers is that it is sometimes both external surface and internal staining which is generally not seen with larger antibodies. Perhaps, the smaller size of aptamers (about 20% the size of IgG) allows them to penetrate the oocyst wall even without the addition of detergents.

The major goal of this project was to develop probes for the rapid detection of *Cyclospora* from produce rinsates or irrigation water, because no commercial antibodies exist [2, 10] and definitive PCR assays are much slower which impedes the ability to curtail or stop outbreaks. While one can use the nascent aptamers we have developed with a simple fluorescence microscope and tedious manual searching of slides, we are also developing a semi-automated fluorescence microscope to aid in the rapid screening of water filters stained with our aptamer-red fluorophore conjugates, because microscopy has a theoretical LOD of one oocyst. We intend to piggyback off of established BAM 19c or EPA 1623 methods for pre-concentration of large volumes (up to 50 or 100 L) of rinsate or irrigation water and then refilter the pre-concentrated samples onto smaller 47 mm diameter filters for microscopic analysis. The final semi-automated autofocusing microscopic instrumentation and results of its applications to rapid screening of water filters for the presence of one or more *C. cayetanensis* oocysts will be the subject of a future publication.

## Electronic Supplementary Material

Below is the link to the electronic supplementary material.

Supplementary Material 1

## Data Availability

All data generated or analyzed during this study are included in this published article and supplemental file.
