# Supplementary material for: DNA Aptamer-Based Staining and Fluorescence Microscopy for Rapid Detection of Cyclospora Cayetanensis Oocysts
Source: J Fluoresc. 2023 Dec 18;35(1):475–82. doi: 10.1007/s10895-023-03533-4 (PMC11807056; doi:10.1007/s10895-023-03533-4)
Supplement: Supplementary file 1 — Supplementary Material 1 [file 10895_2023_3533_MOESM1_ESM.pdf]

Supplemental File Notes:

All parasite species were incubated with aptamers developed against recombinant wall protein (WP-2) or TA4 antigen-like protein as well as whole *Cyclospora* cell (WC; whole oocyst) aptamers designated S3 and S16.

Conclusions:

1. The parasites and oocysts were in a wide range of sizes. To maintain uniformity the magnification was fixed for all samples at 400X total magnification.
2. The density of the oocytes in some of the samples was low. In some fields only one or two oocytes were visible.
3. Some *Cyclospora* aptamers showed very weak positive against non-cognate species.
4. Some *Cyclospora* aptamers showed very weak signals for some aptamers and totally negative for some other aptamers.
5. *Cyclospora cayetanensis* with WP-2-1 aptamer was used uniformly as an assay positive control for each set or family of species samples.
6. Parasites incubated with PBS buffer with no aptamer were used as negative assay control for all samples and never emitted red fluorescence.
7. Optimization of the assays:
    (a) Each oocyte was incubated with different incubation time (10, 30, 60 min) to rule out the possibility of insufficient incubation time being the reason for negative results (i.e., to eliminate false negatives).
    (b) The oocytes were incubated with decreasing concentrations of aptamer to rule out the possibilities of non-specific binding of biotin-labelled aptamer (100, 50, and 30 µl of 1 ml stock aptamers at ~ 1.5 mg/ml) as the cause of positive results (i.e., to eliminate false positives).

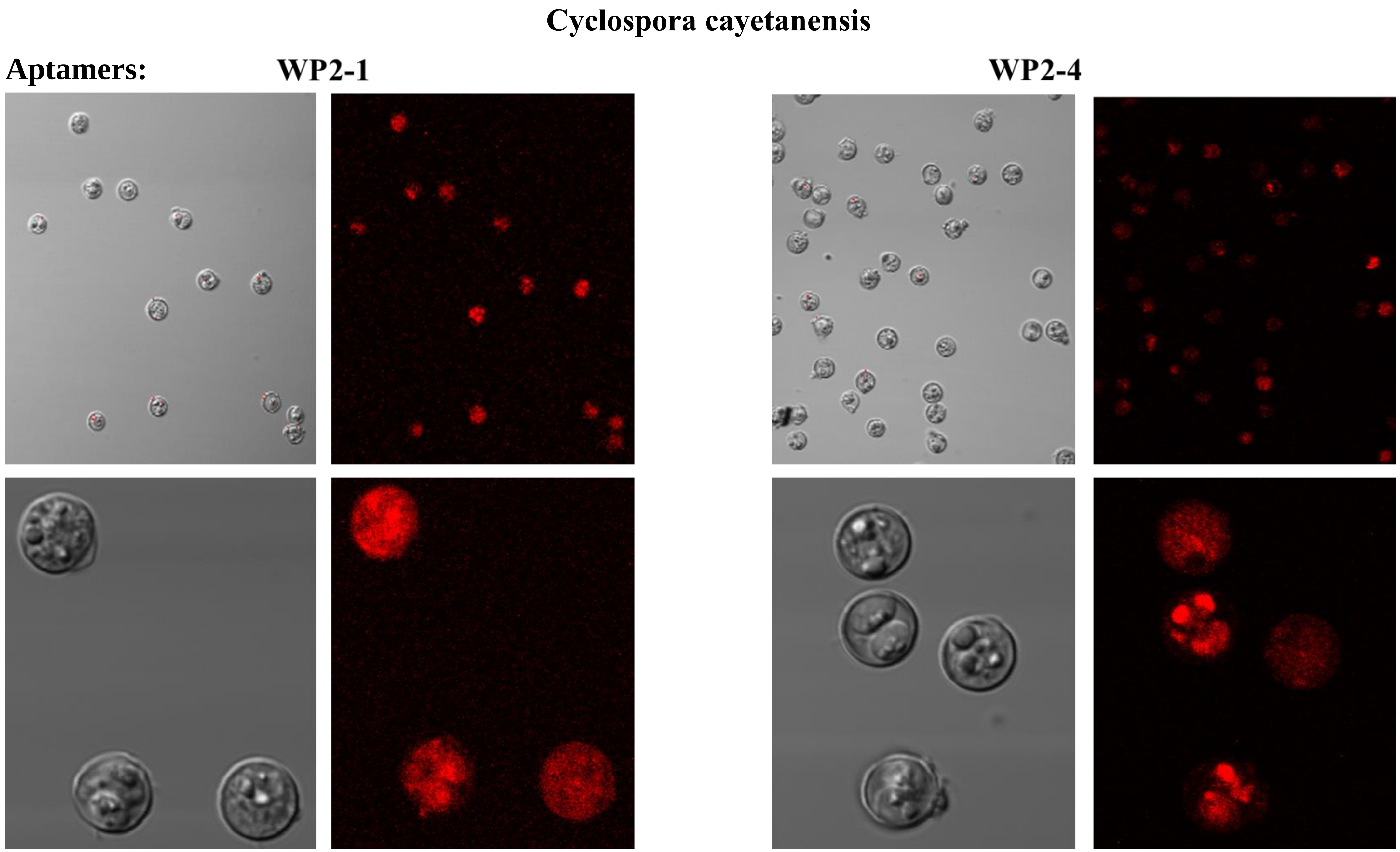
Cyclospora cayetanensis
Aptamers:
WP2-1
WP2-4

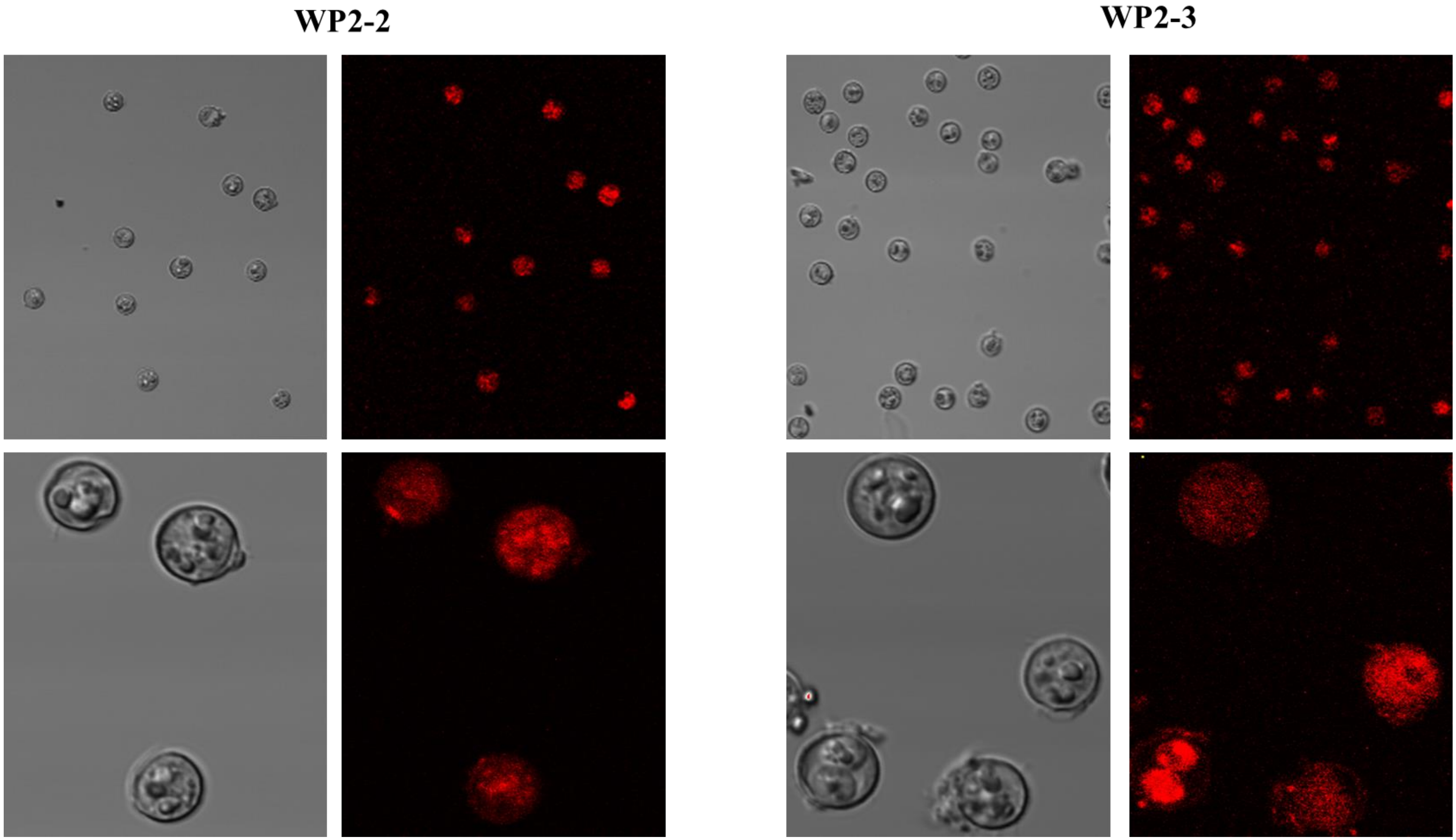
WP2-2
WP2-3

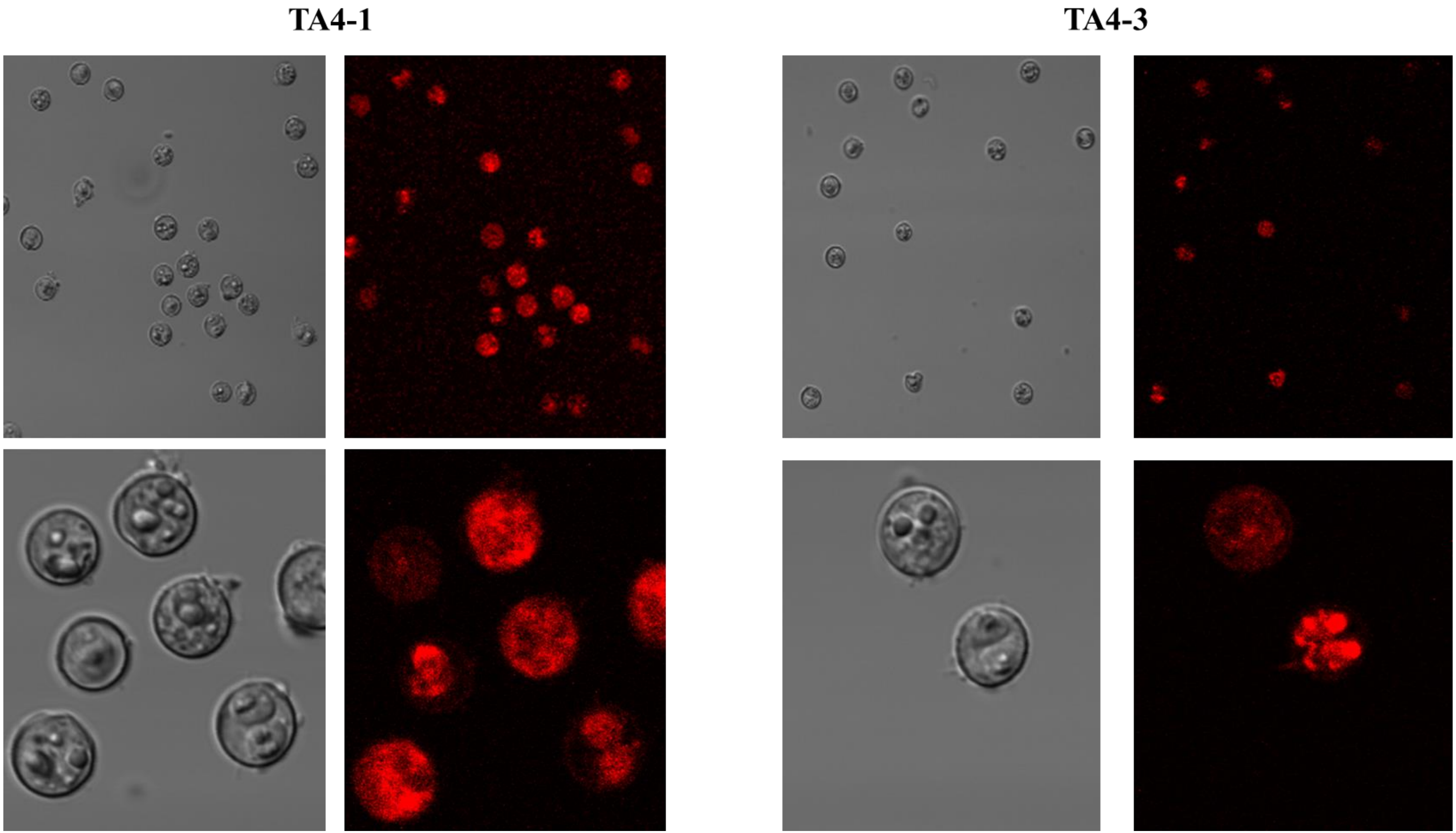
TA4-1
TA4-3

TA4-2 TA4-4

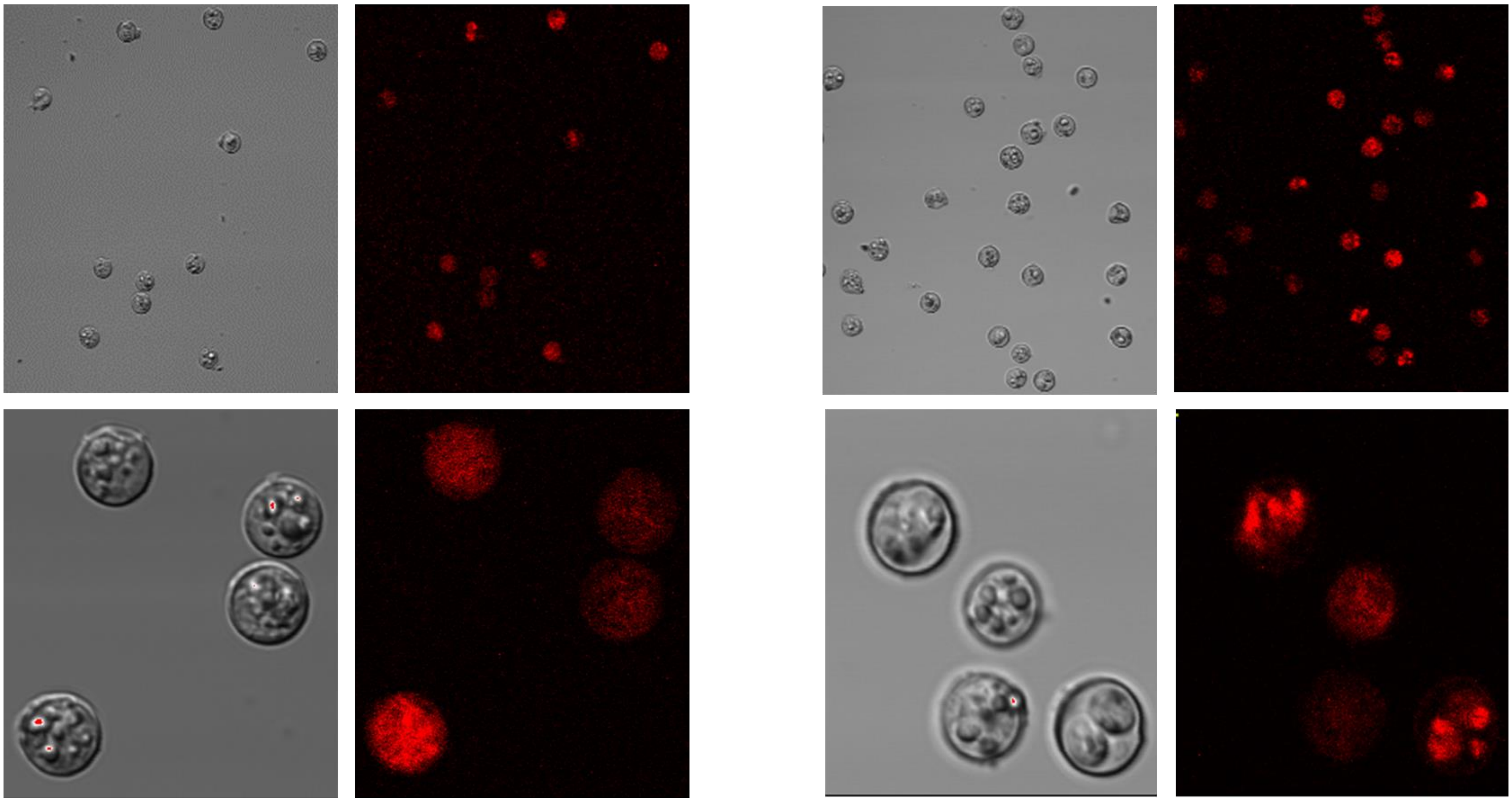

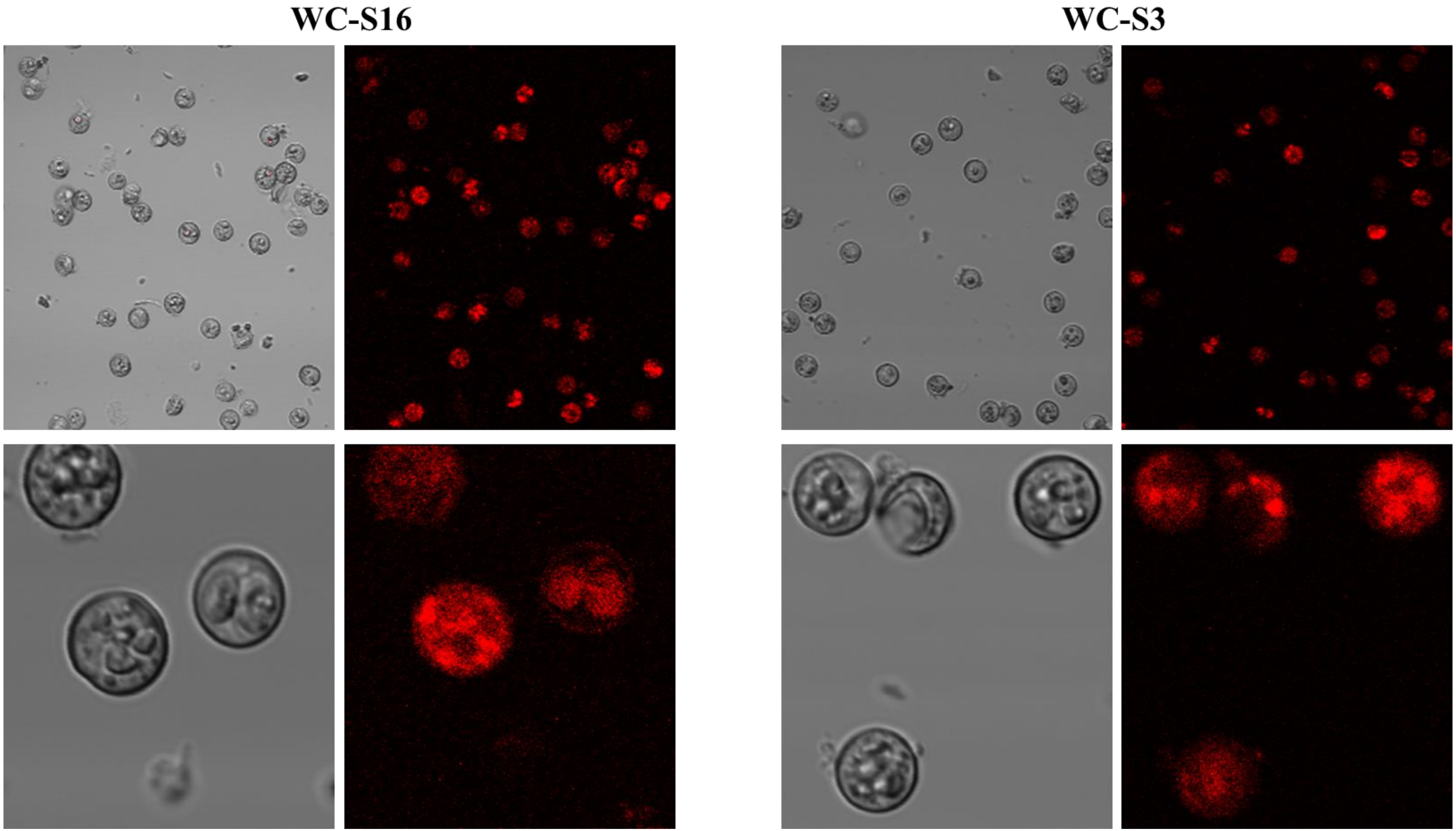
WC-S16
WC-S3

Conclusion: Cyclospora cayatenensis showed strong positive fluorescence signals with all ten different aptamers.

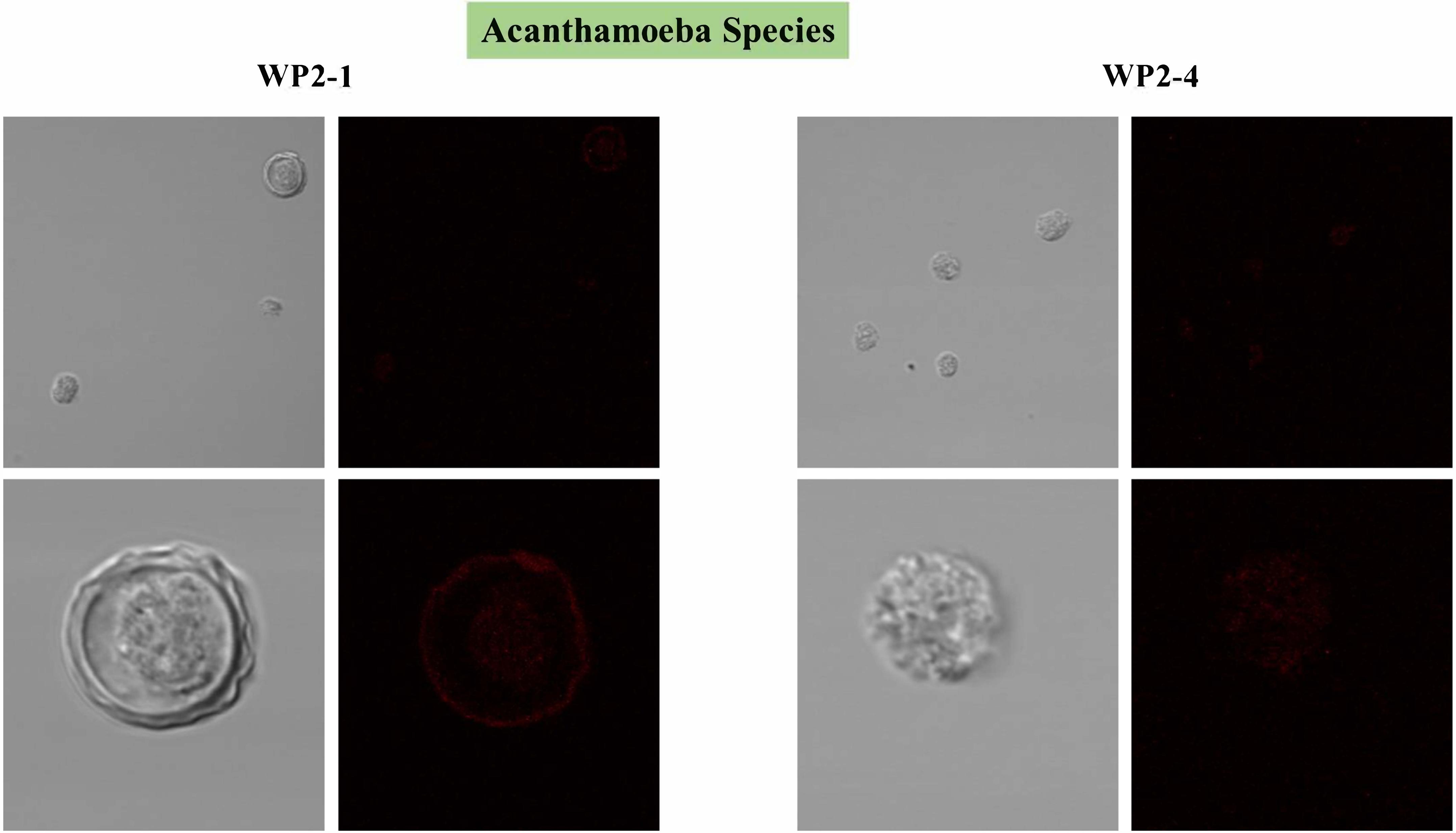
Acanthamoeba Species
WP2-1
WP2-4

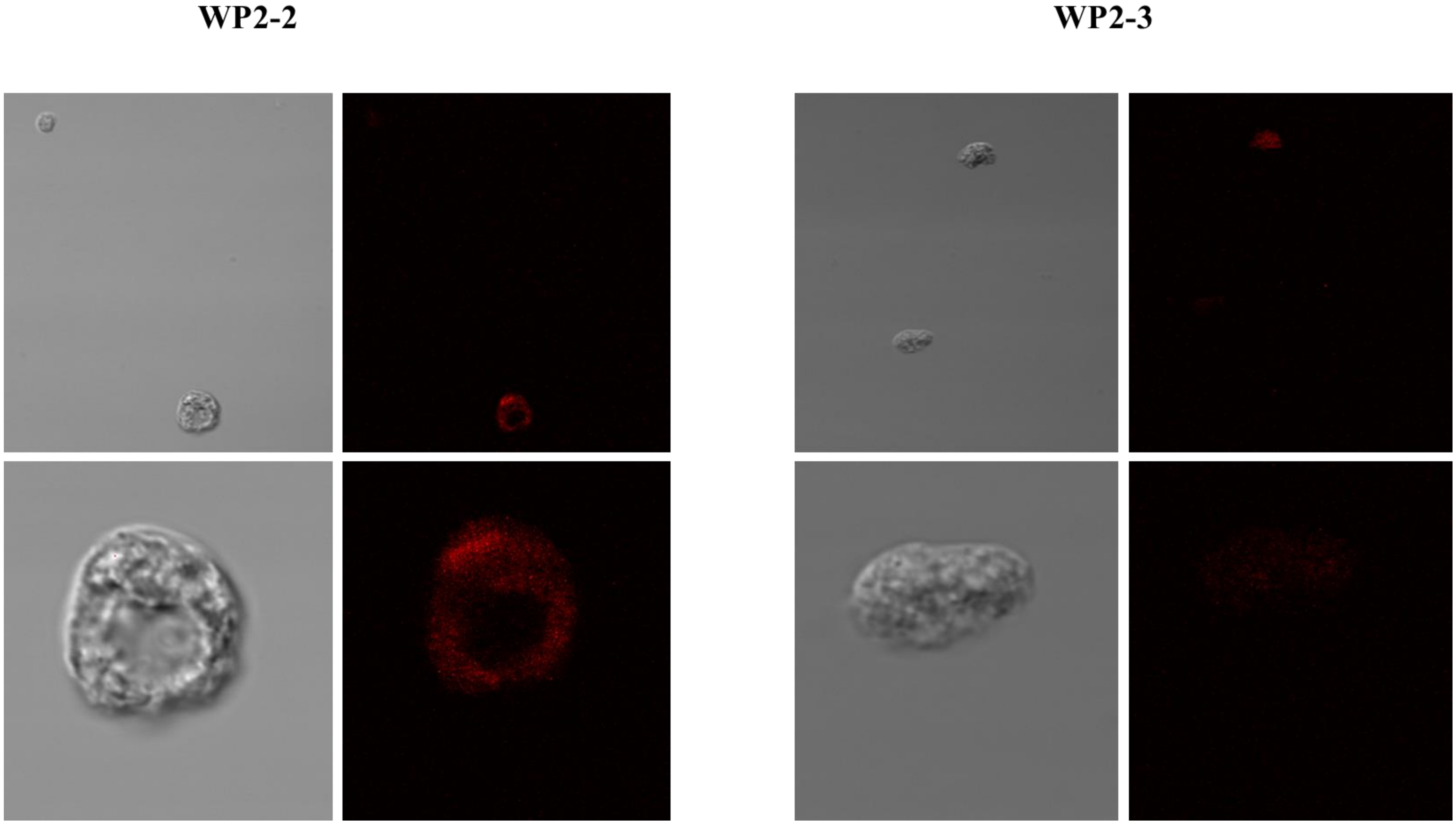
WP2-2
WP2-3

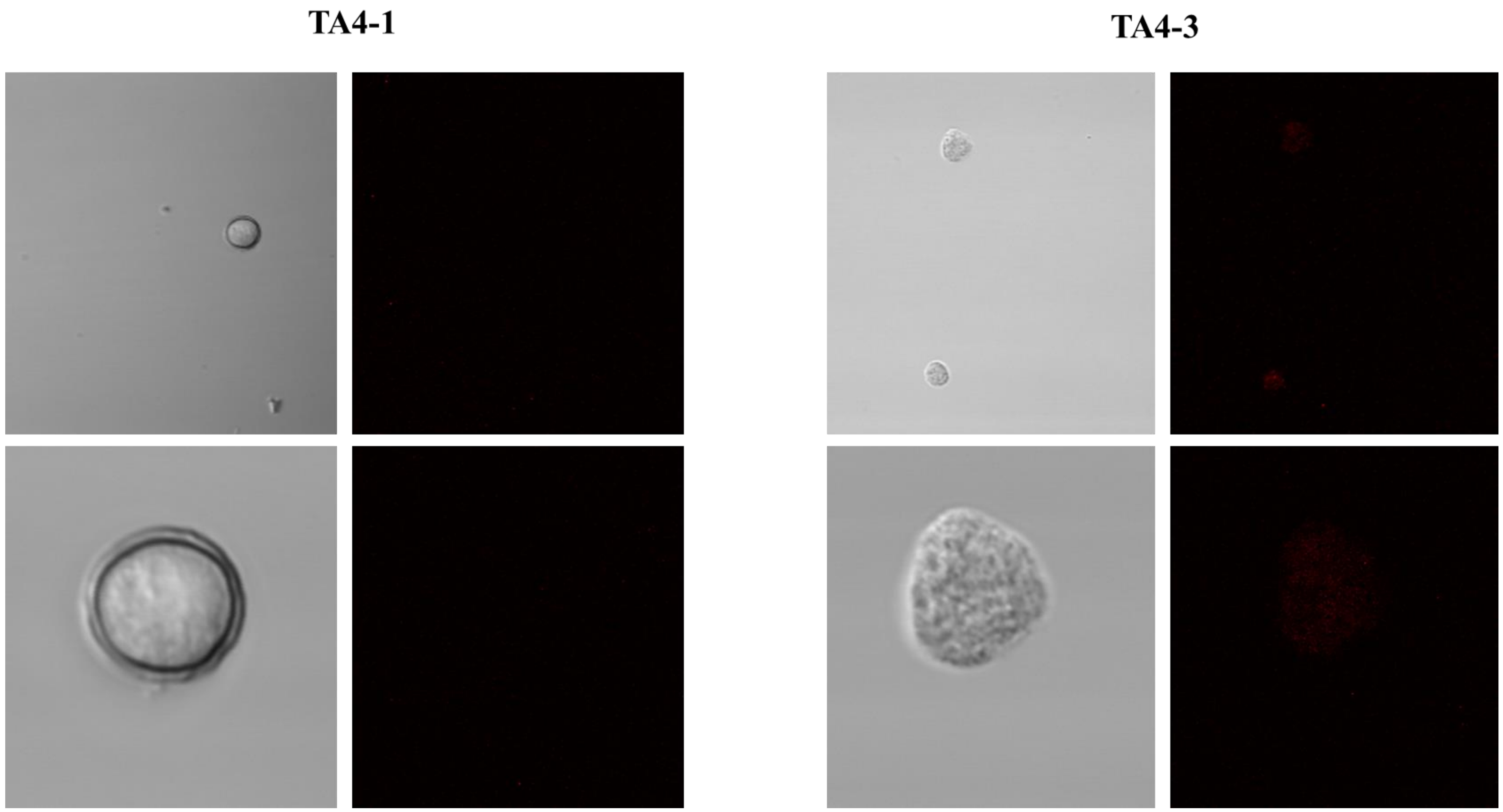
TA4-1
TA4-3

TA4-2 TA4-4

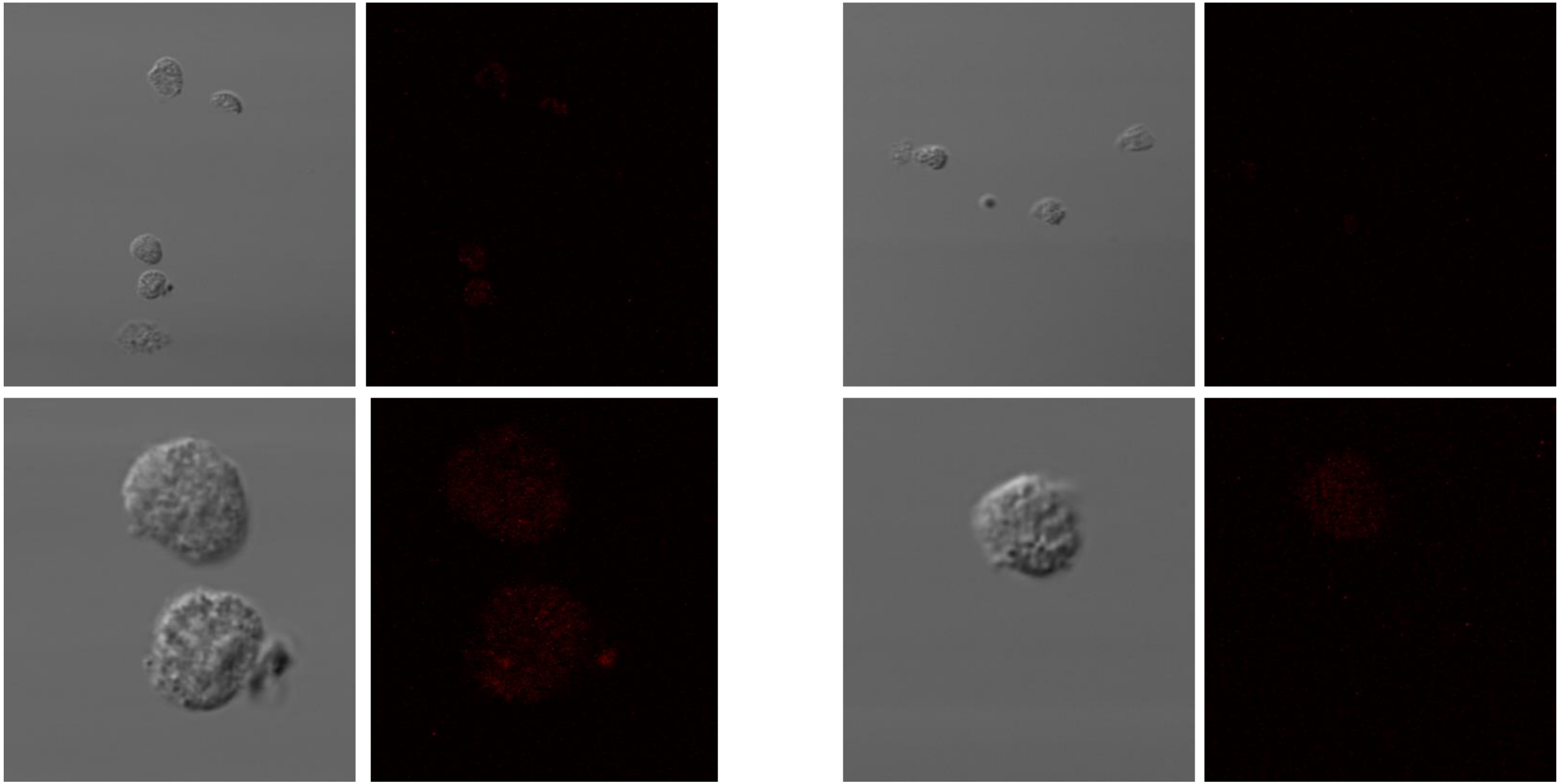

Whole Cyclo S16

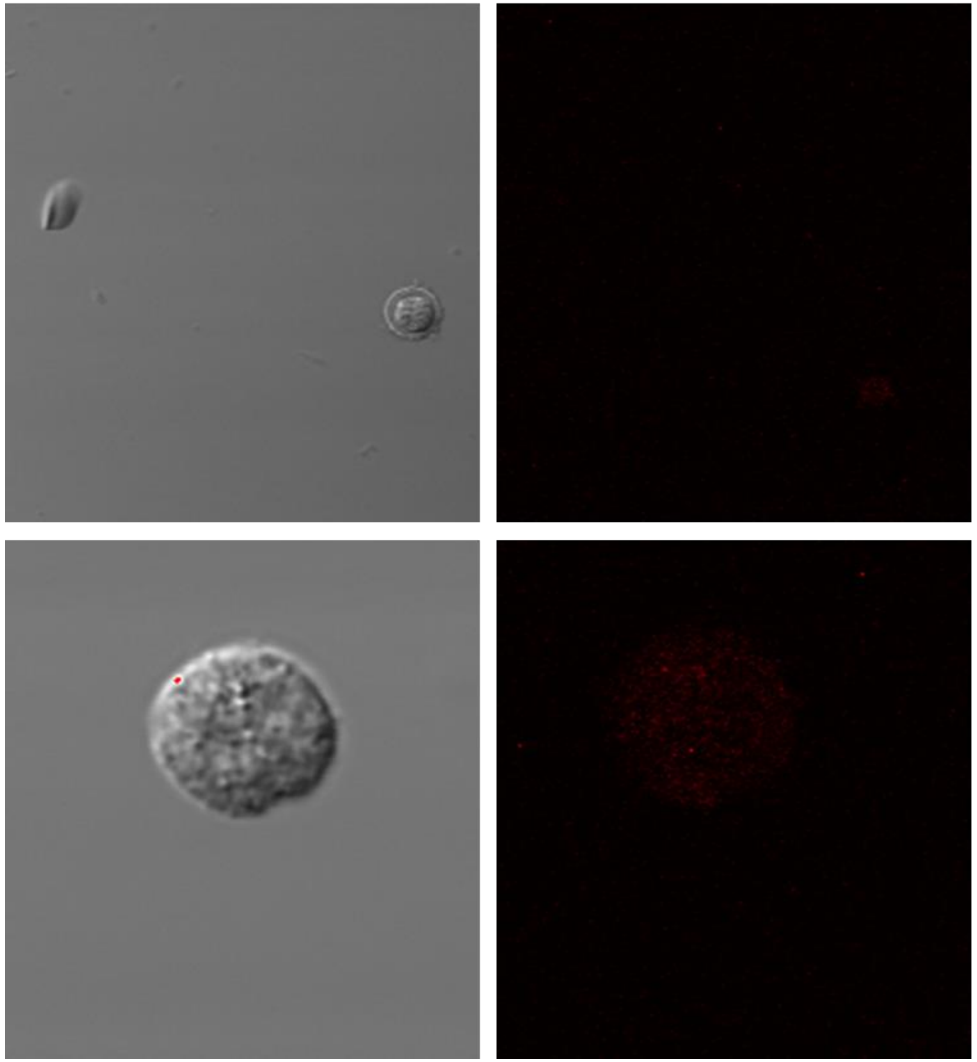

Whole Cyclo S3

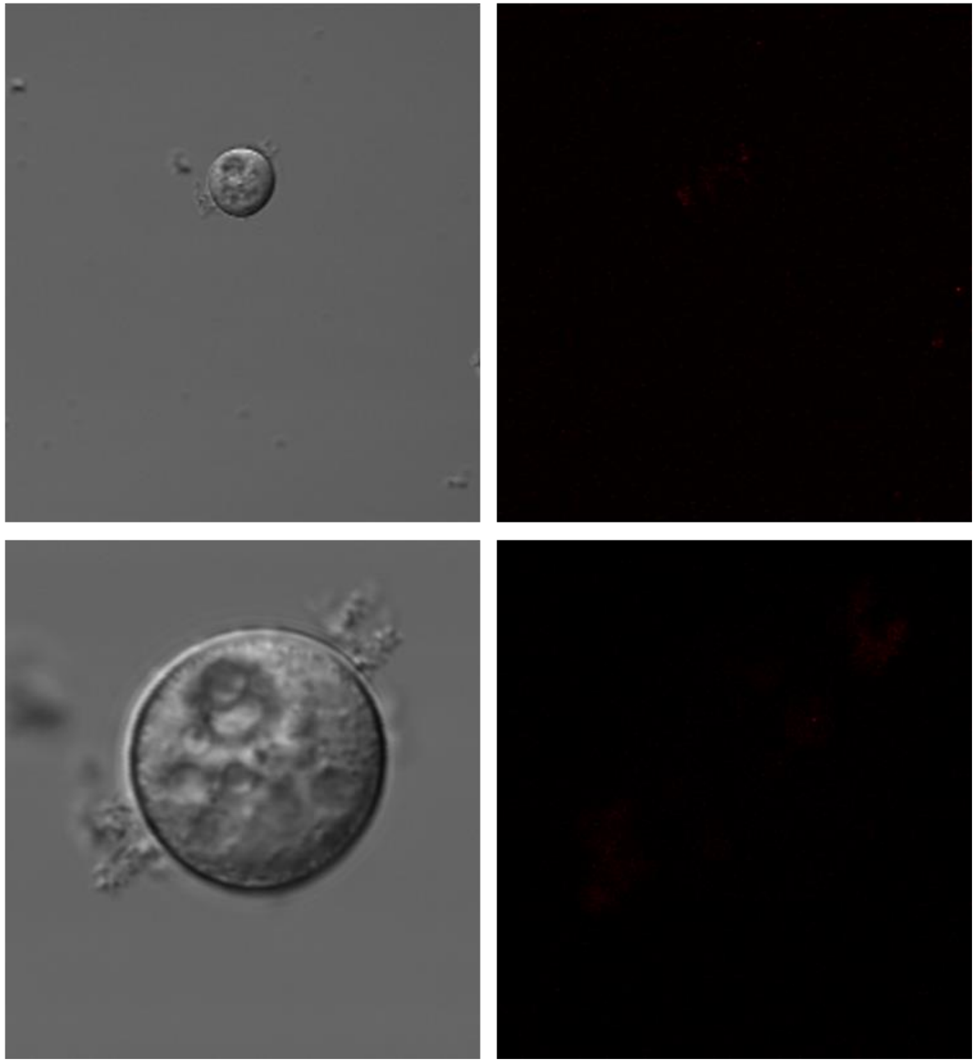

Assay Controls

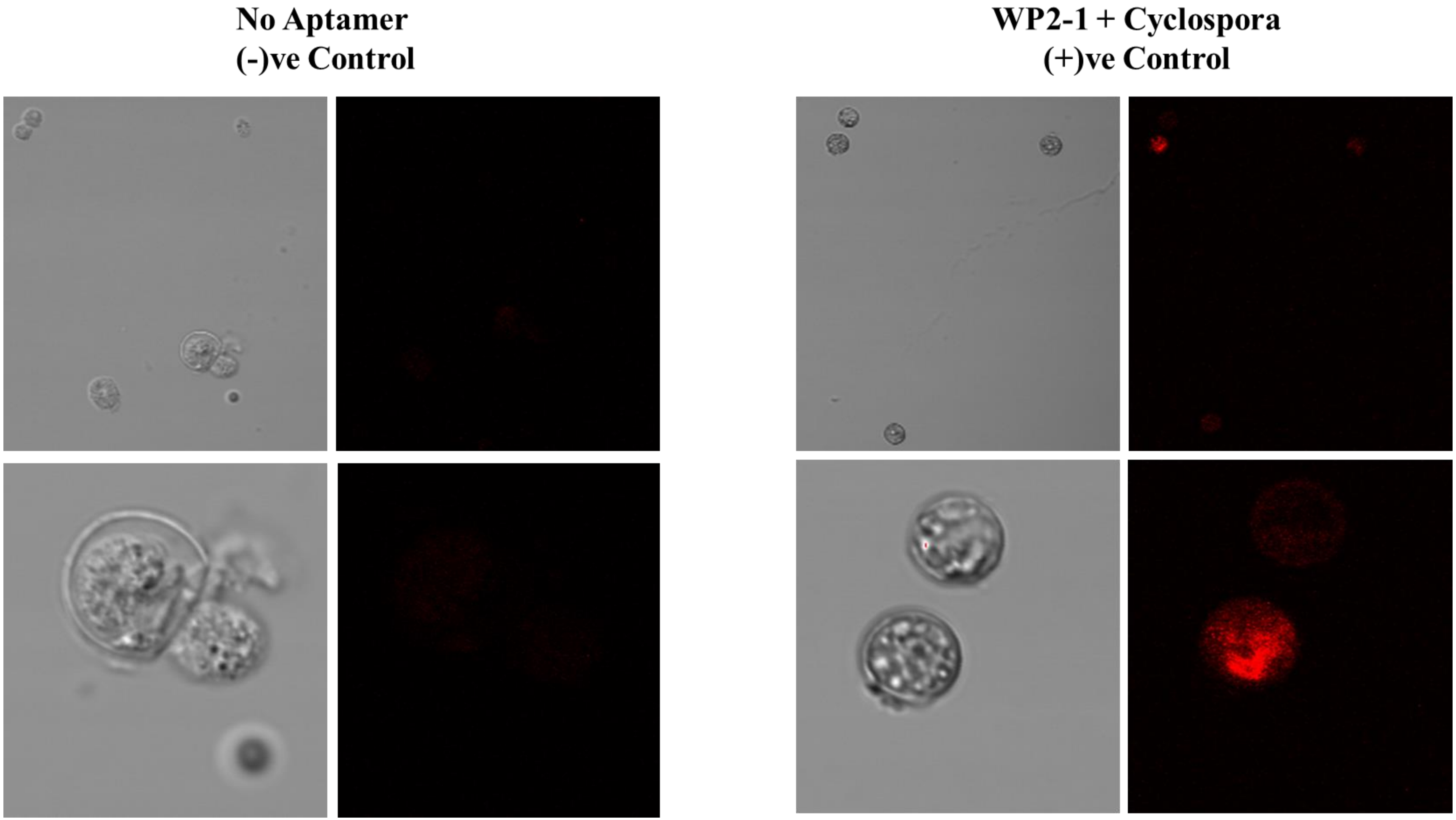

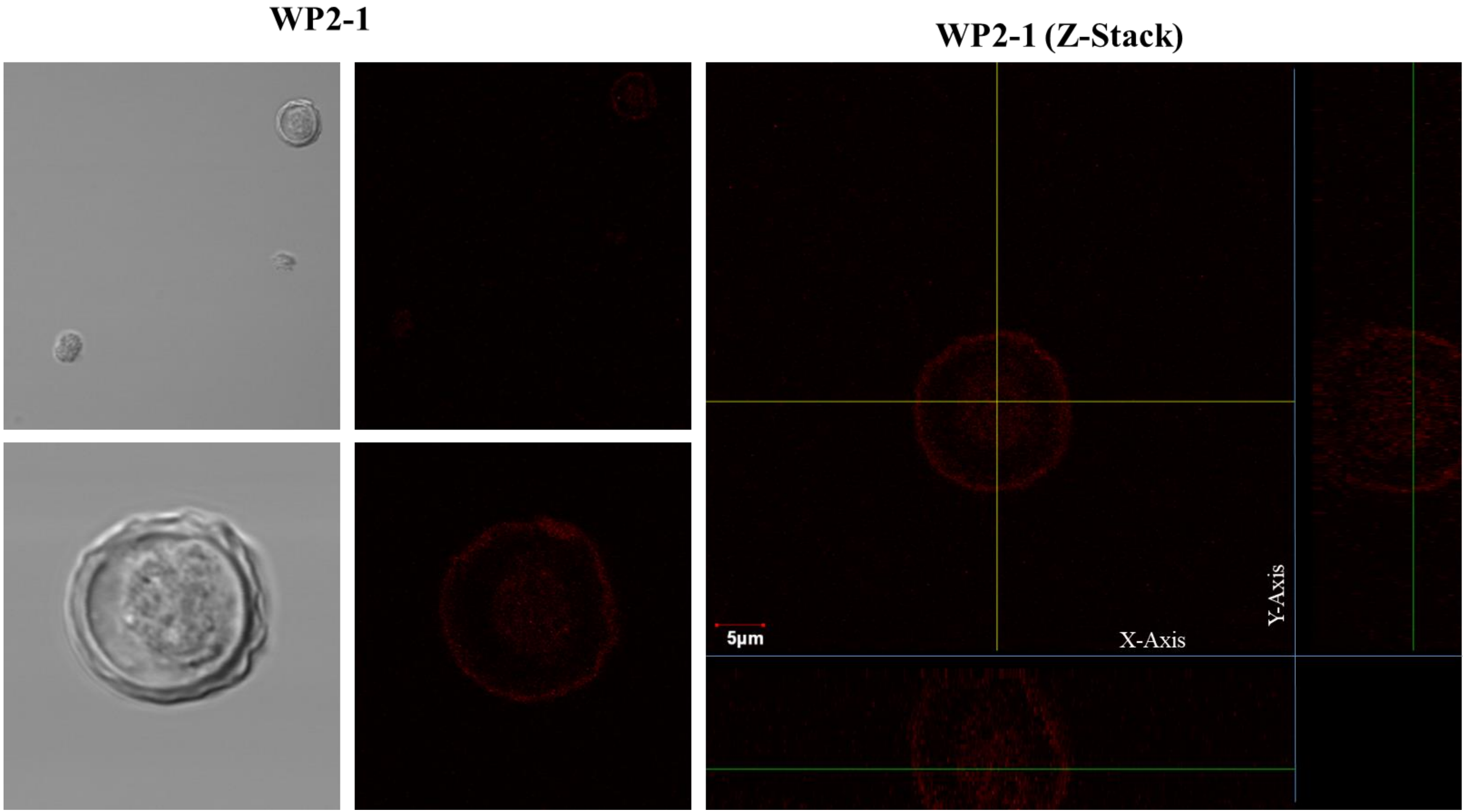

WP2-1
WP2-1 (Z-Stack)
5μm
X-Axis
Y-Axis

Conclusion: Acanthamoeba species showed weak positive signals with most of the aptamers. WP2-1 and WP2-2 aptamers showed a slight increase in signals over other aptamers.

Balamuthia Mandrillaris

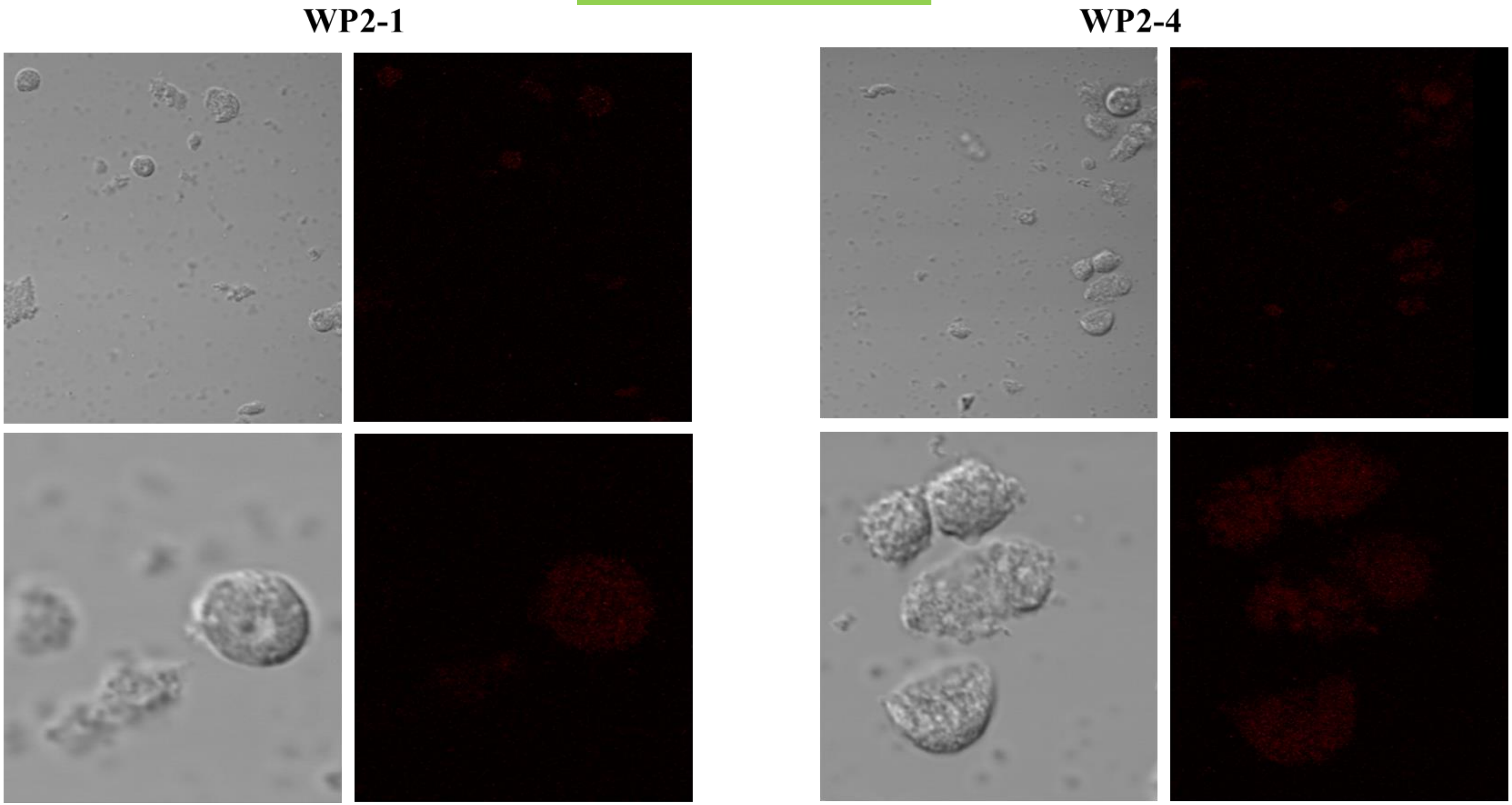

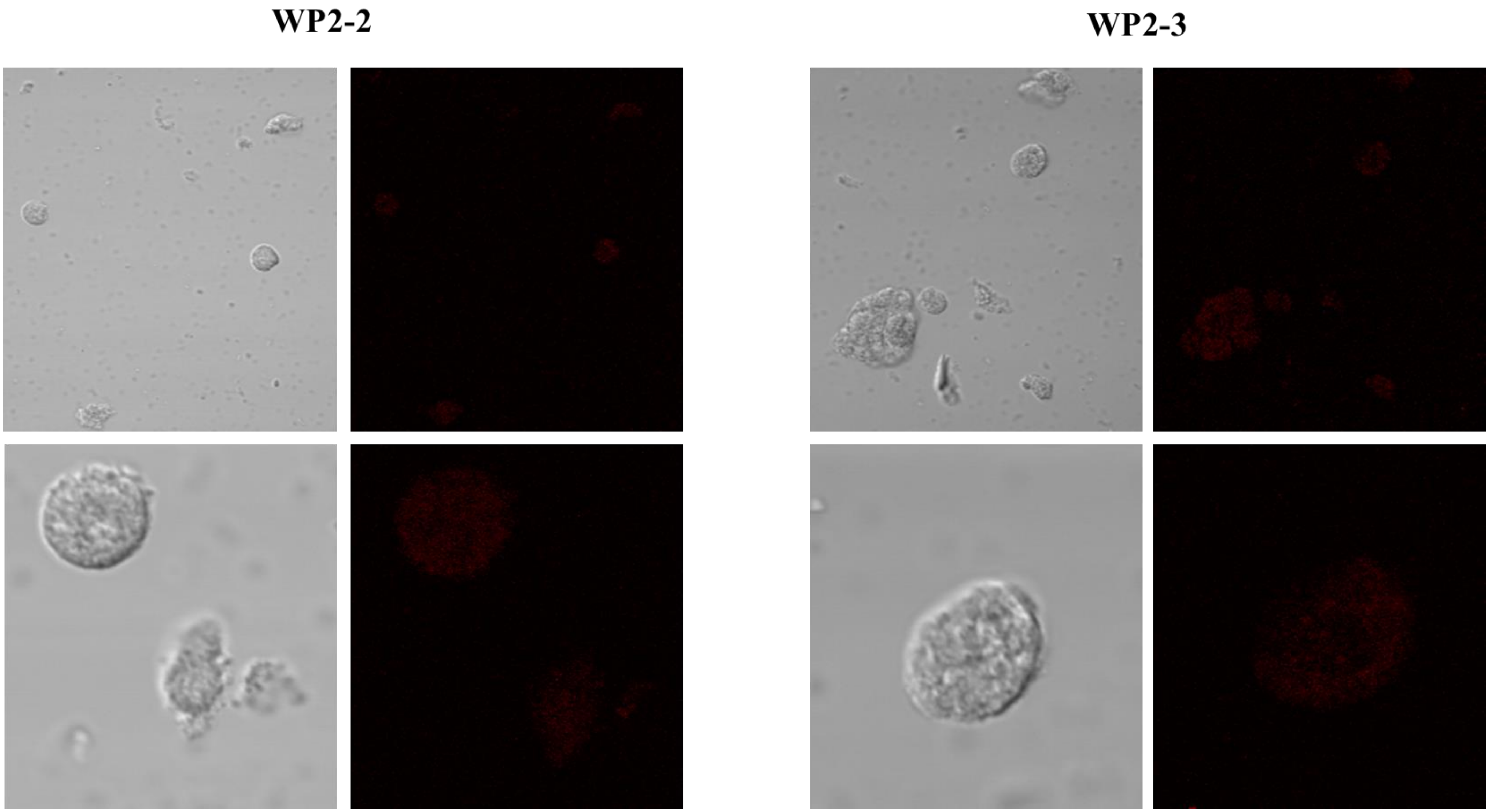
WP2-2
WP2-3

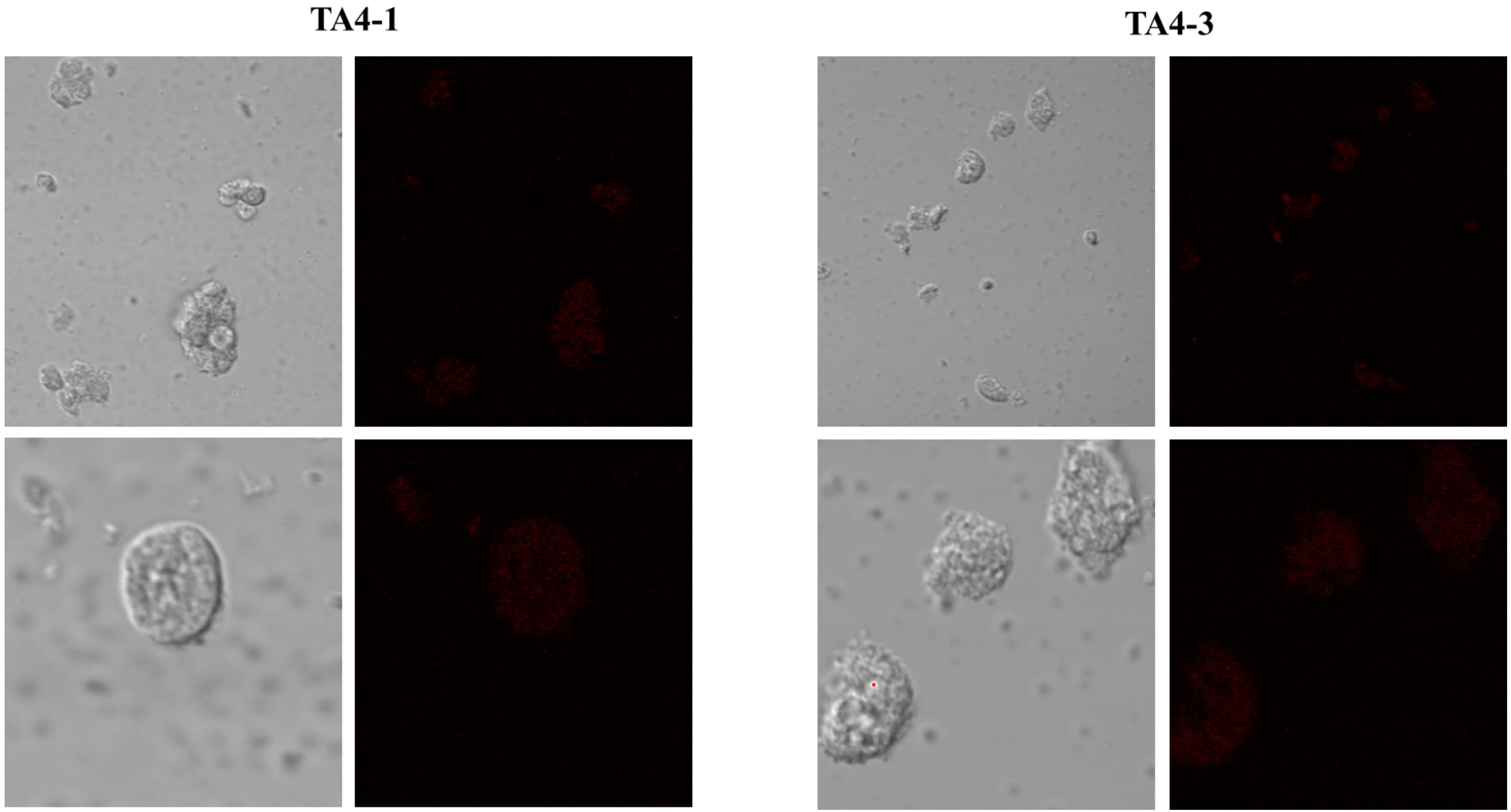
TA4-1
TA4-3

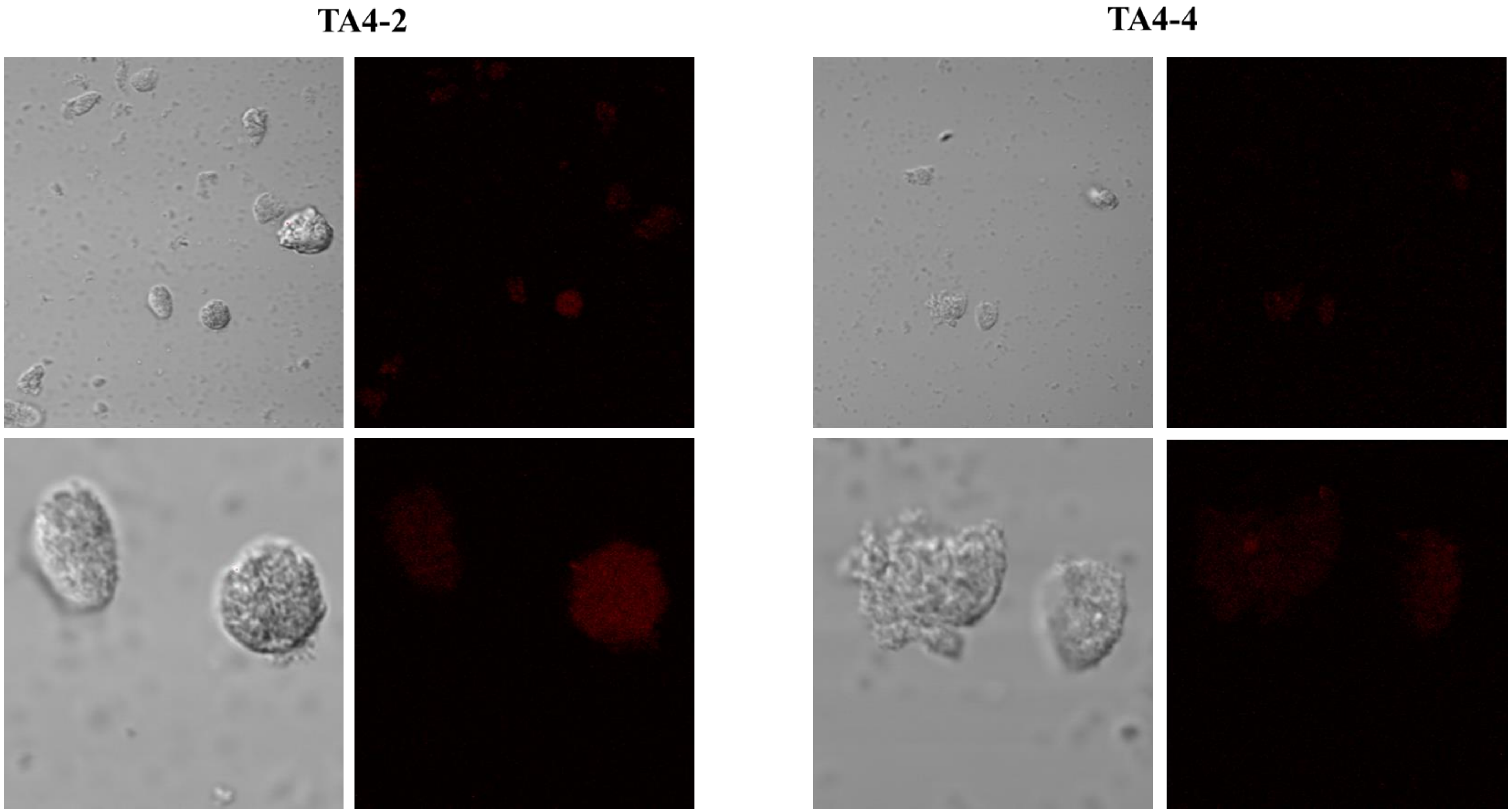
TA4-2
TA4-4

Whole Cyclo S16

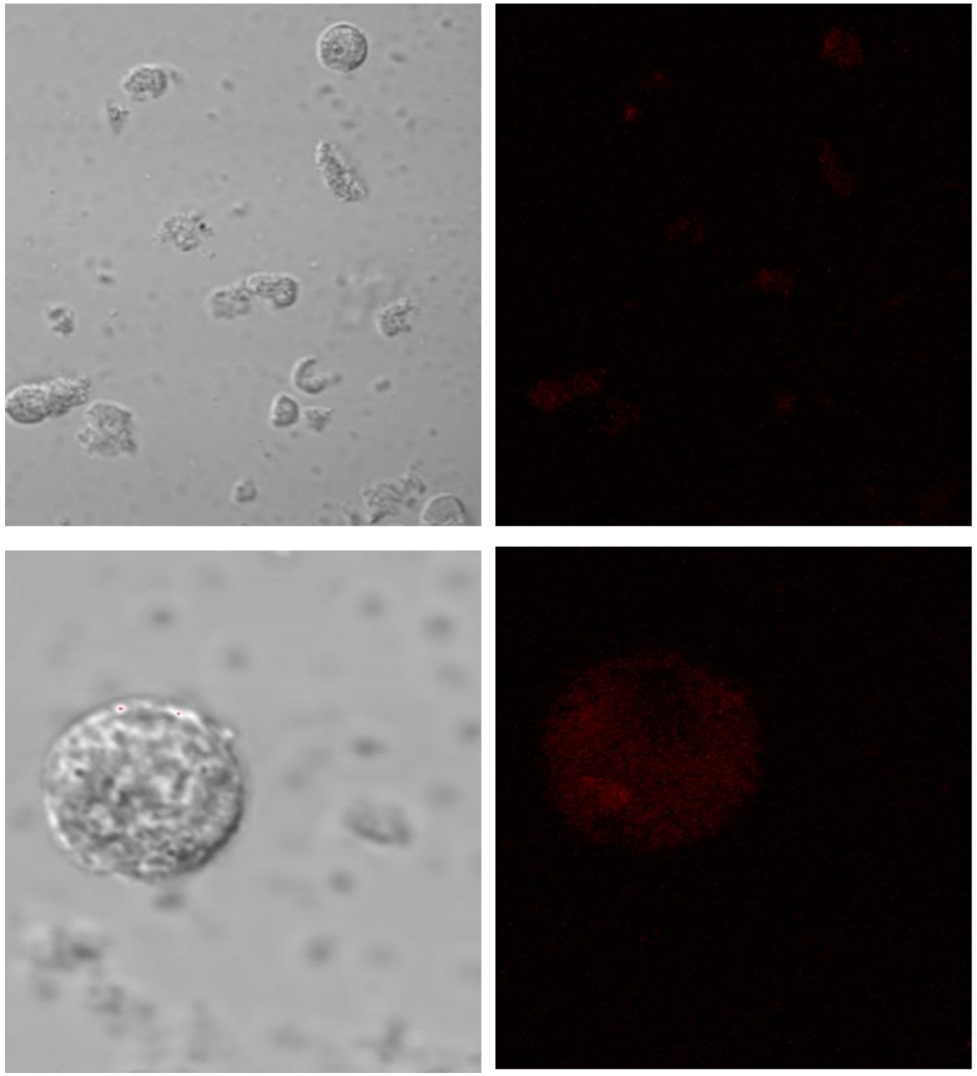

Whole Cyclo S3

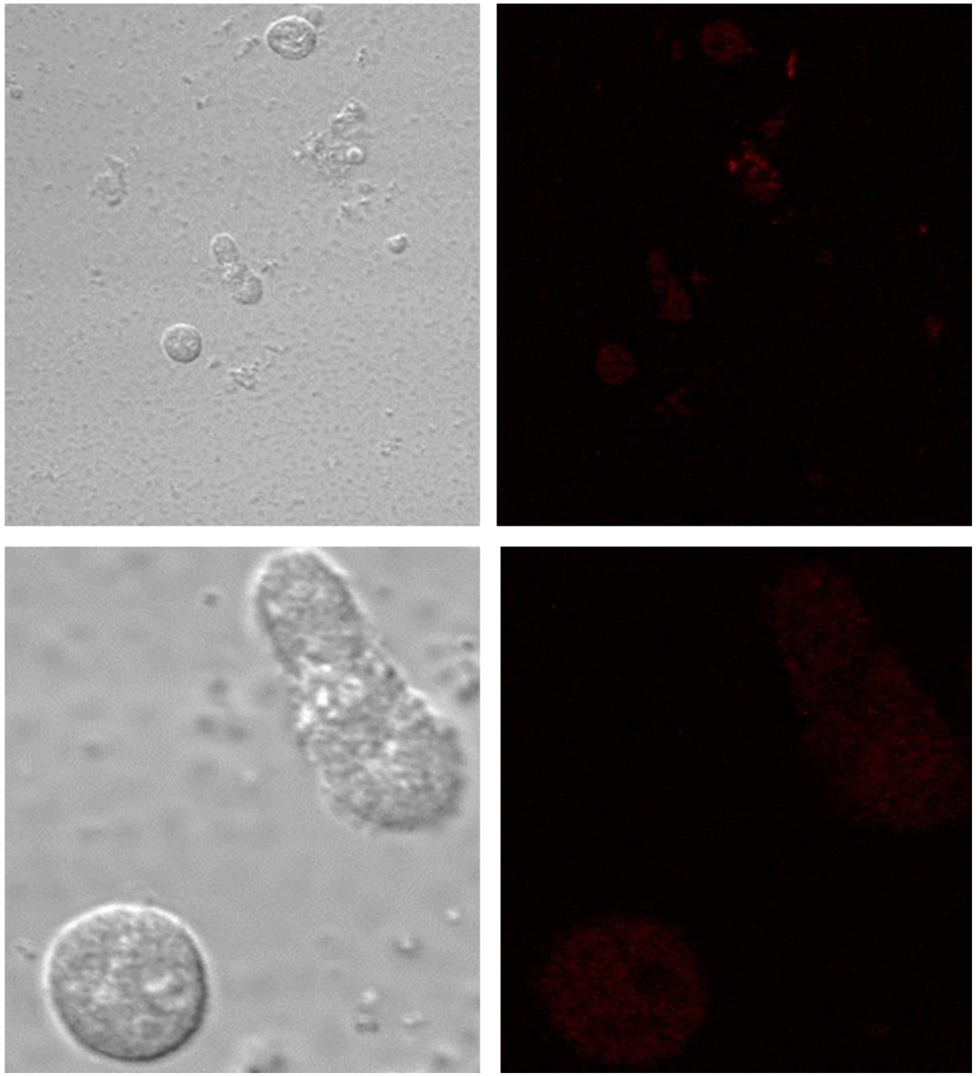

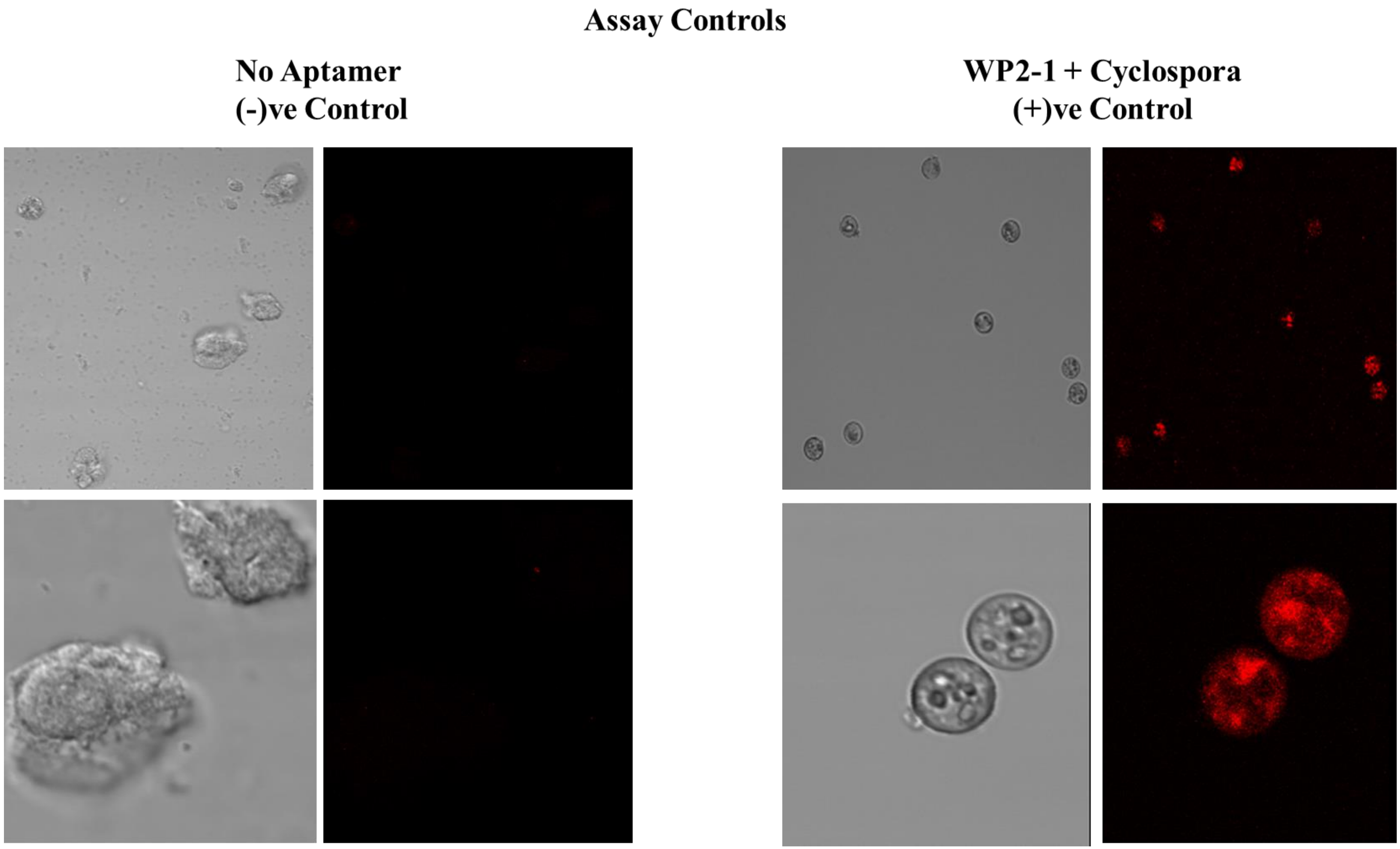
Assay Controls
No Aptamer
(-)ve Control
WP2-1 + Cyclospora
(+)ve Control

TA4-2

TA4-2 (Z-stack)

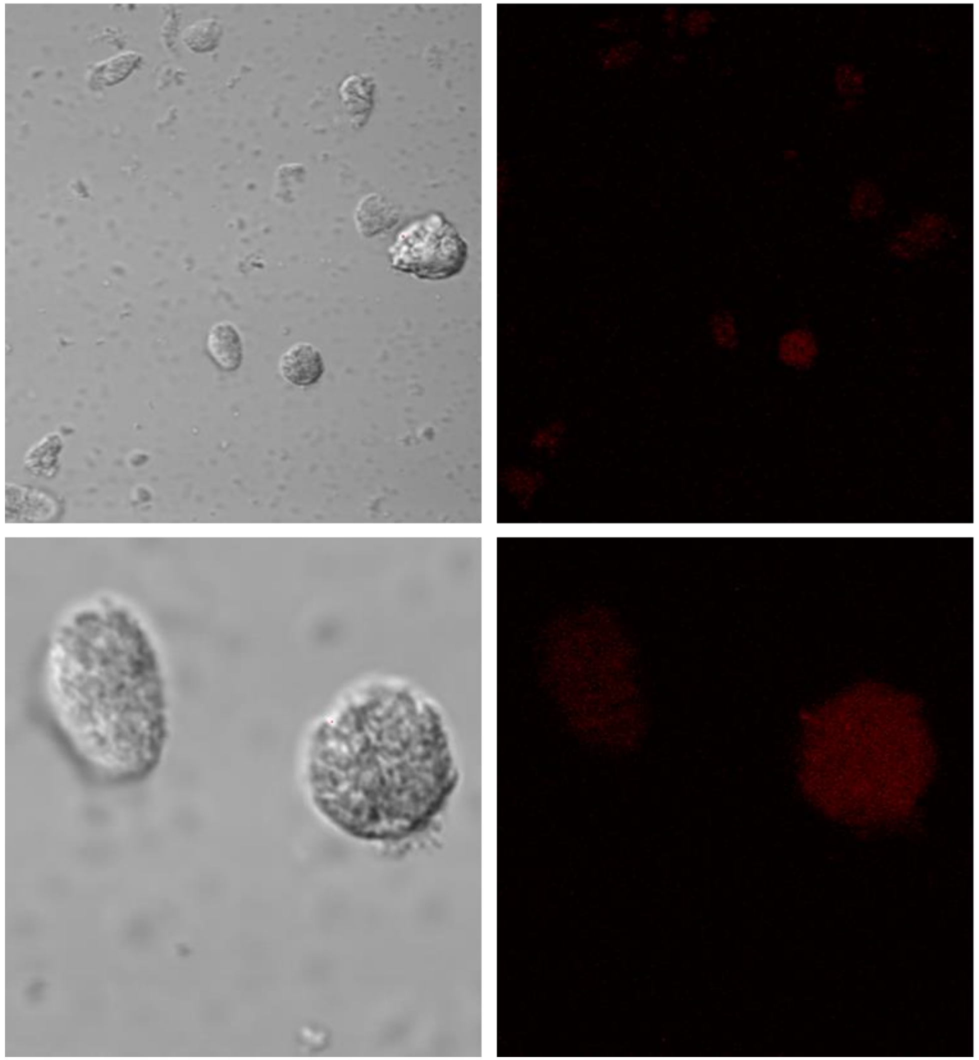

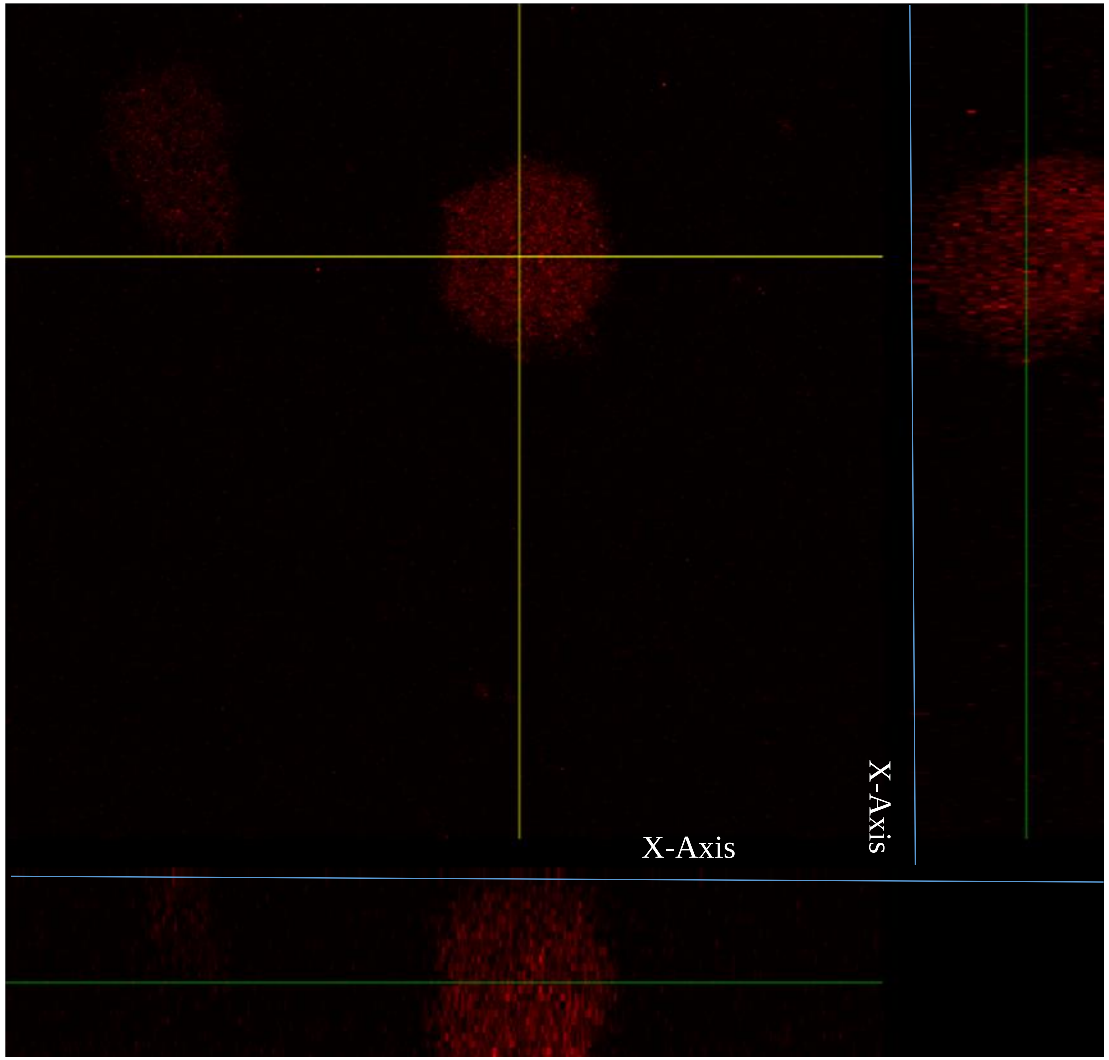

Conclusion: Balamuthia mandurillaris showed weak or insignificant positive signals.

Cryptosporidium Parvum

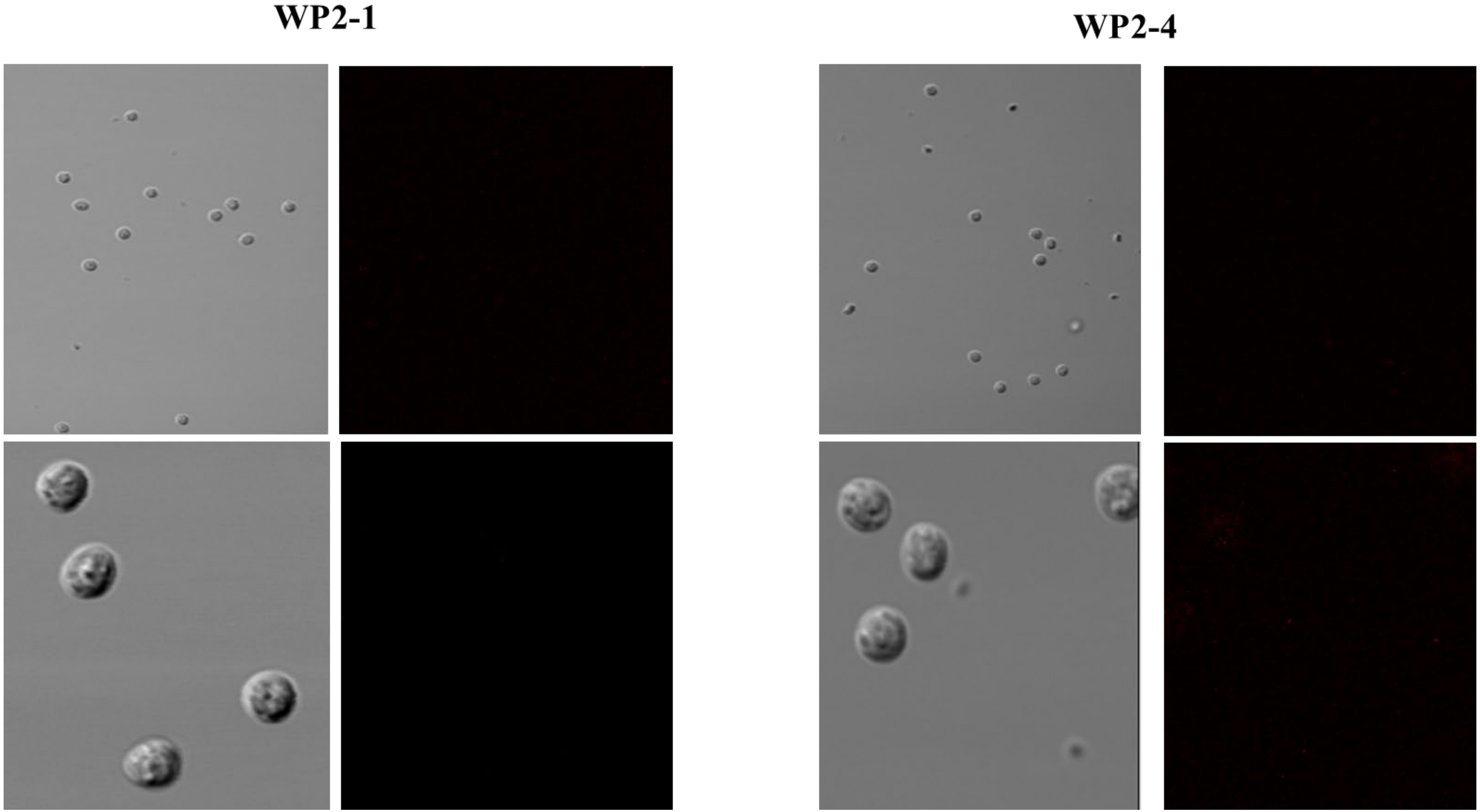

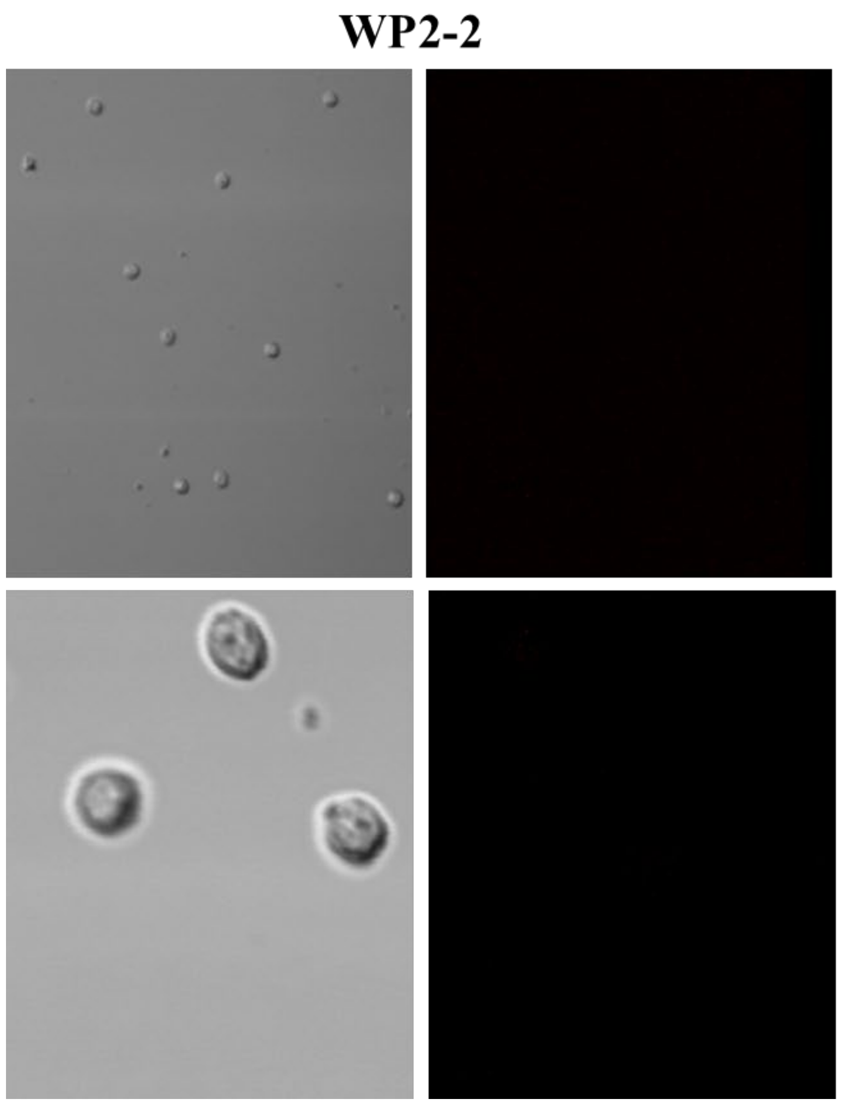
WP2-2
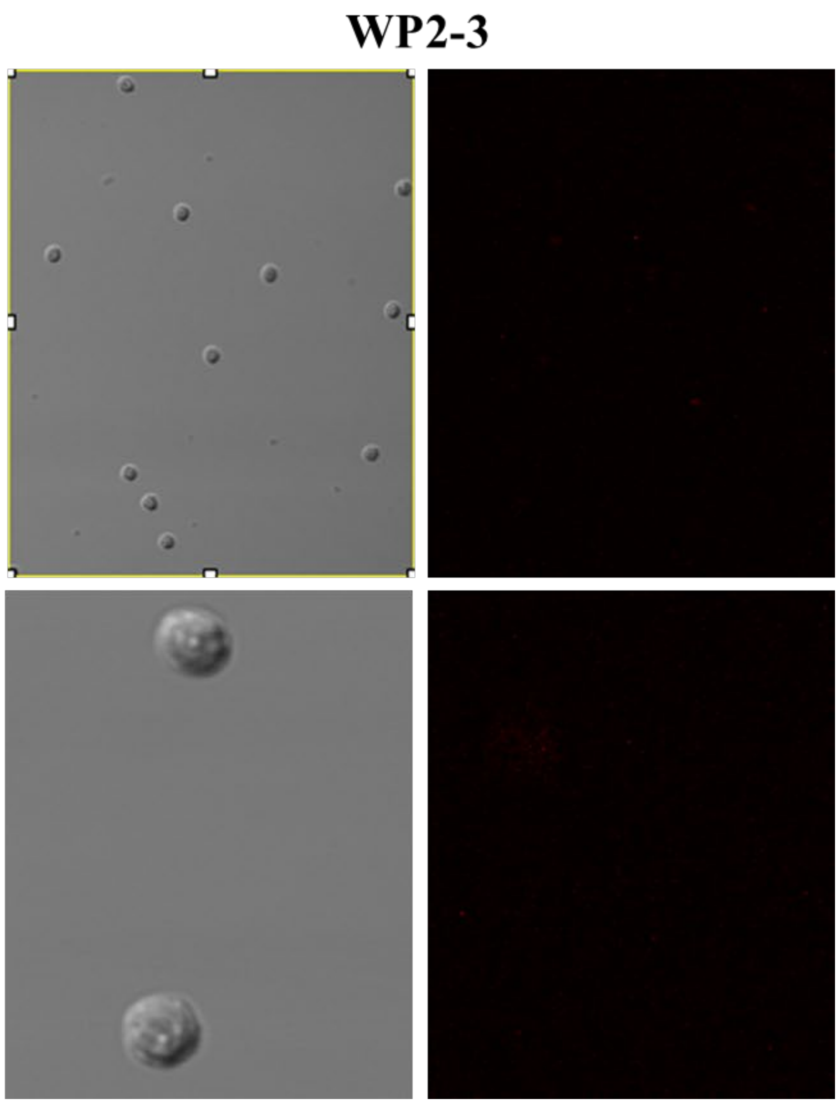
WP2-3

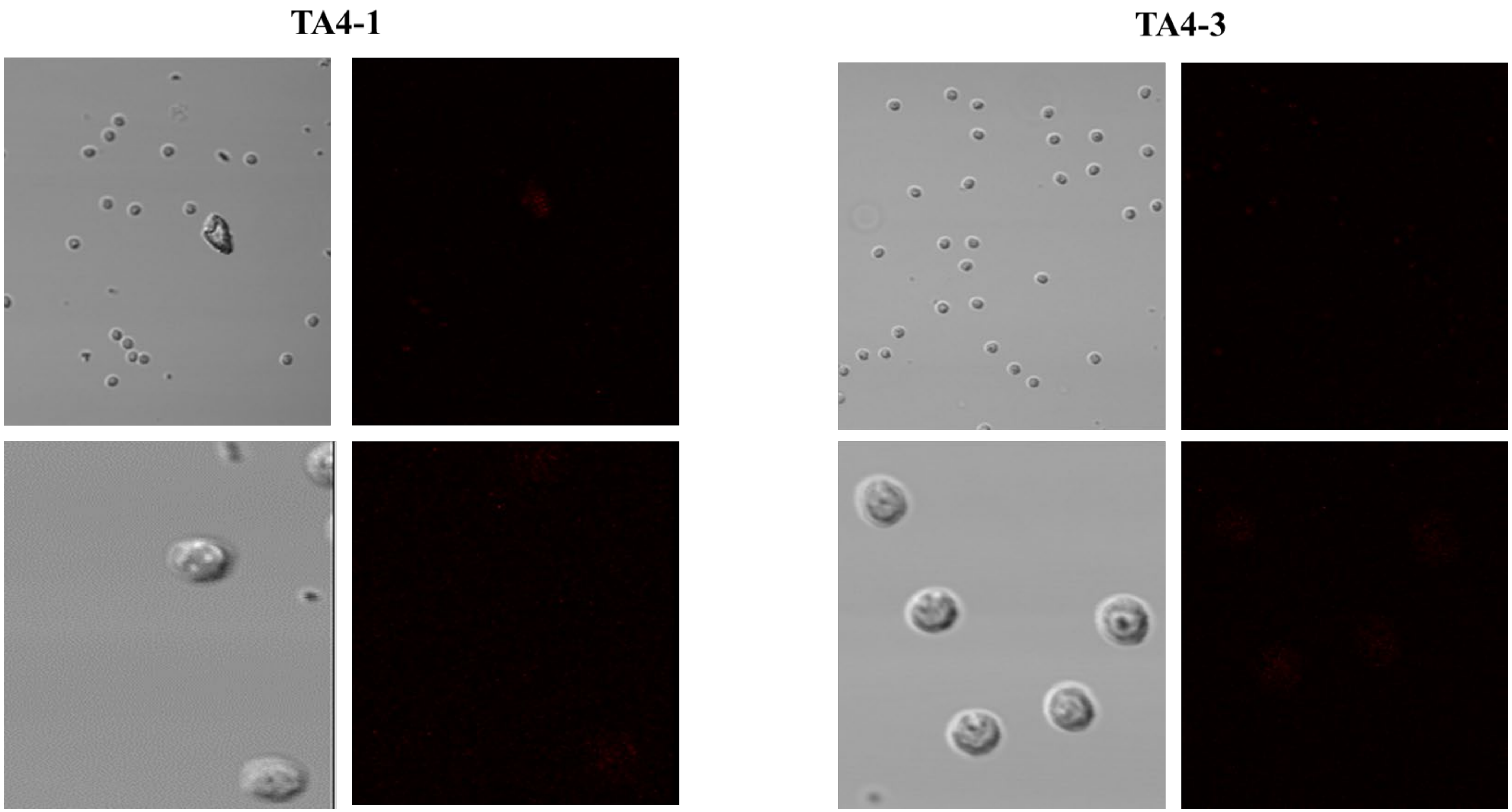
TA4-1
TA4-3

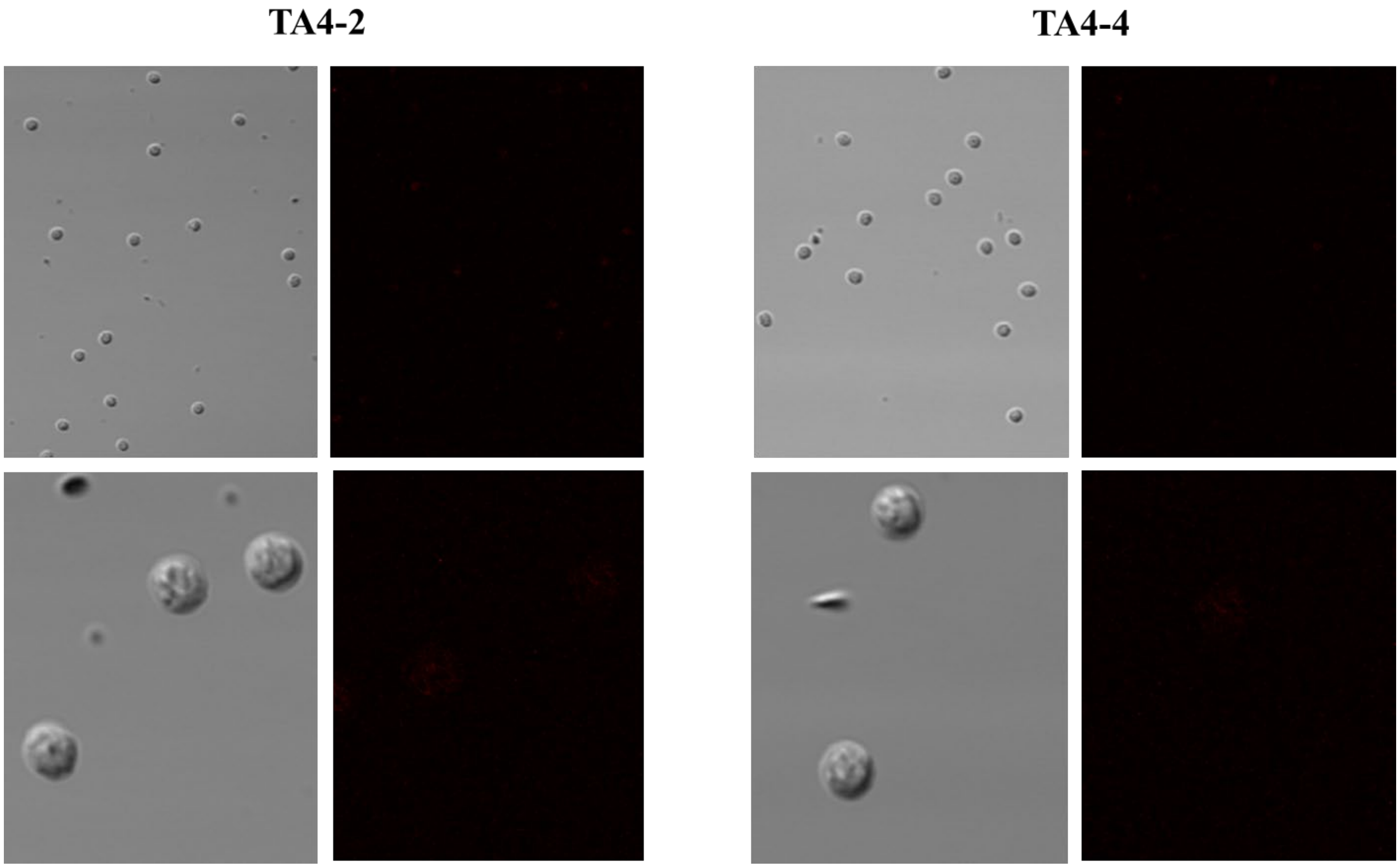

TA4-2
TA4-4

Whole Cyclo S16

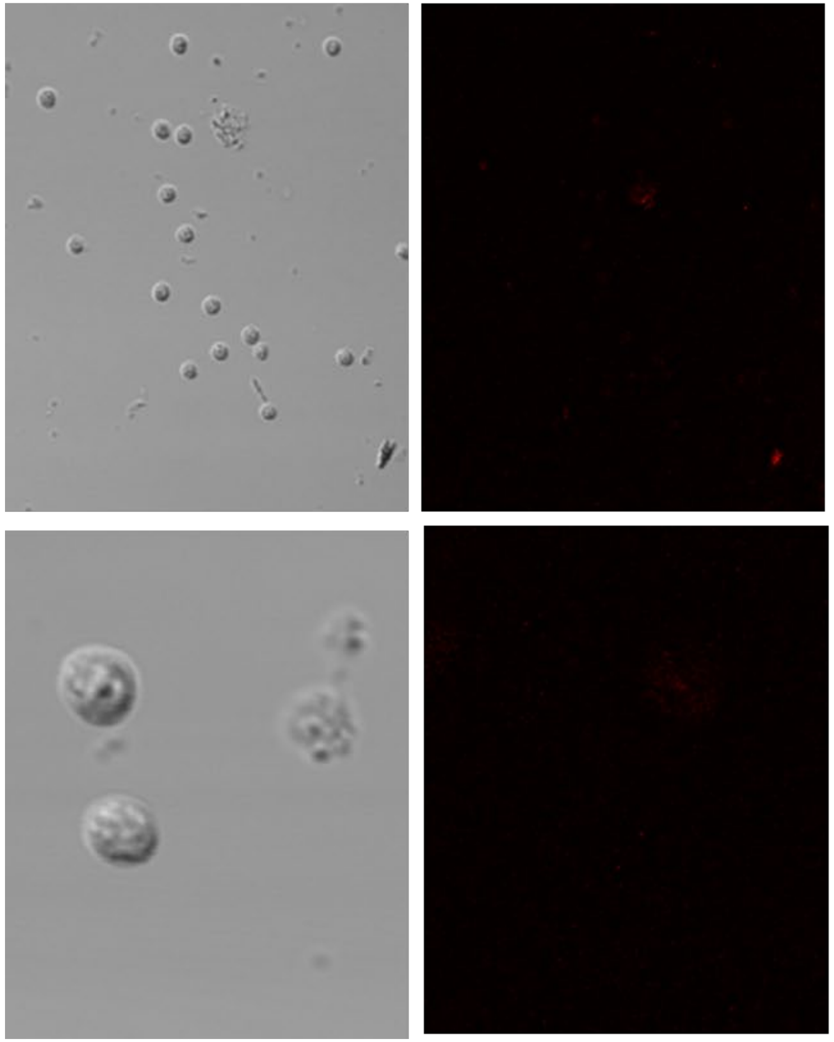

Whole Cyclo S3

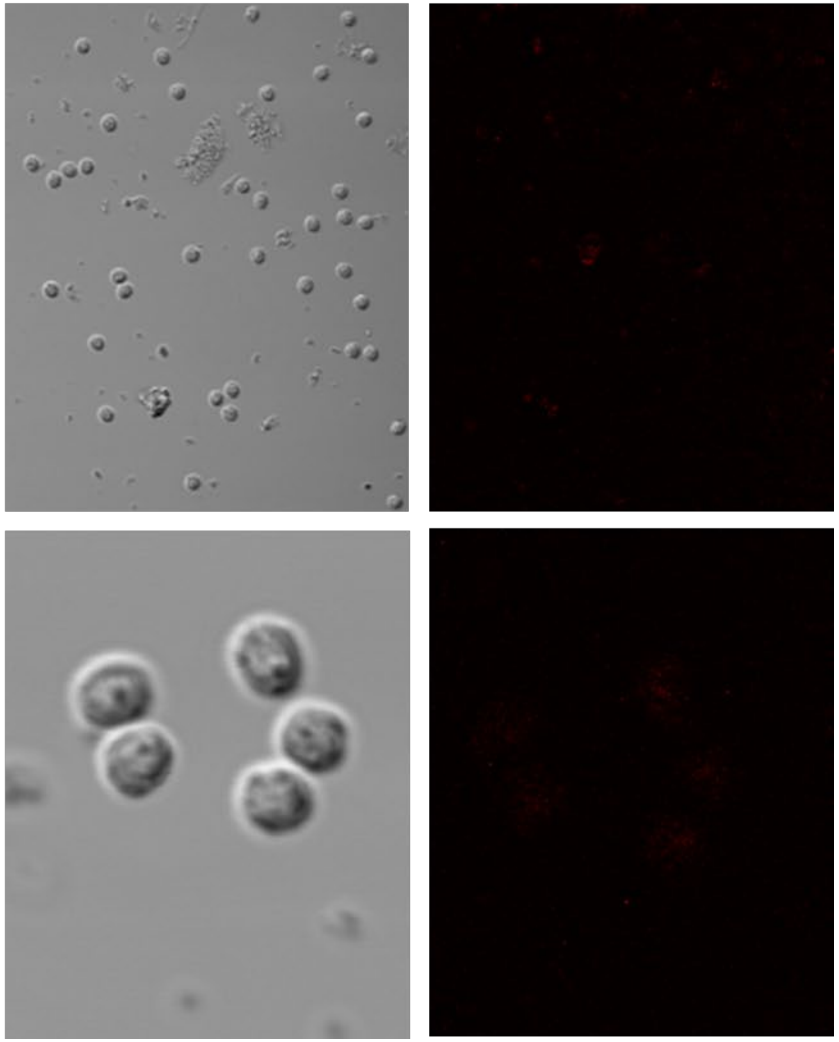

Assay Controls

No Aptamer
(-)ve Control

WP2-1 + Cyclospora
(+)ve Control

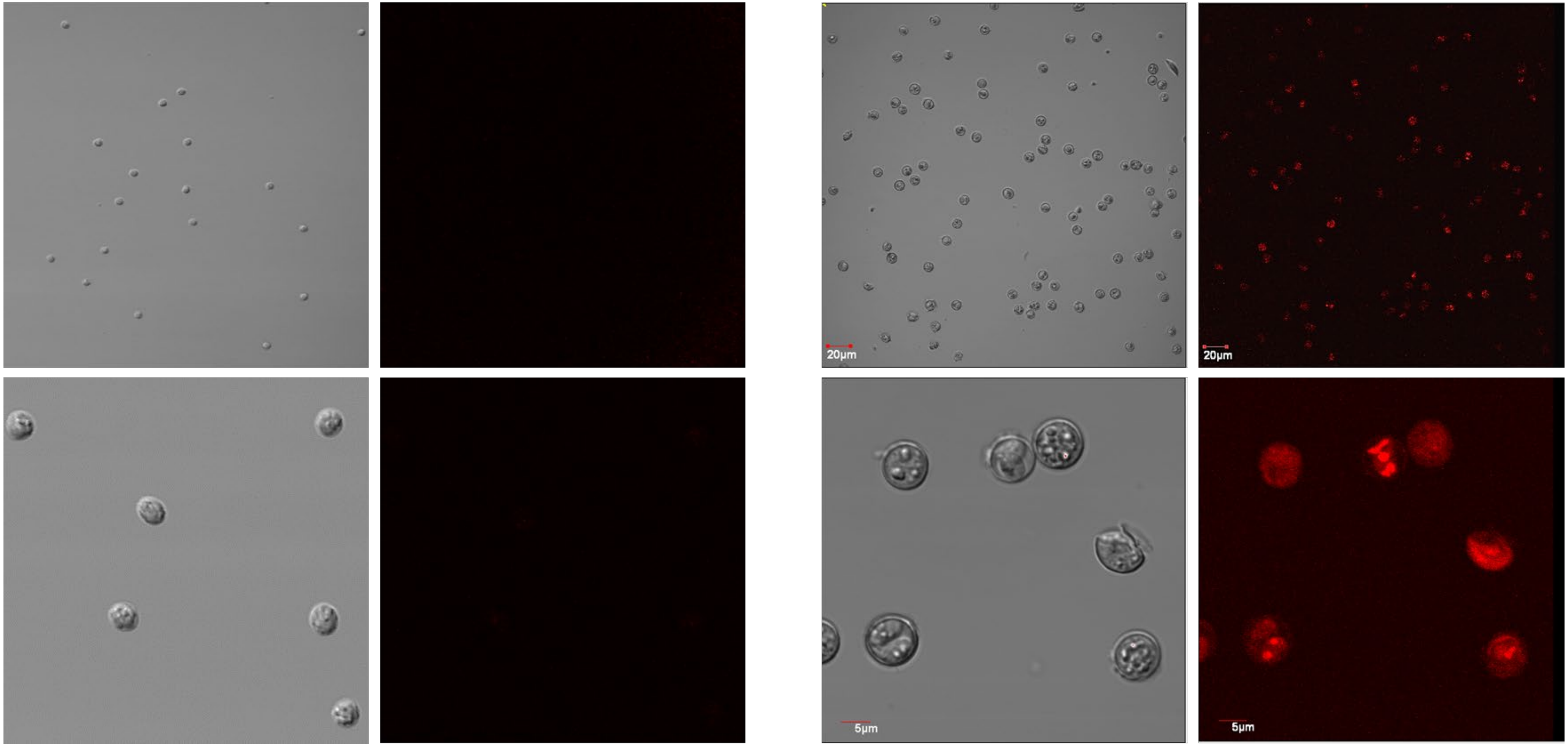

Conclusion: Cryptosporidium parvum showed no positive signals to any of the aptamer

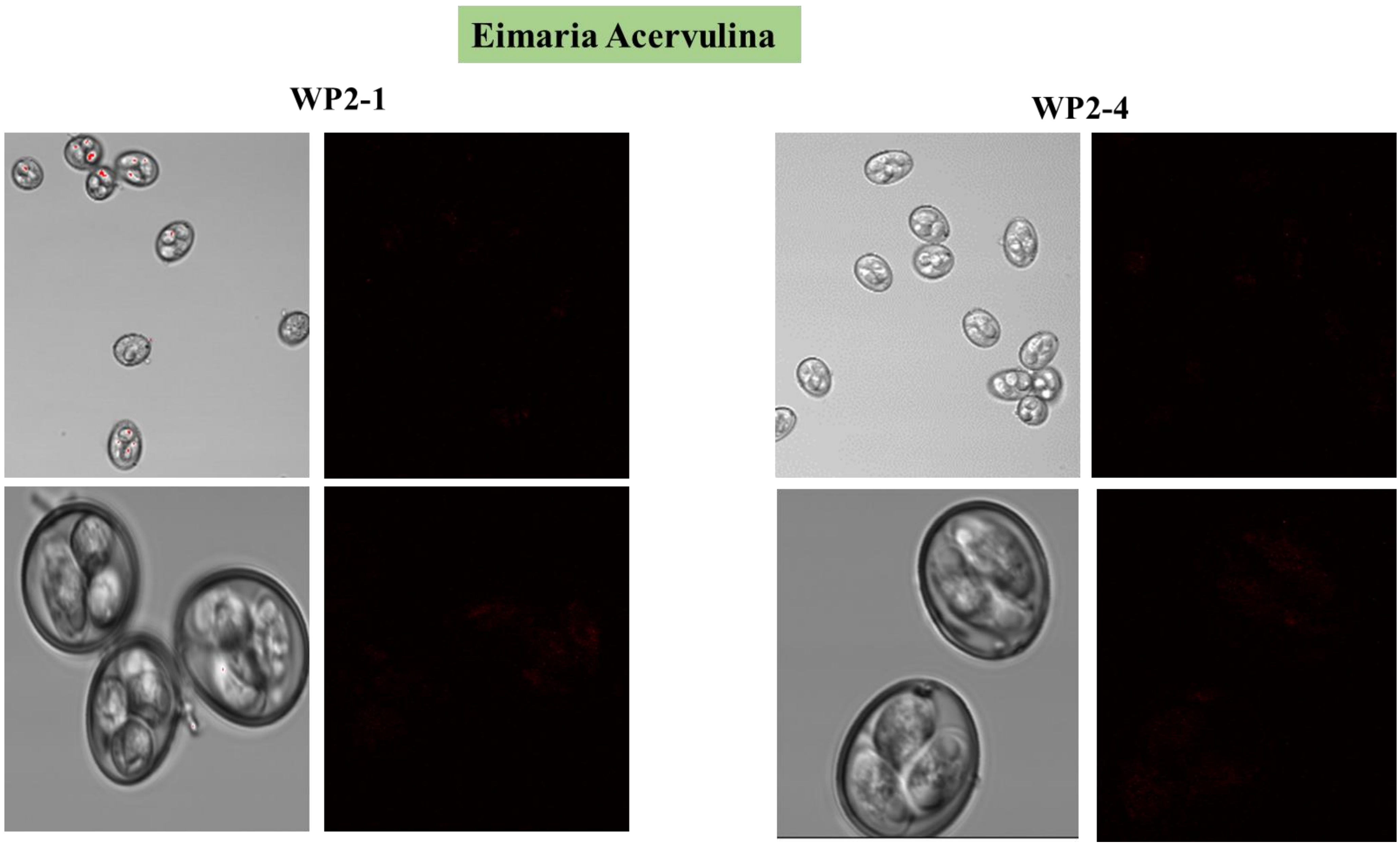
Eimaria Acervulina
WP2-1
WP2-4

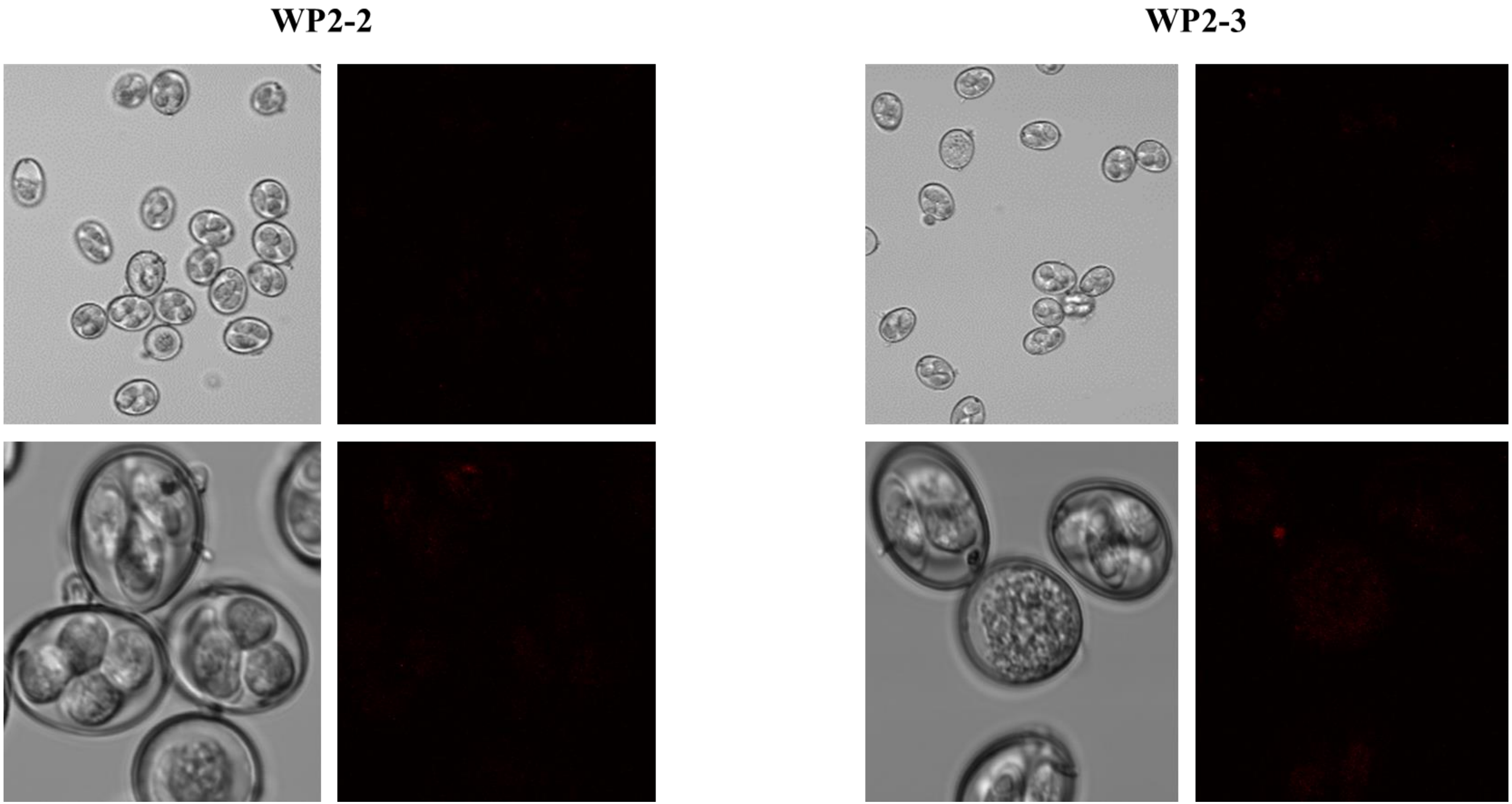
WP2-2
WP2-3

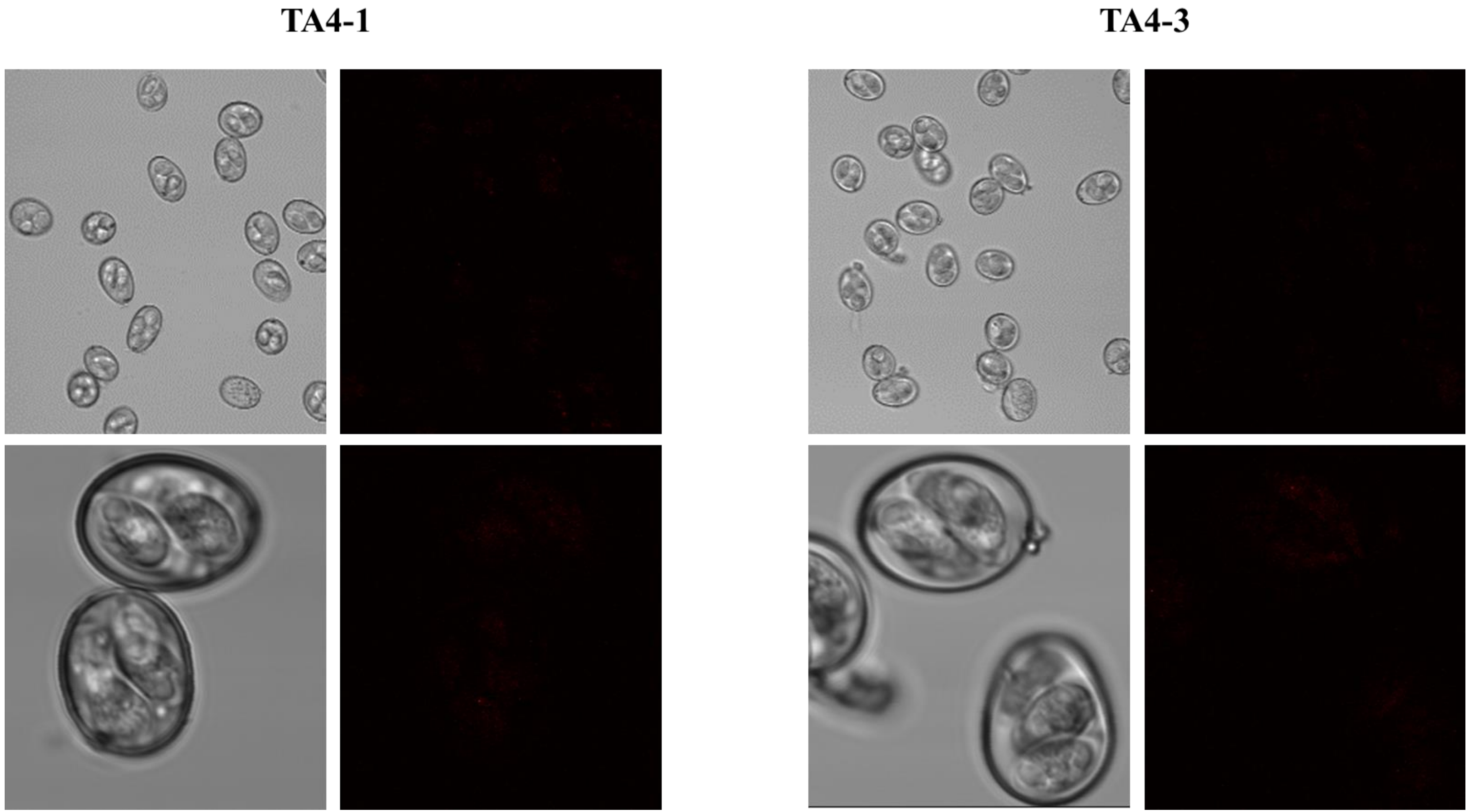
TA4-1
TA4-3

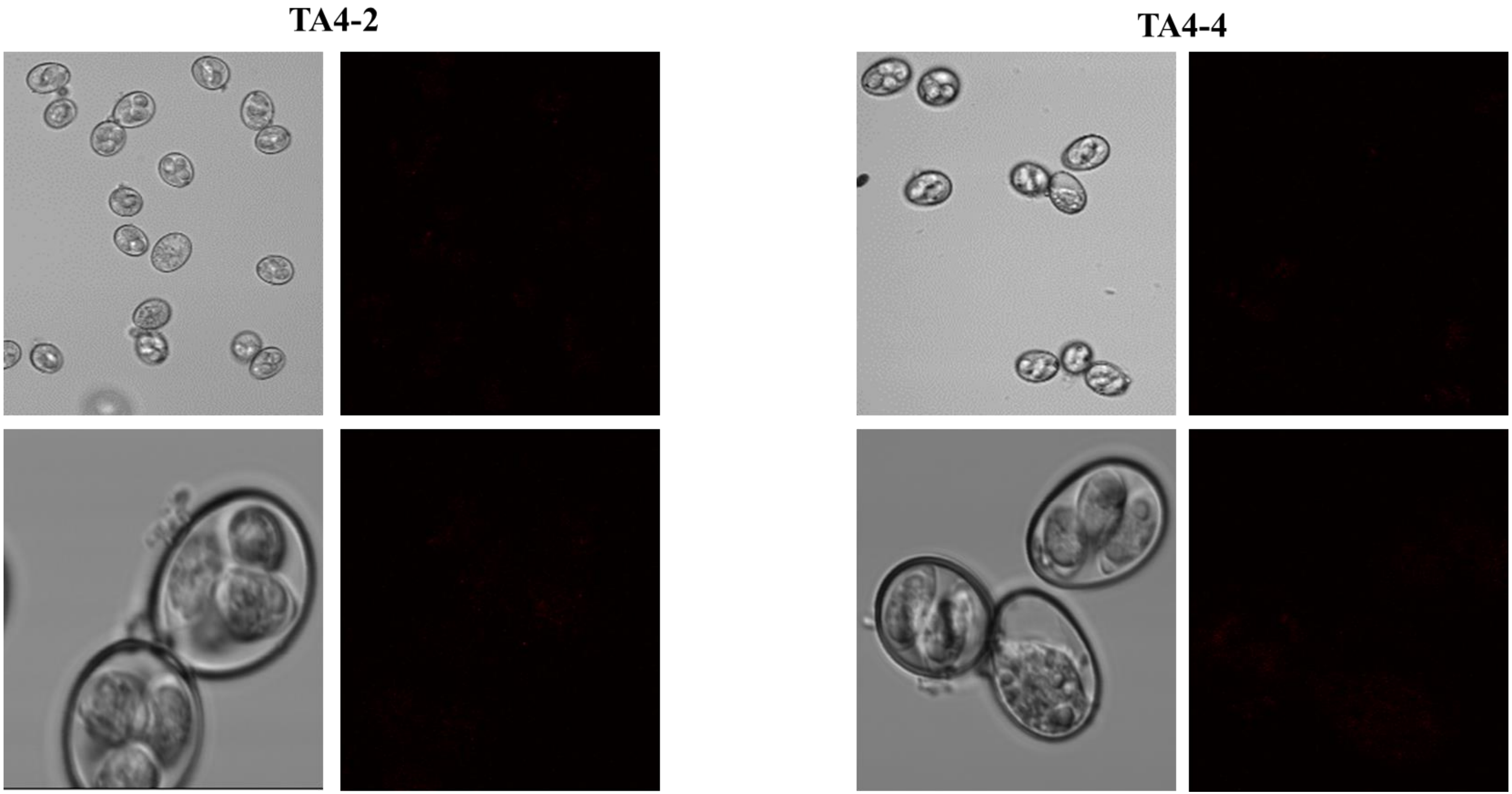
TA4-2
TA4-4

Whole Cyclo S16 **Whole Cyclo S3**

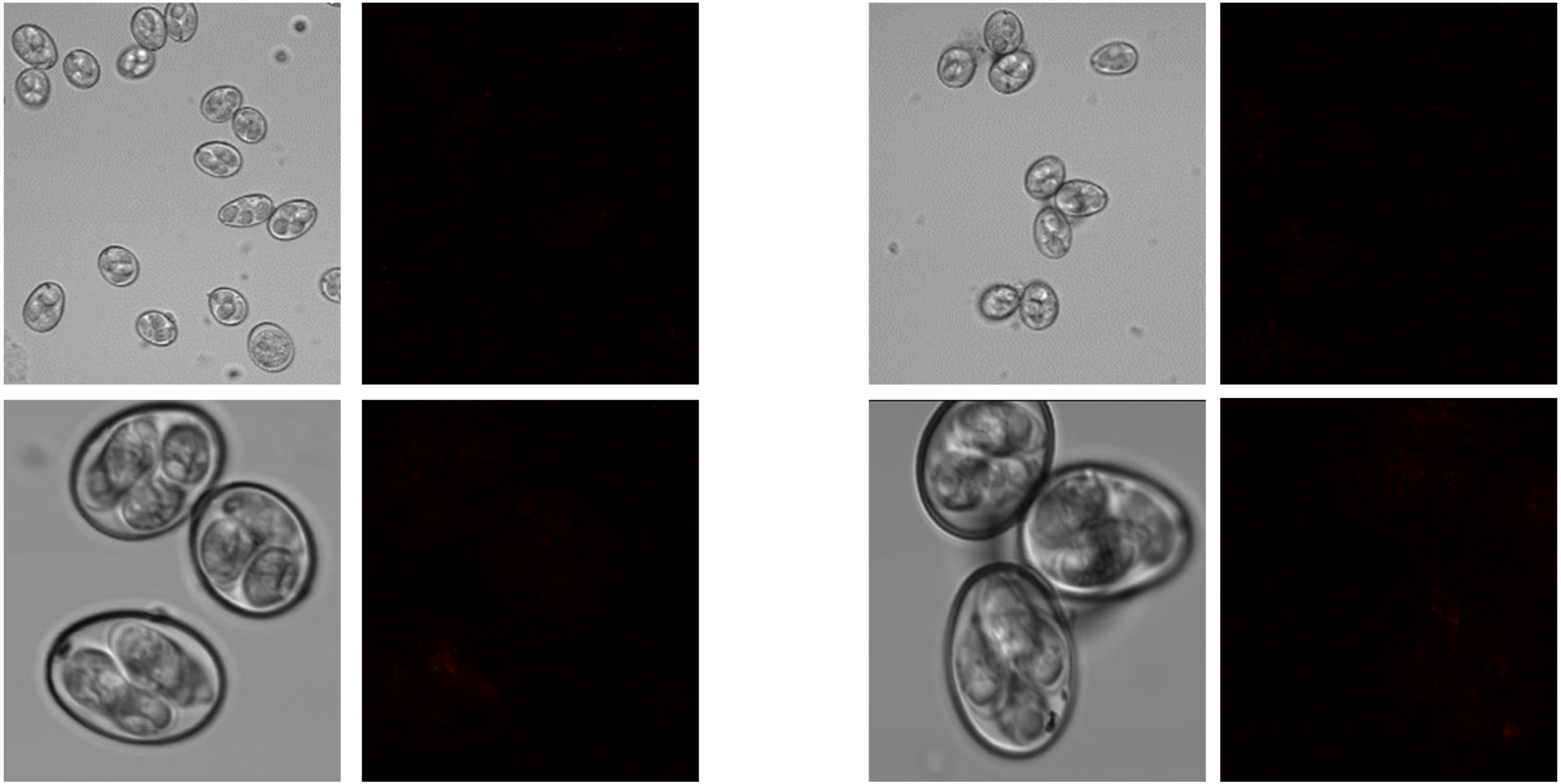

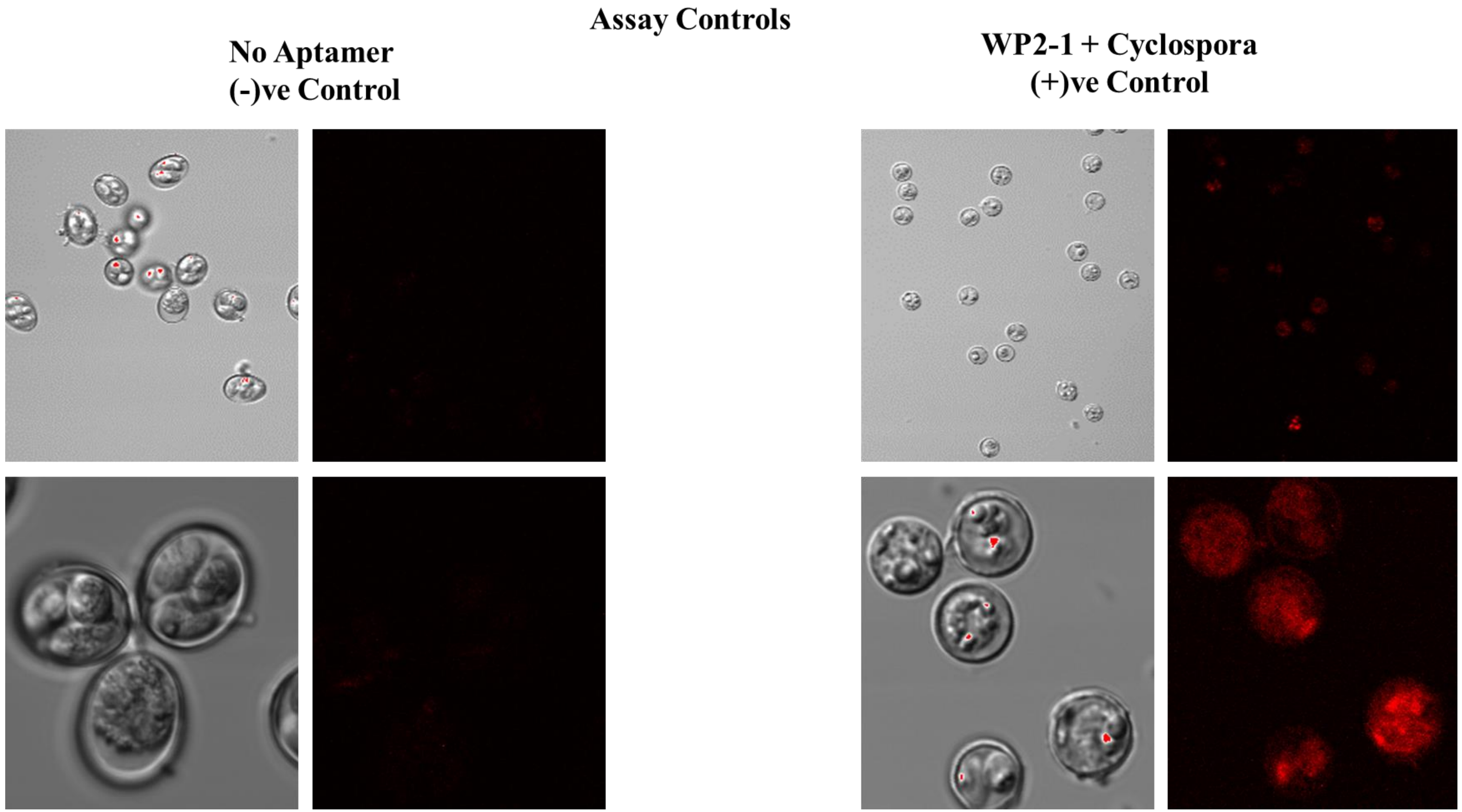
Assay Controls
No Aptamer
(-)ve Control
WP2-1 + Cyclospora
(+)ve Control

Conclusion: Eimaria acervulina showed no positive signals with any of the aptamers.

Eimaria Maxima

WP2-1 **WP2-4**

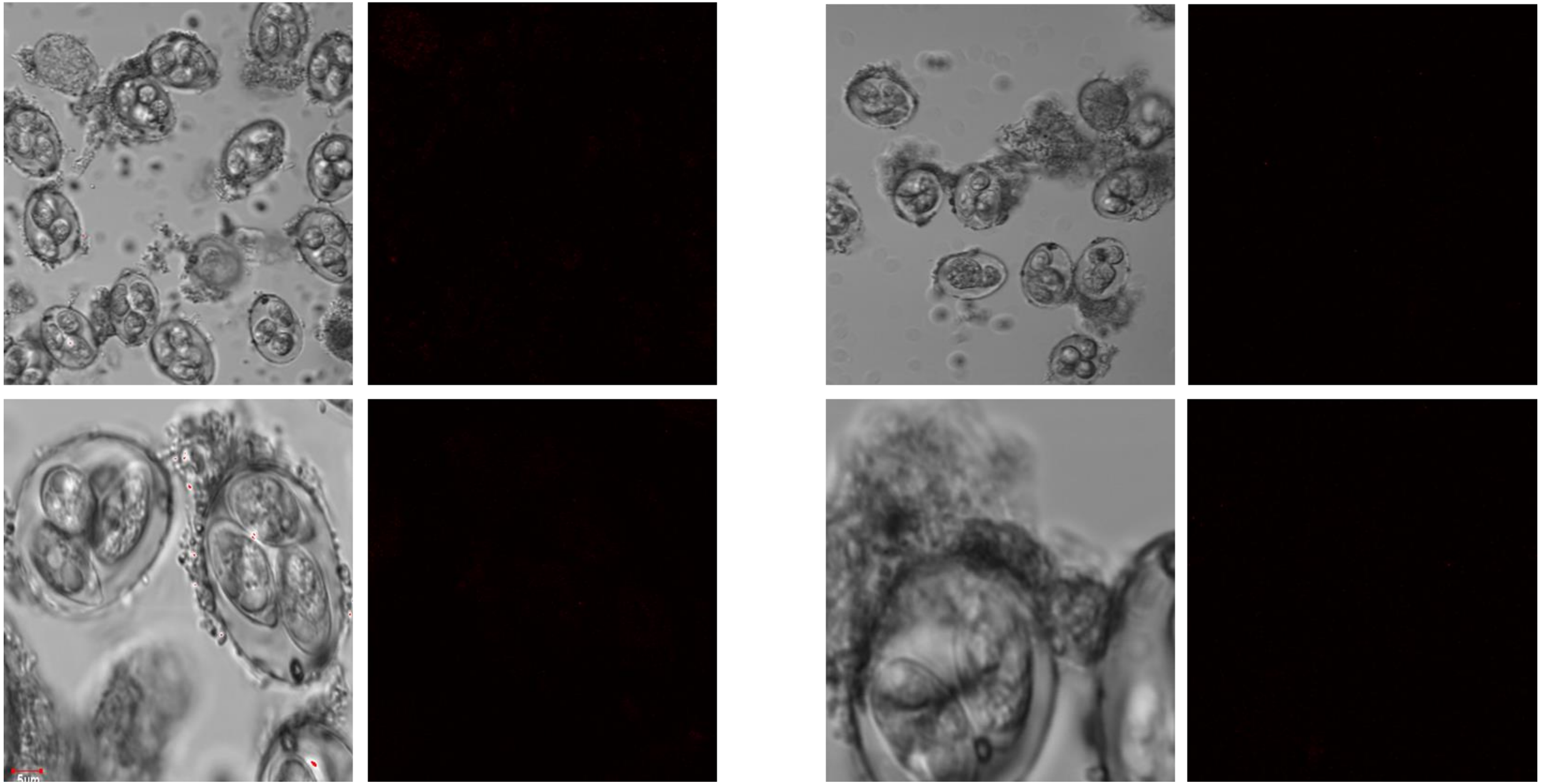

WP2-2

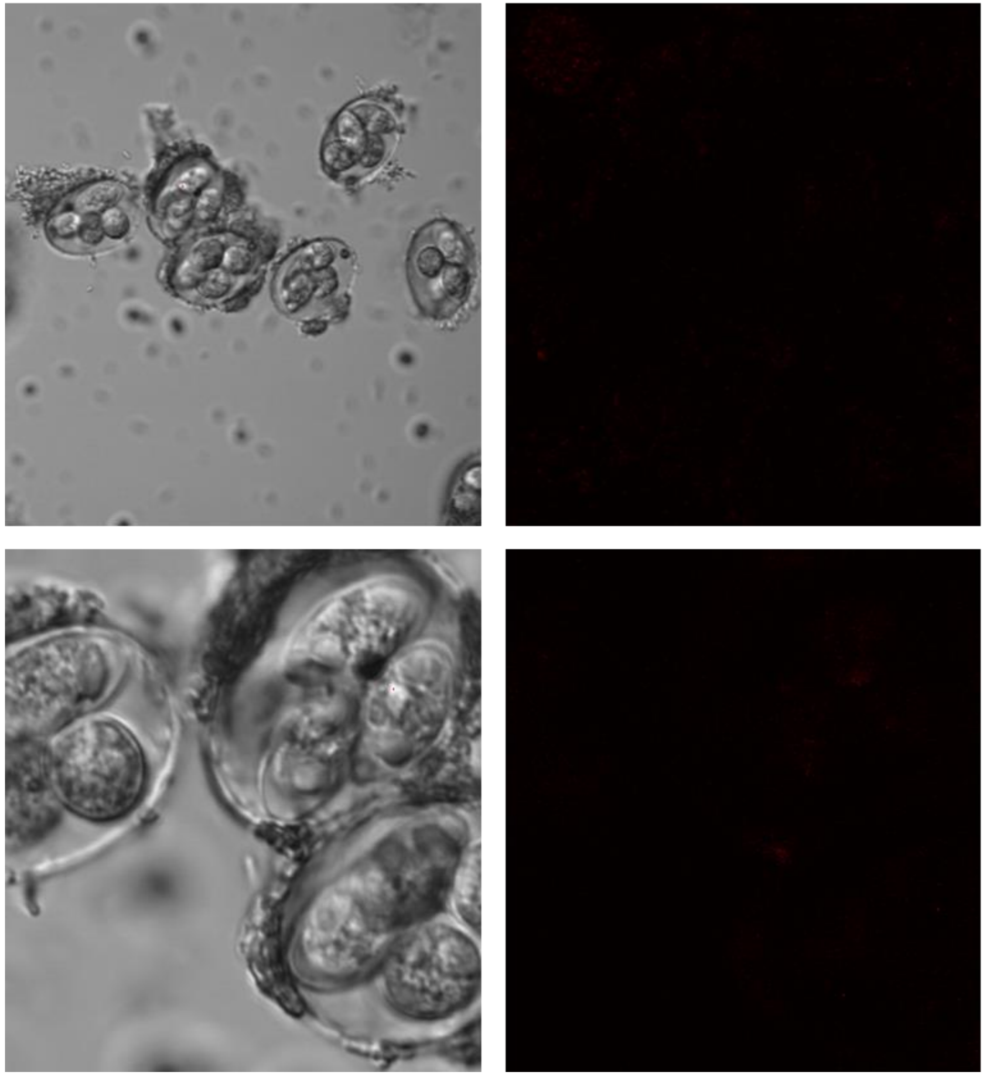

WP2-3

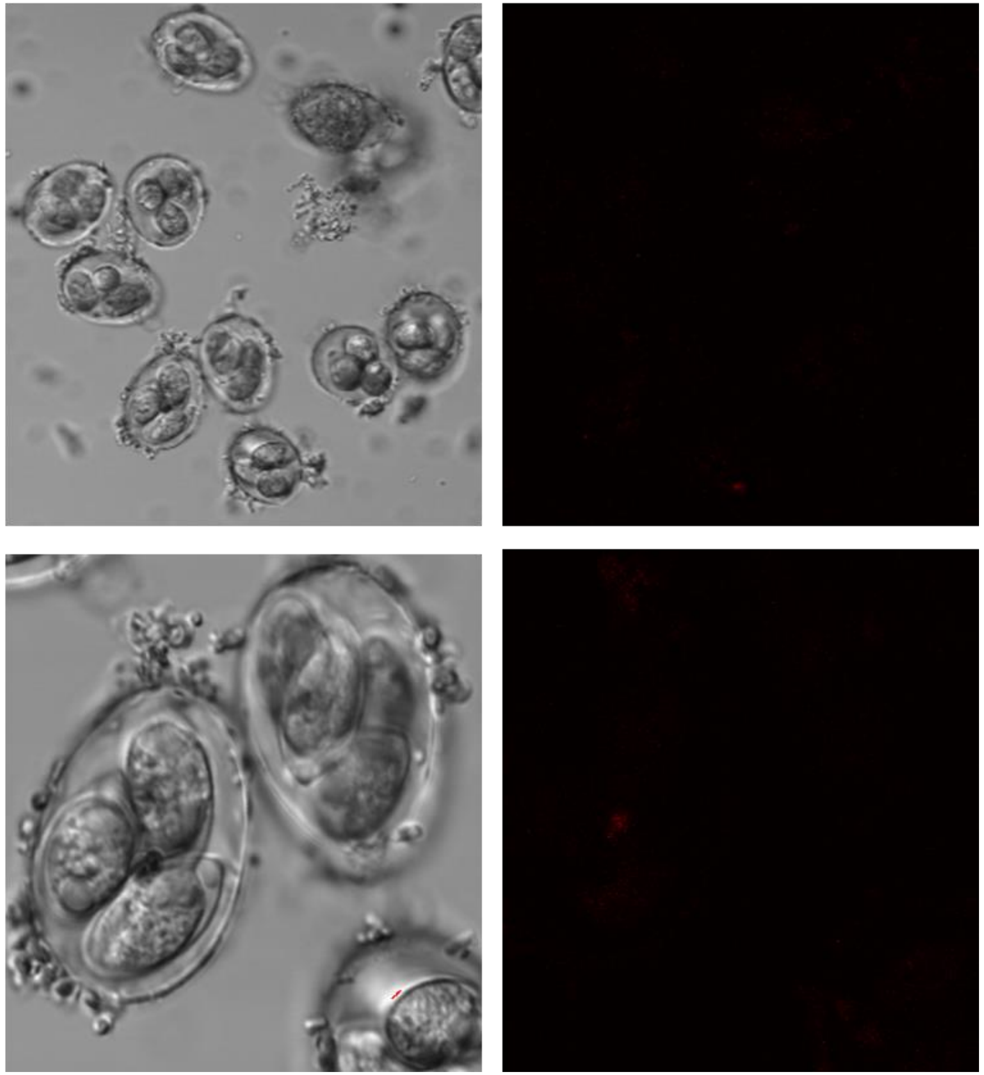

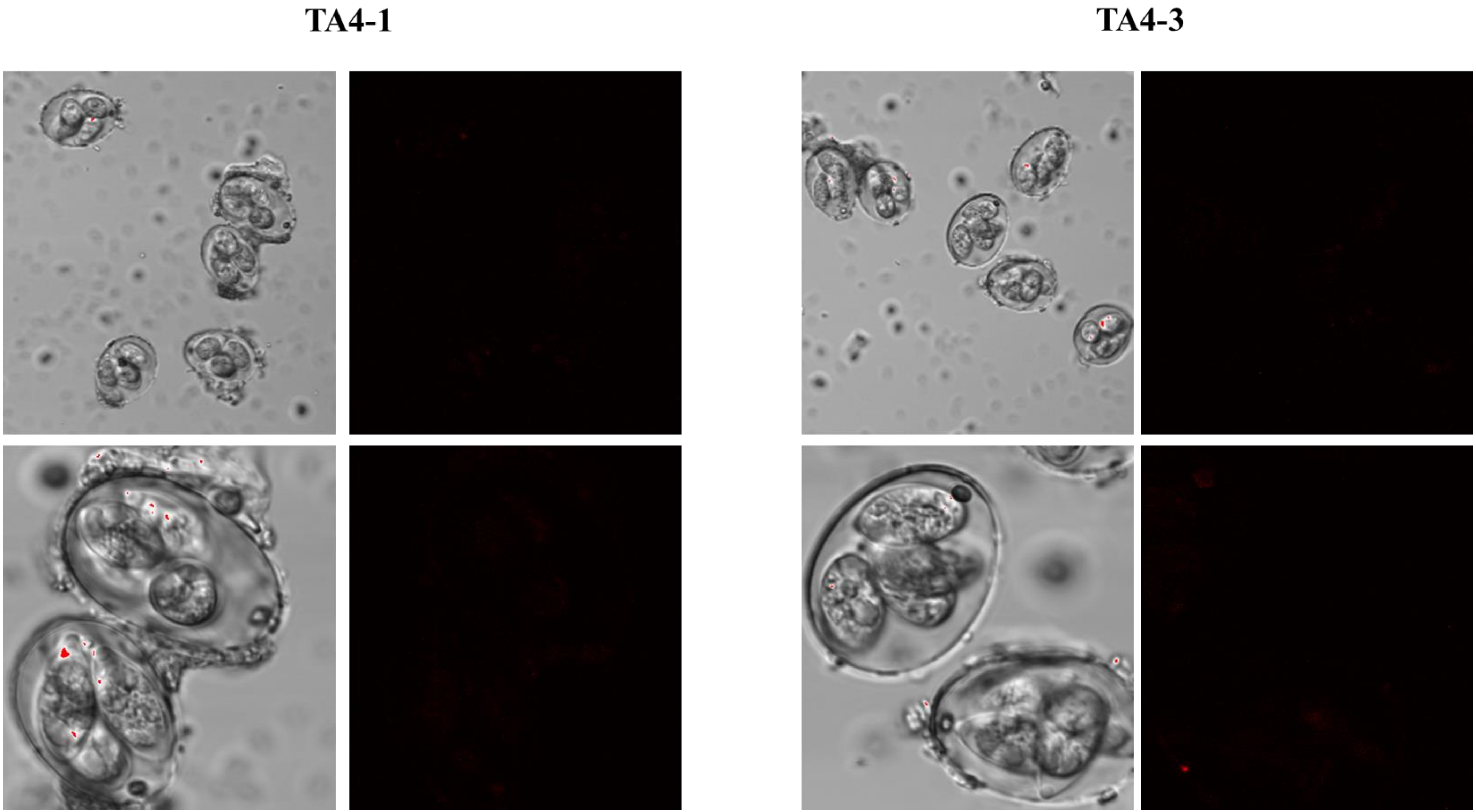
TA4-1
TA4-3

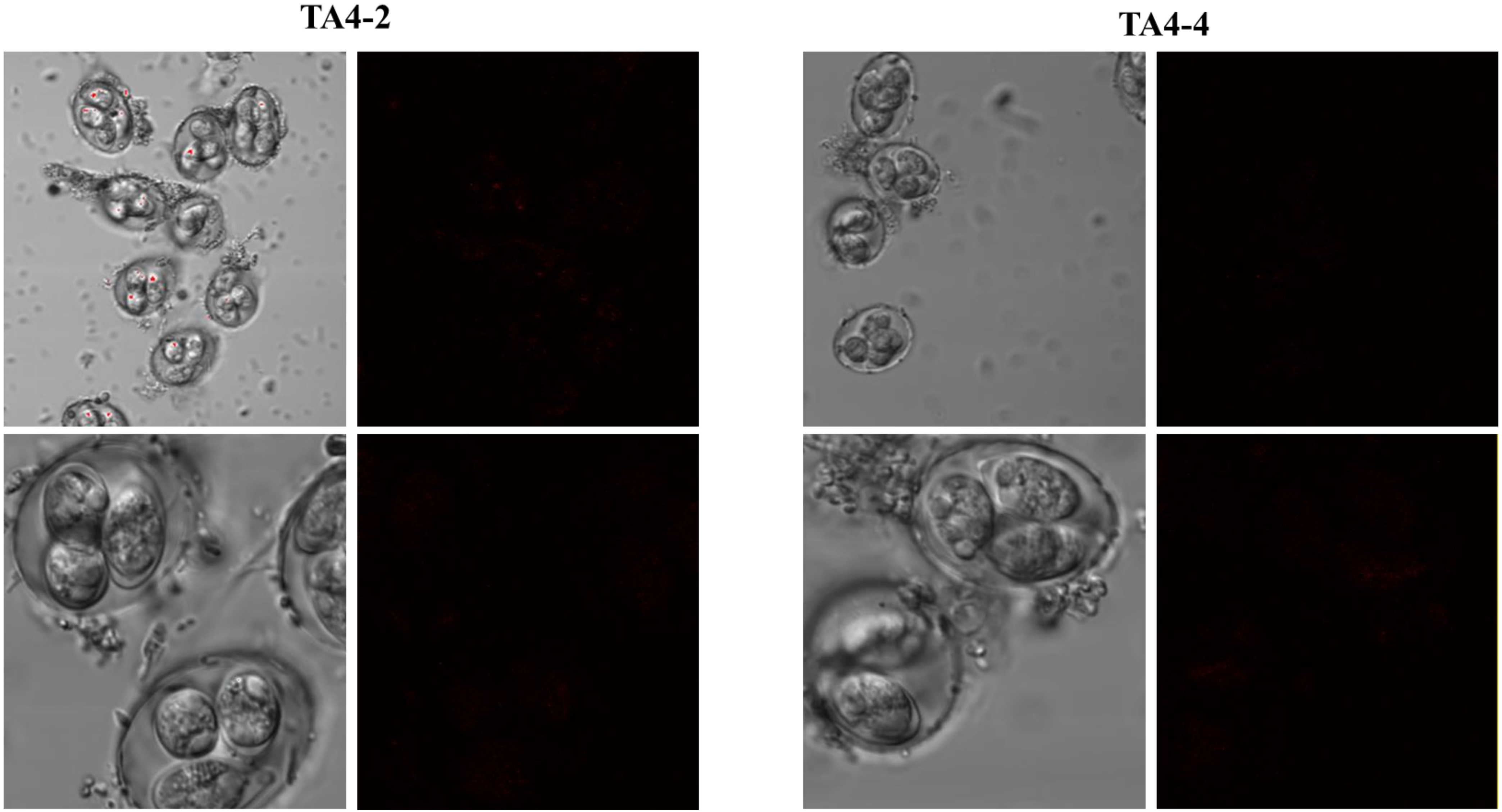
TA4-2
TA4-4

Whole Cyclo S16

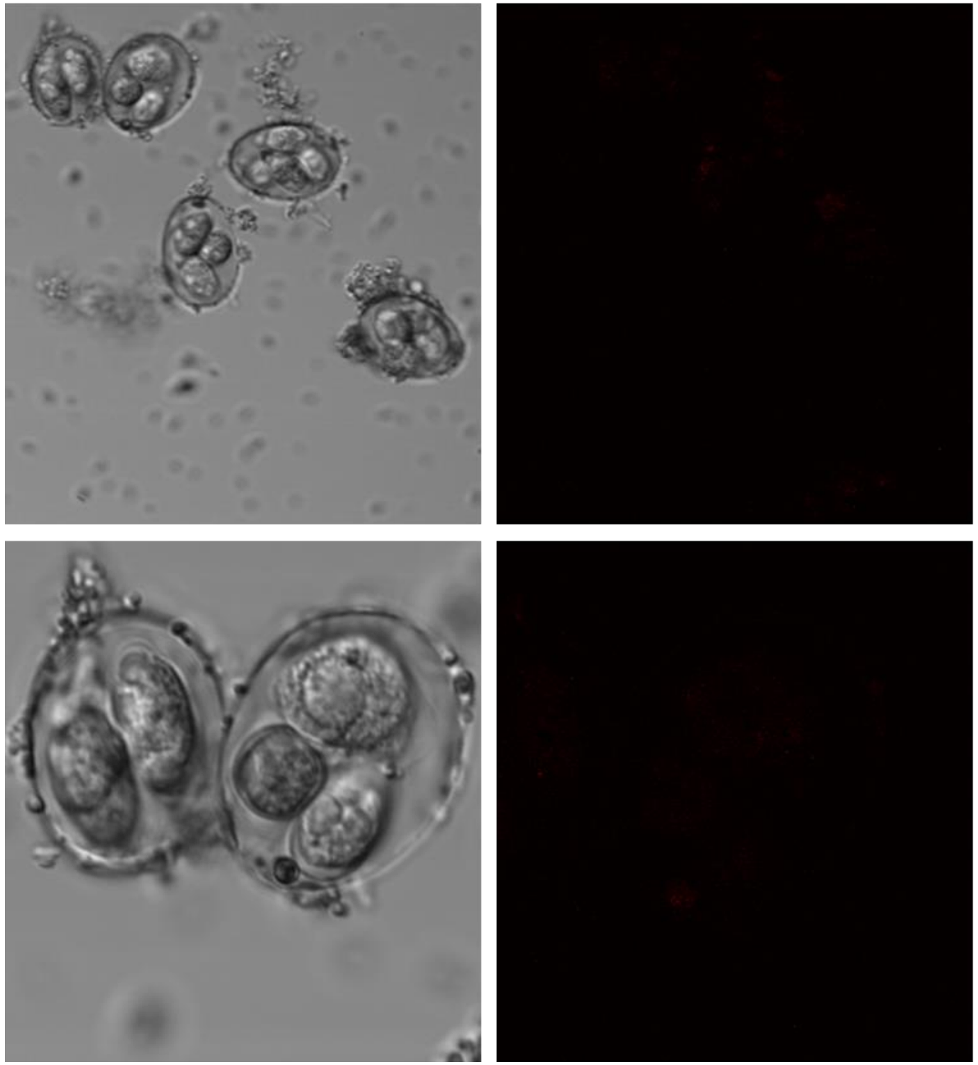

Whole Cyclo S3

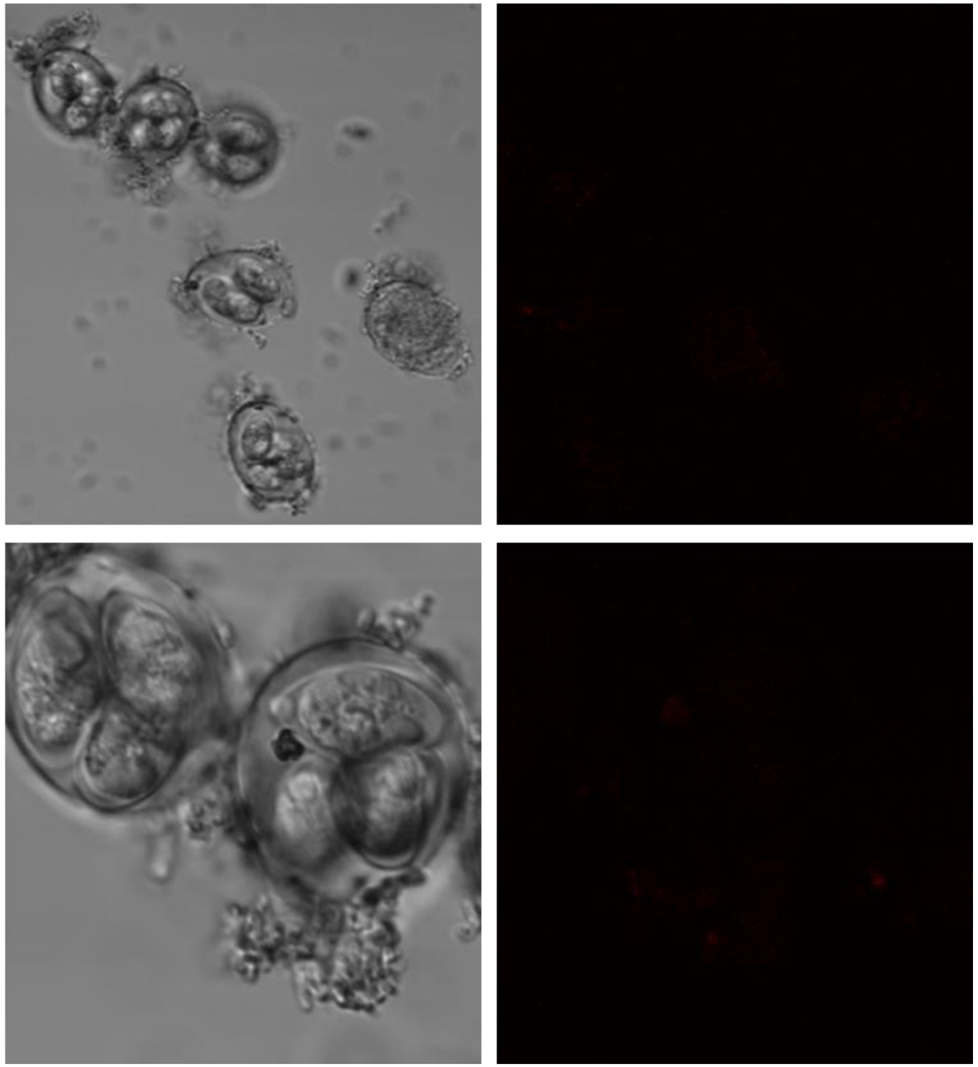

Assay Controls

No Aptamer
(-)ve Control

WP2-1 + Cyclospora
(+)ve Control

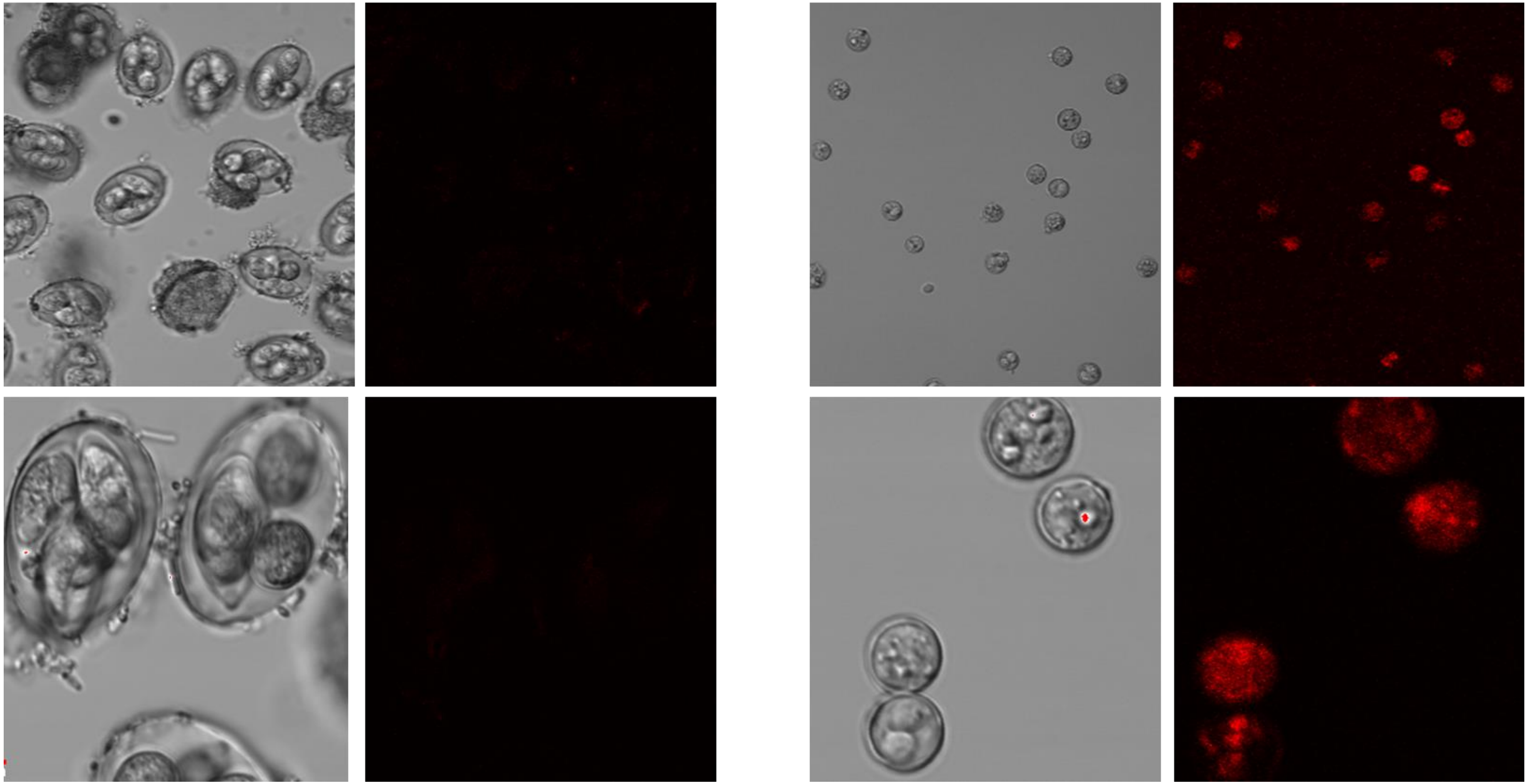

Conclusion: Eimaria maxima showed no positive signals with any of the aptamers.

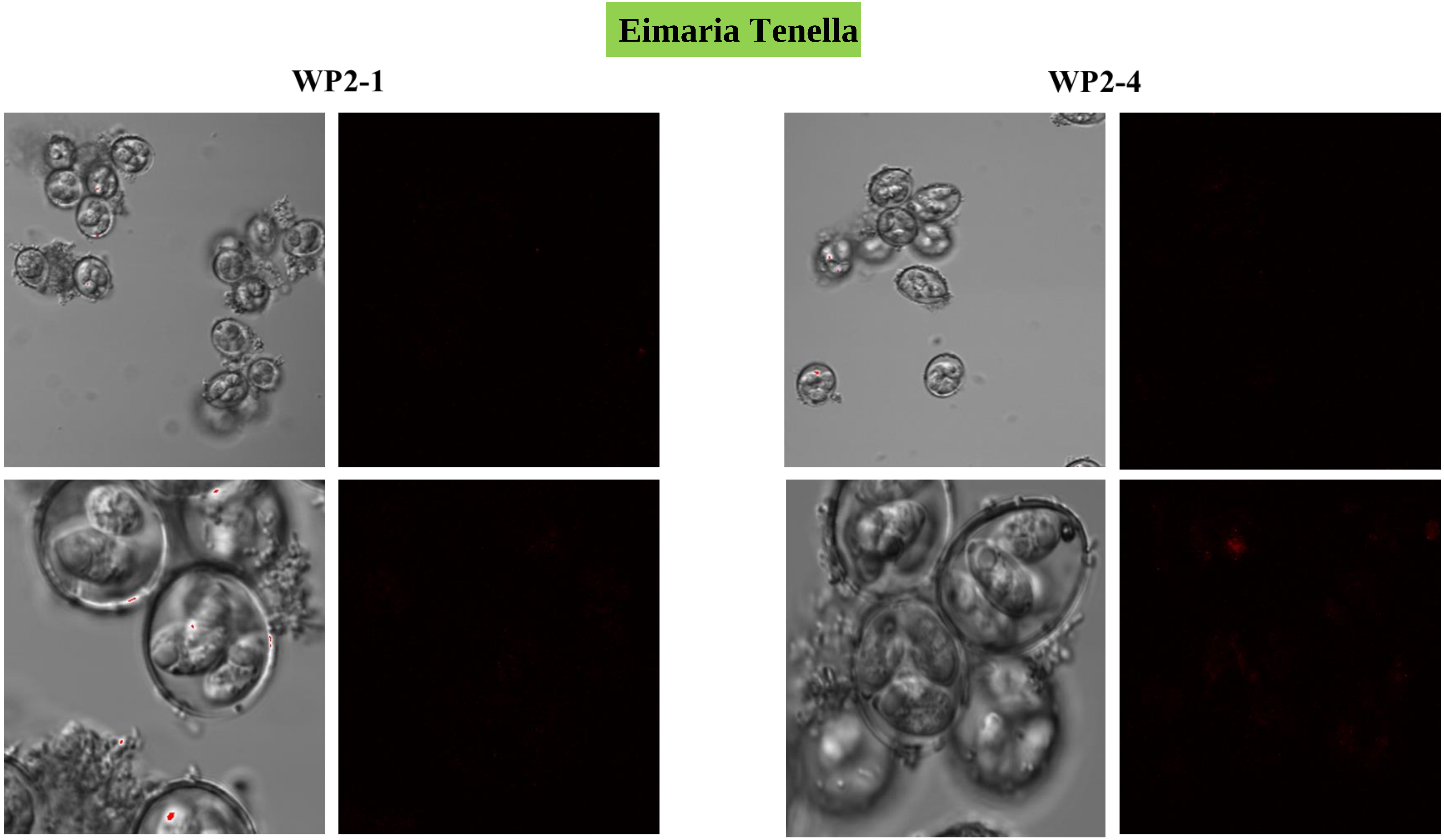
Eimaria Tenella
WP2-1
WP2-4

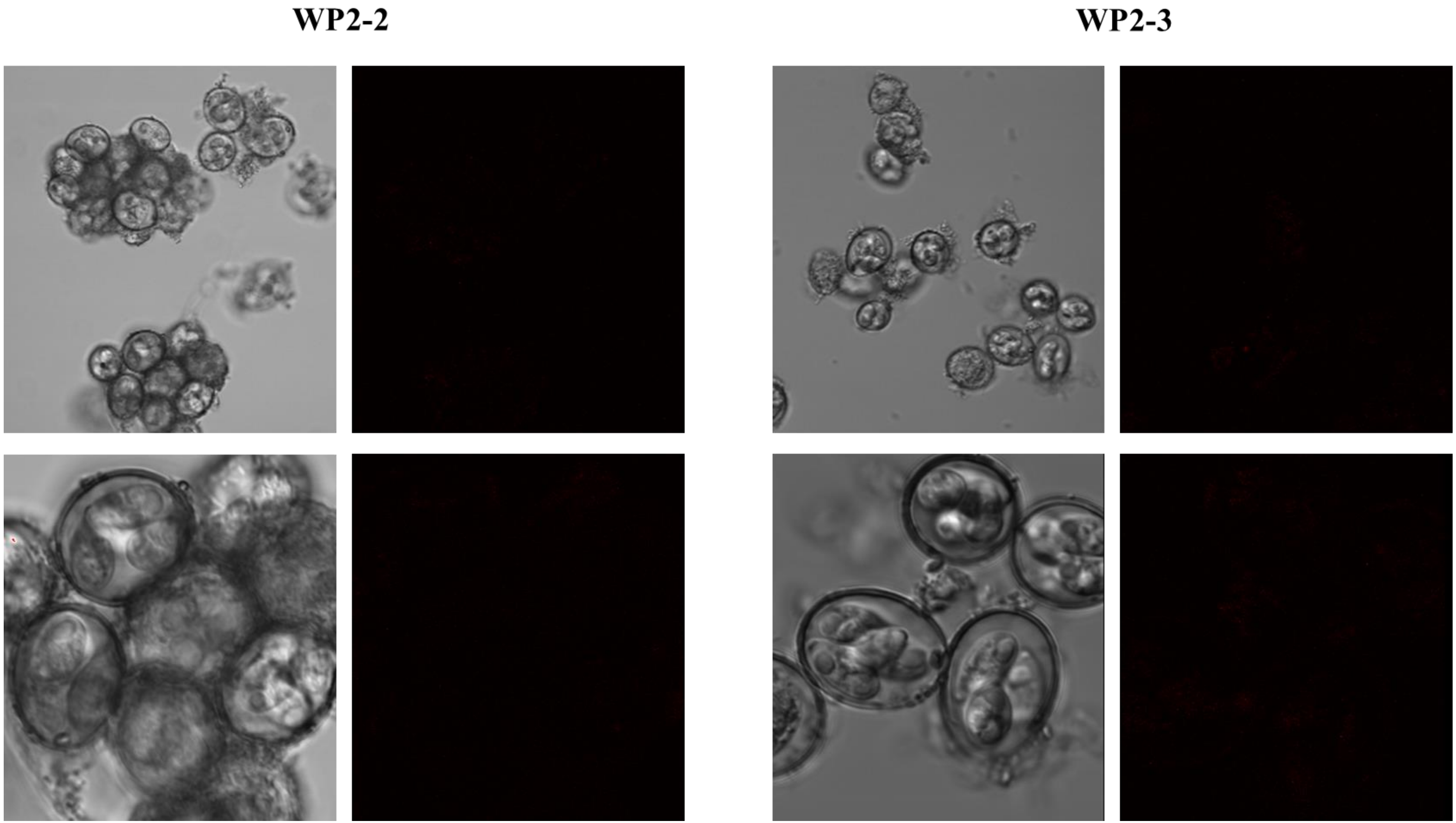
WP2-2
WP2-3

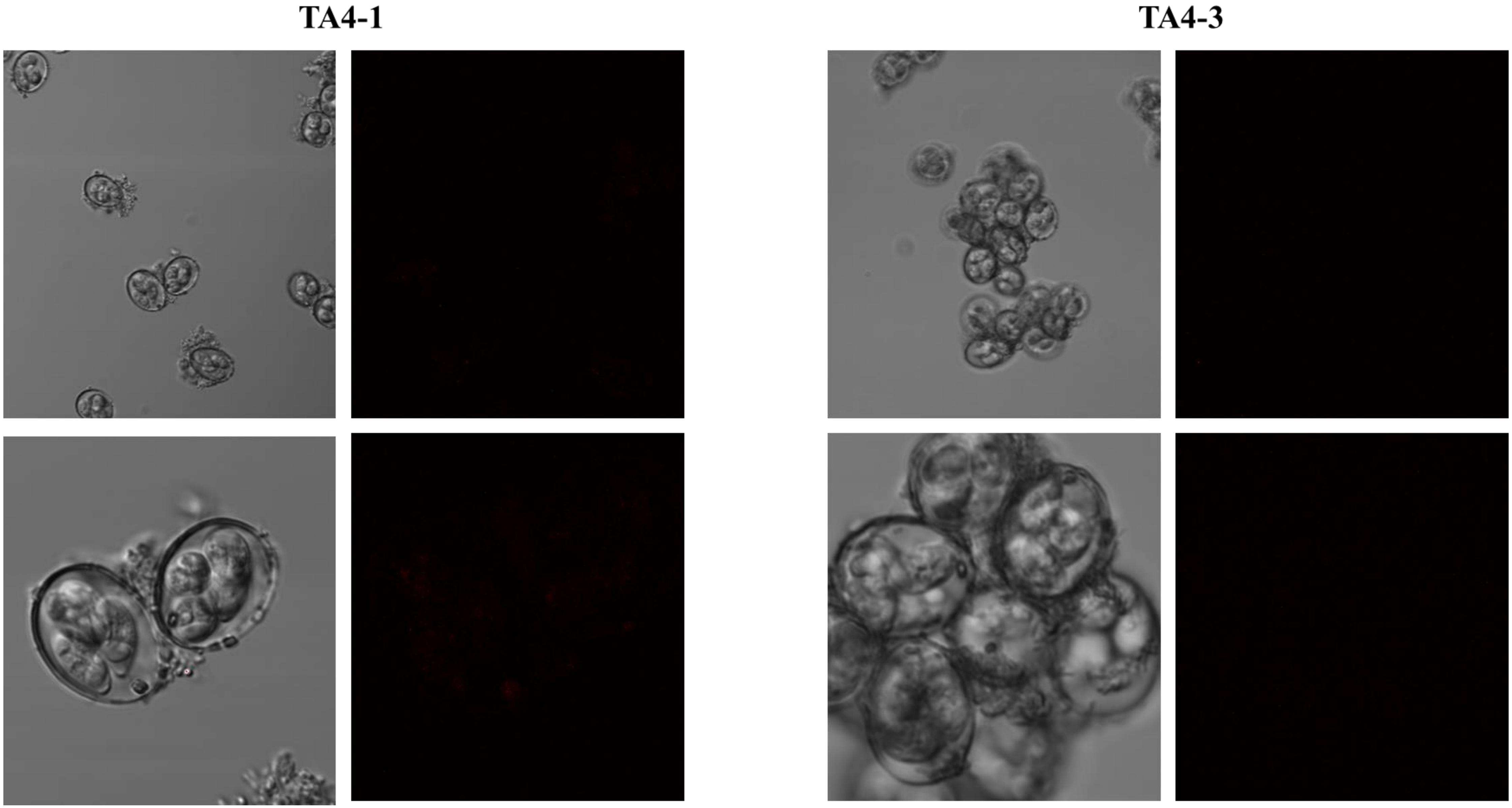
TA4-1
TA4-3

TA4-2

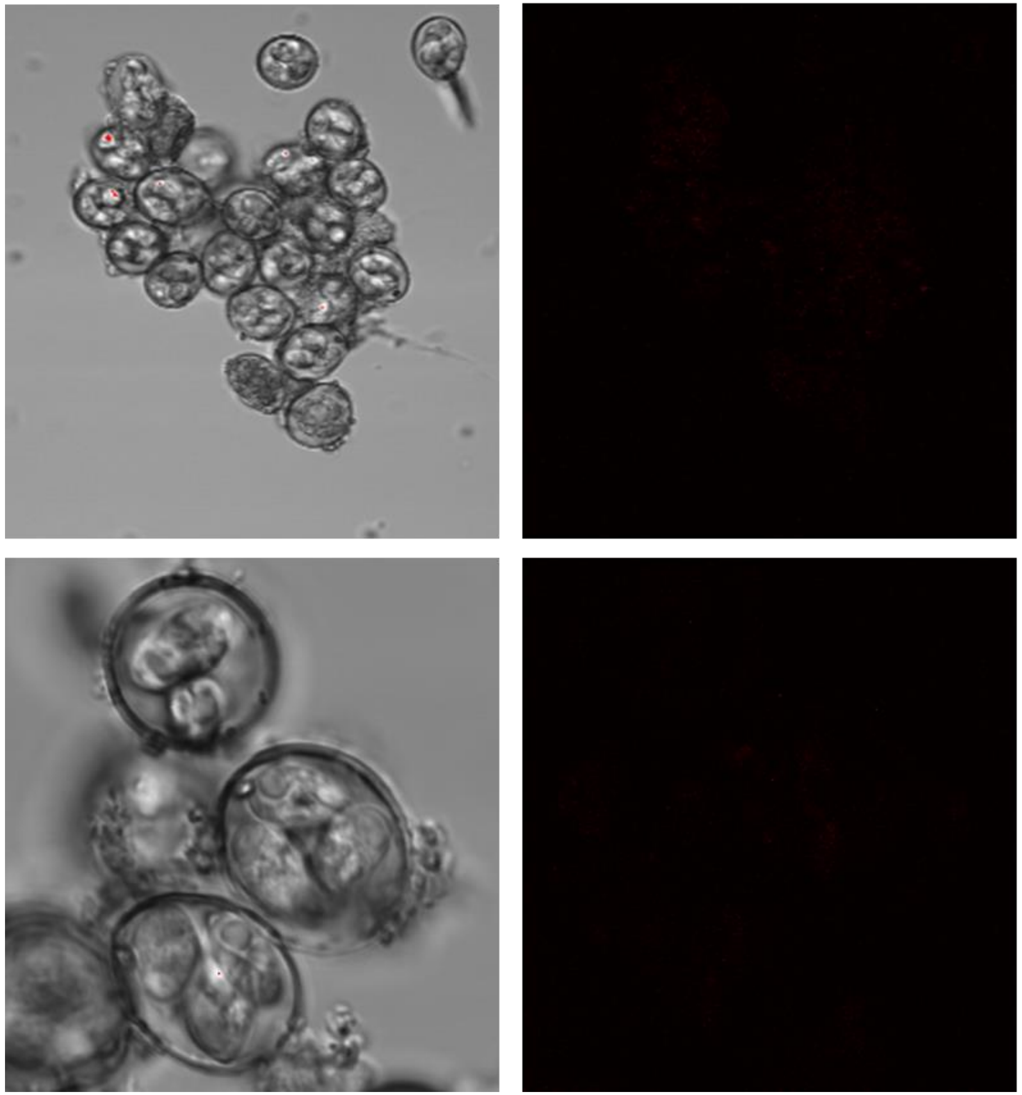

TA4-4

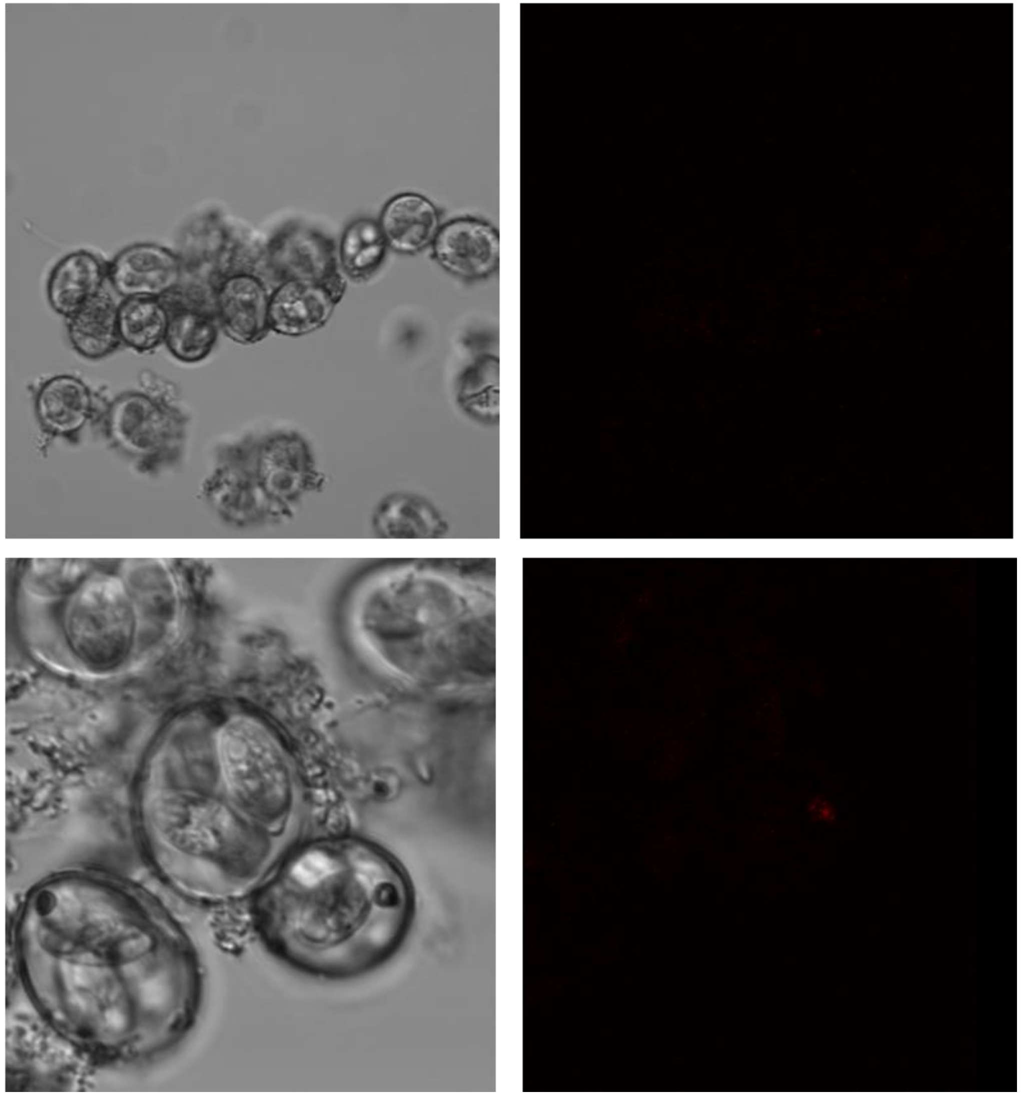

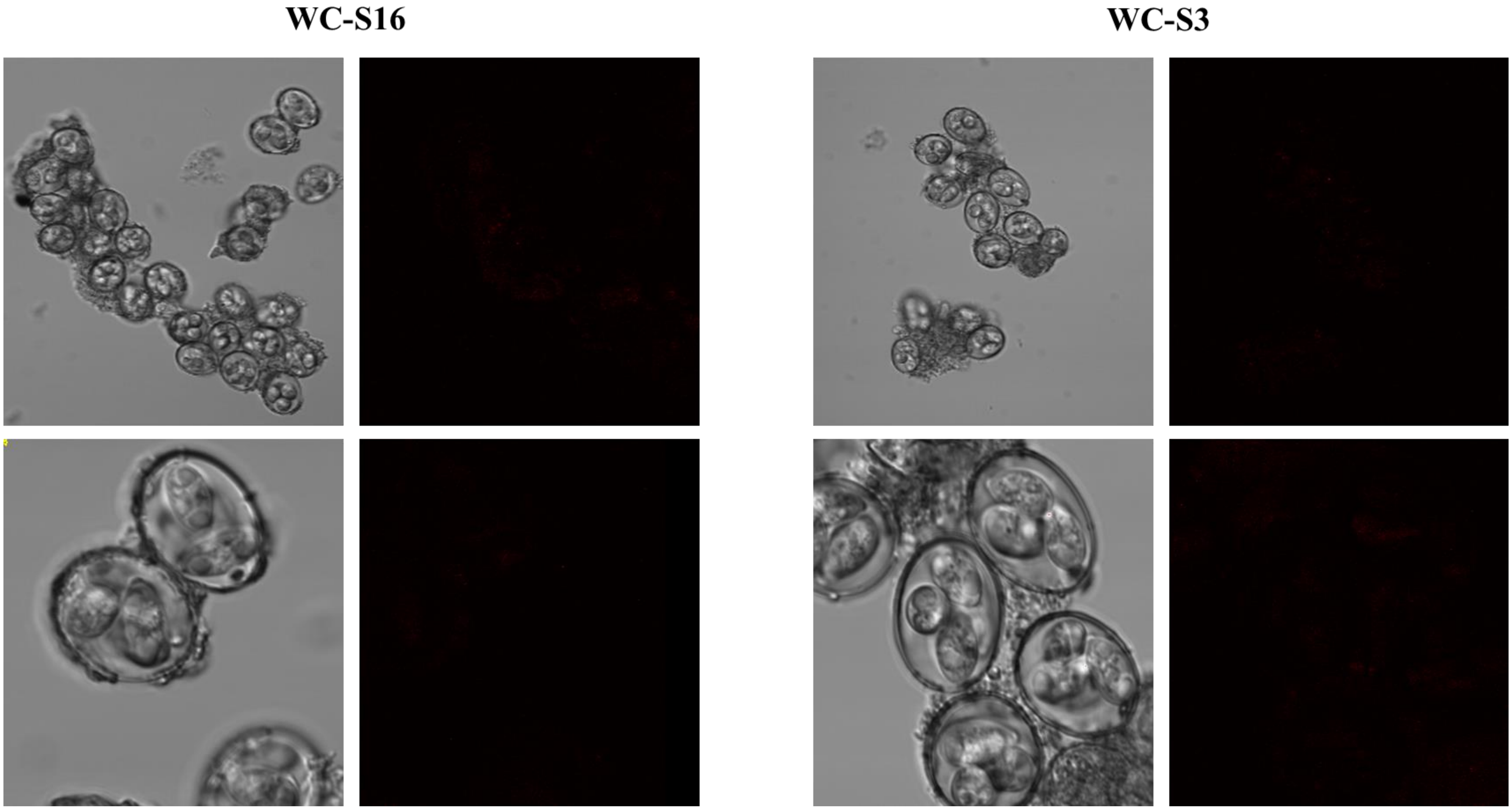
WC-S16
WC-S3

Assay Controls

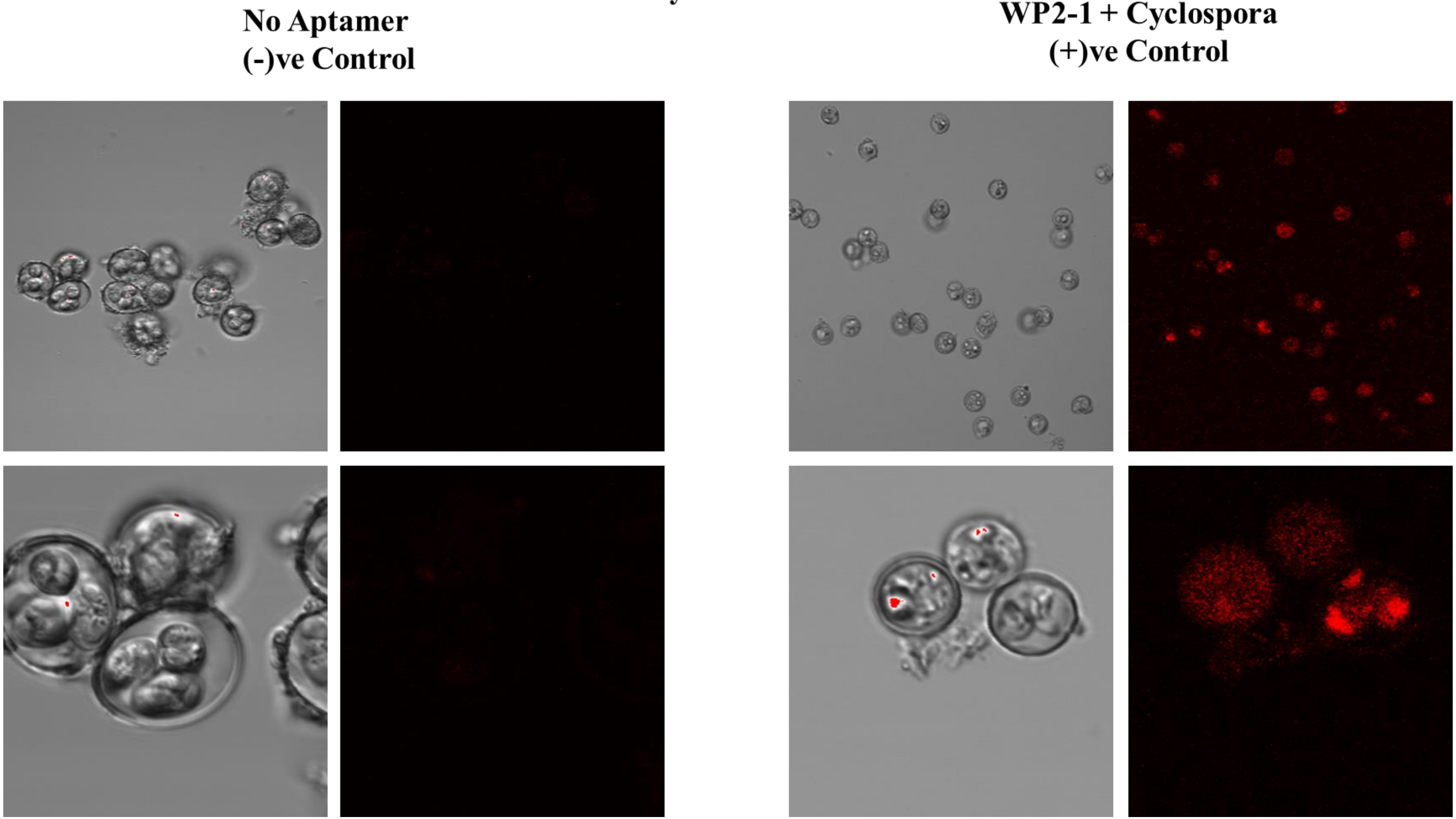

Conclusion: Eimaria tenella is negative with all aptamers. There is no positive signal.

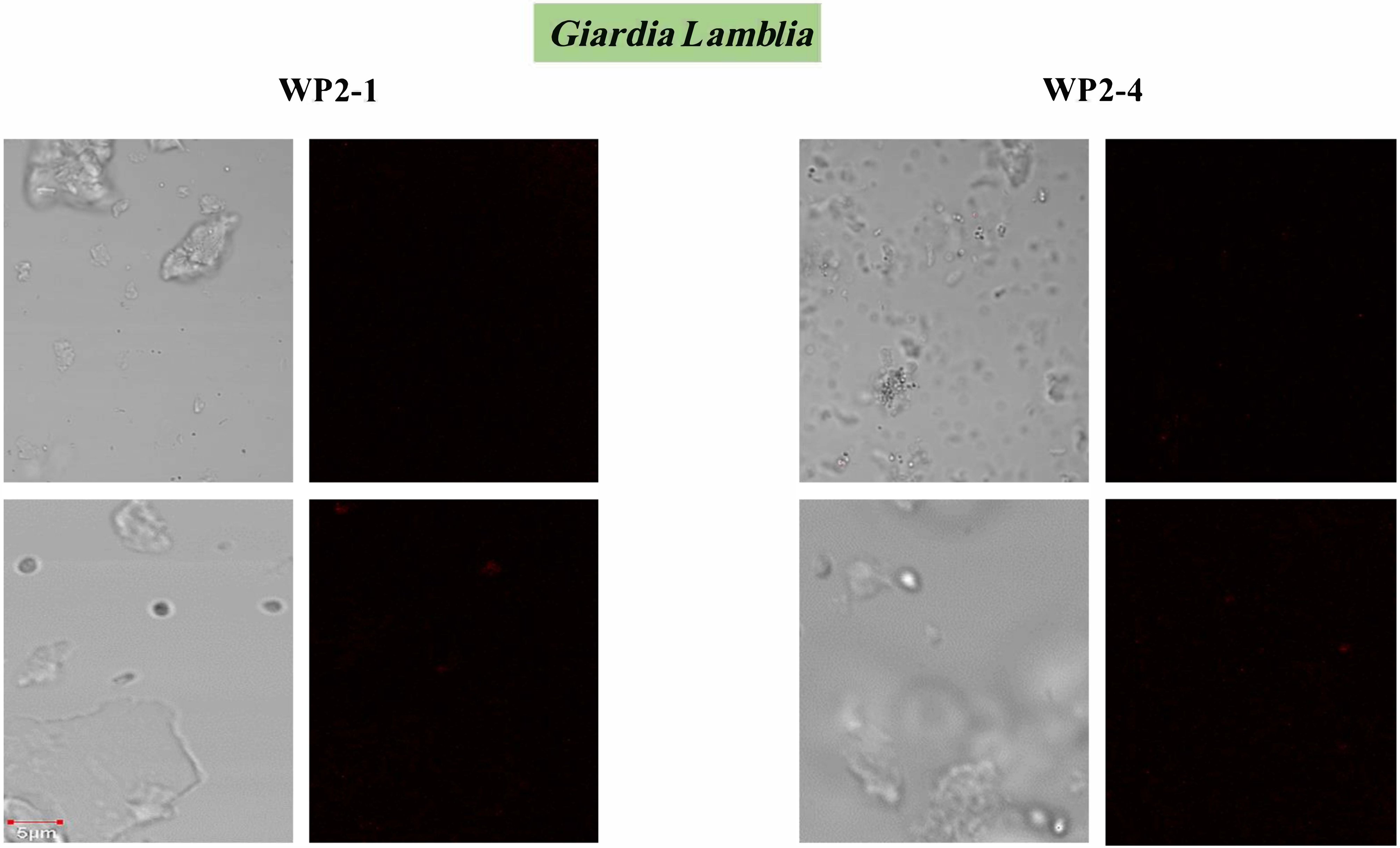
Giardia Lamblia
WP2-1
WP2-4
5μm

WP2-2

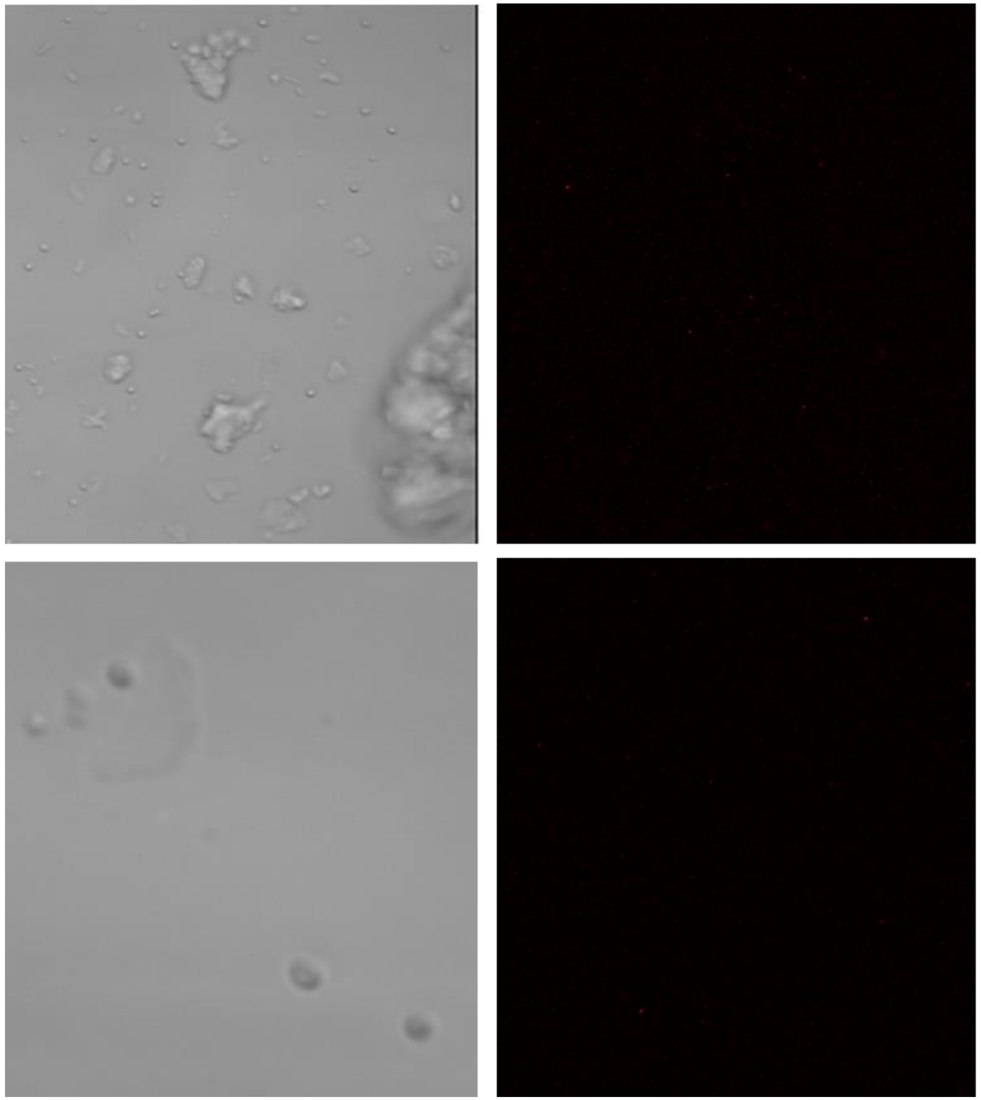

WP2-3

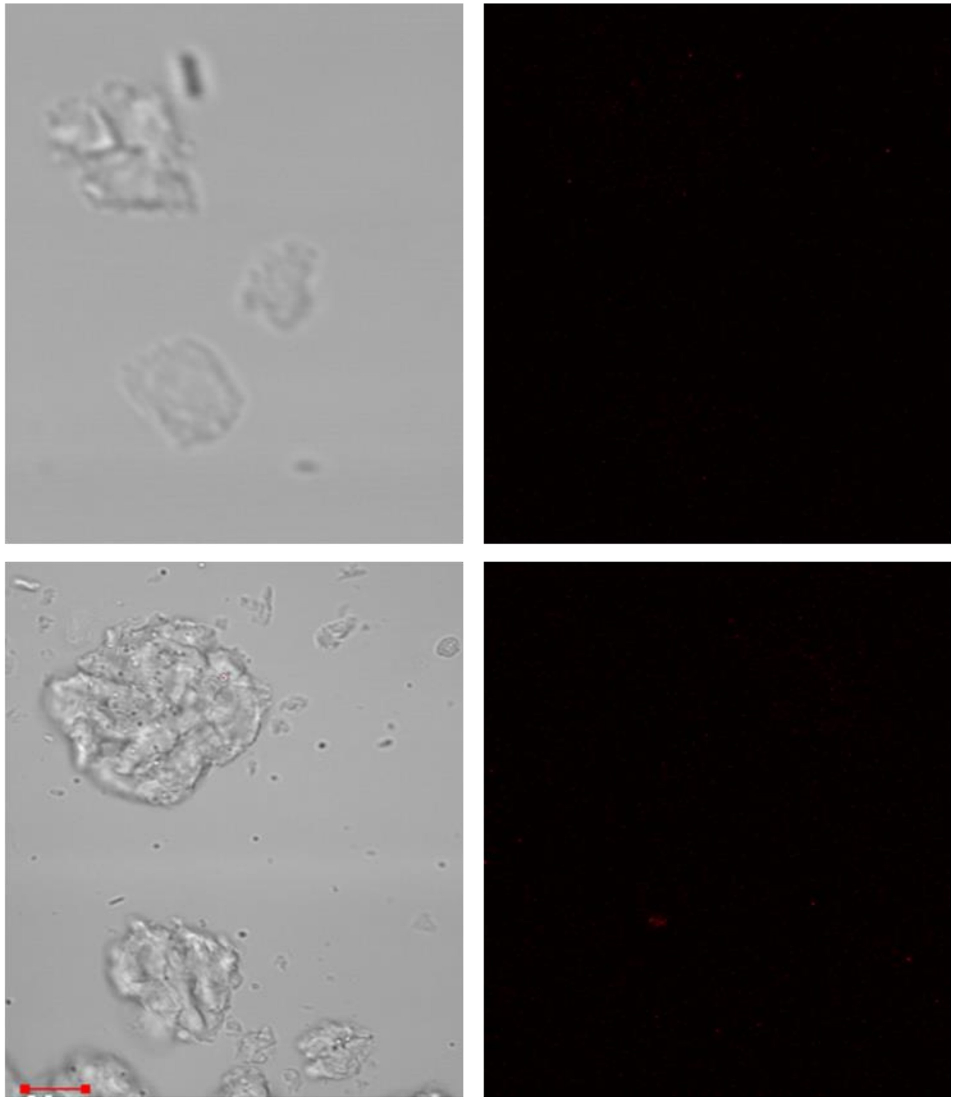

TA4-1

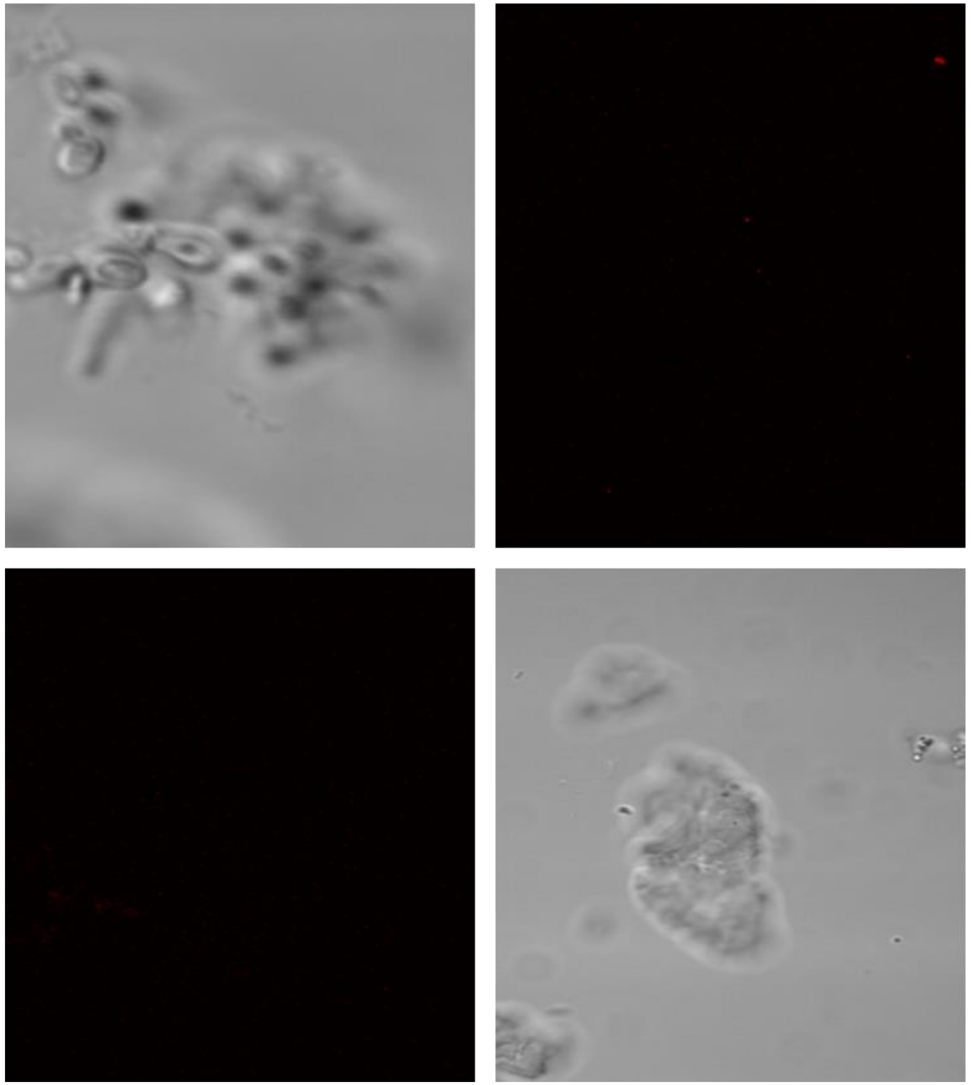

TA4-3

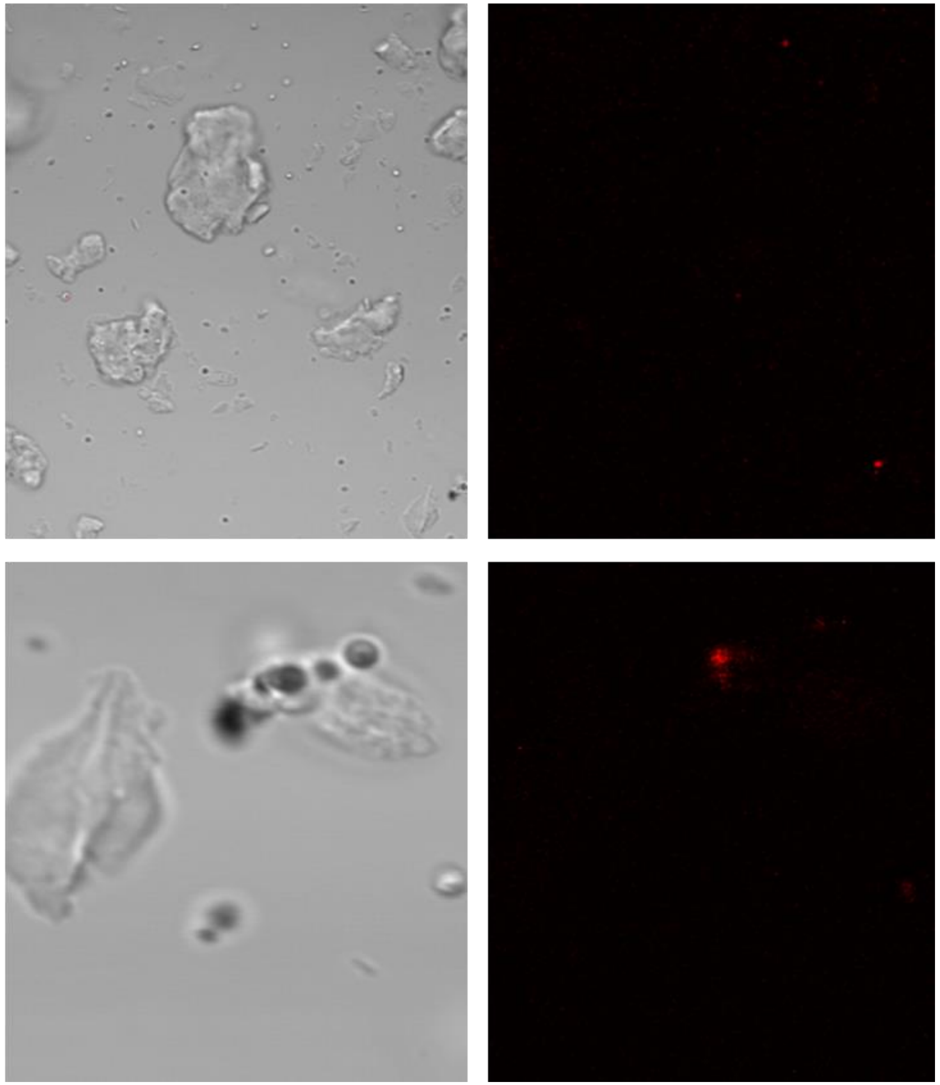

TA4-2

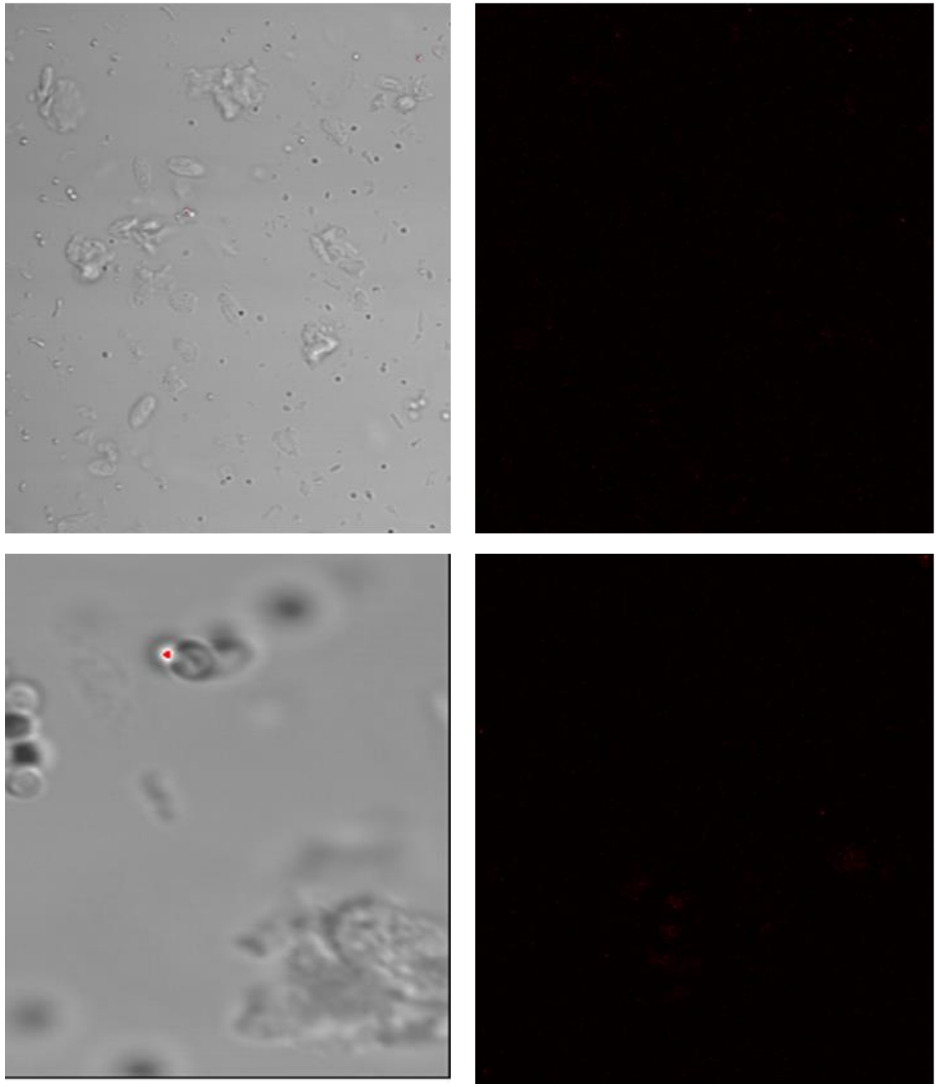

TA4-4

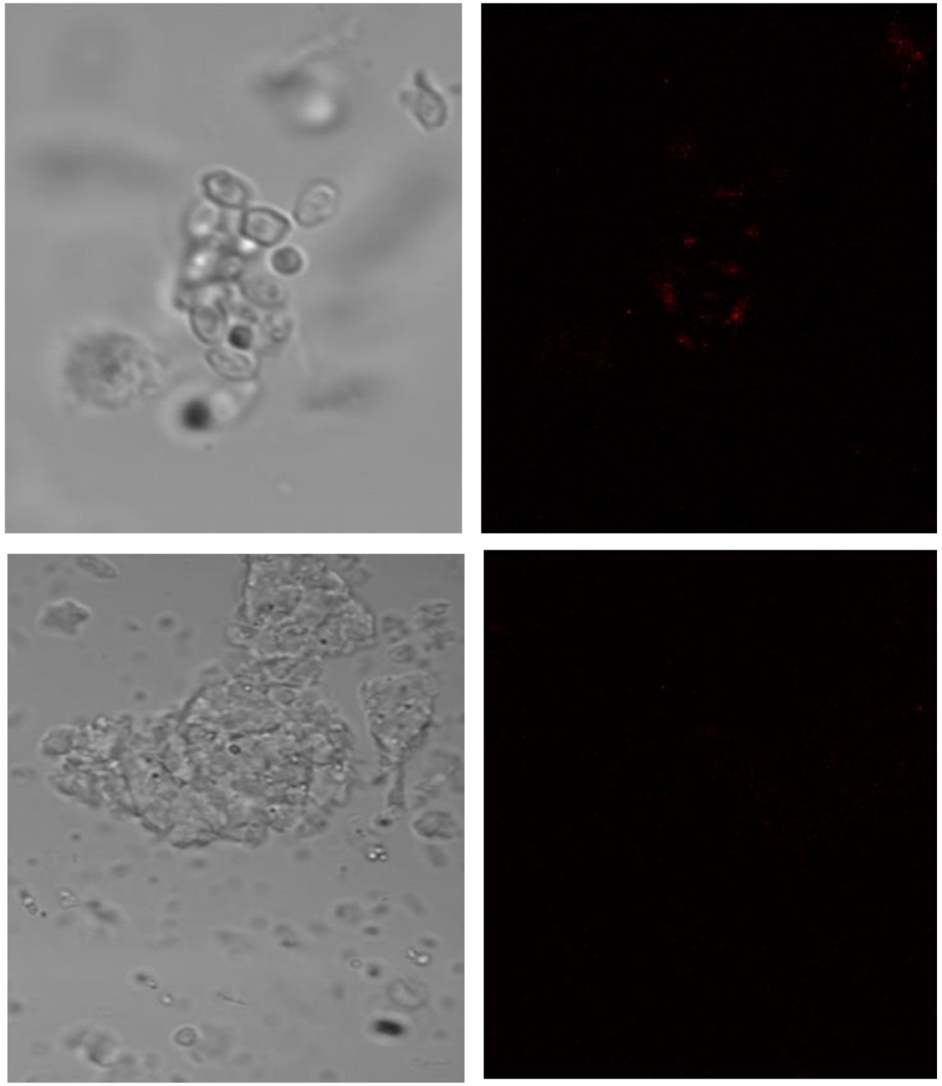

Whole Cyclo S16

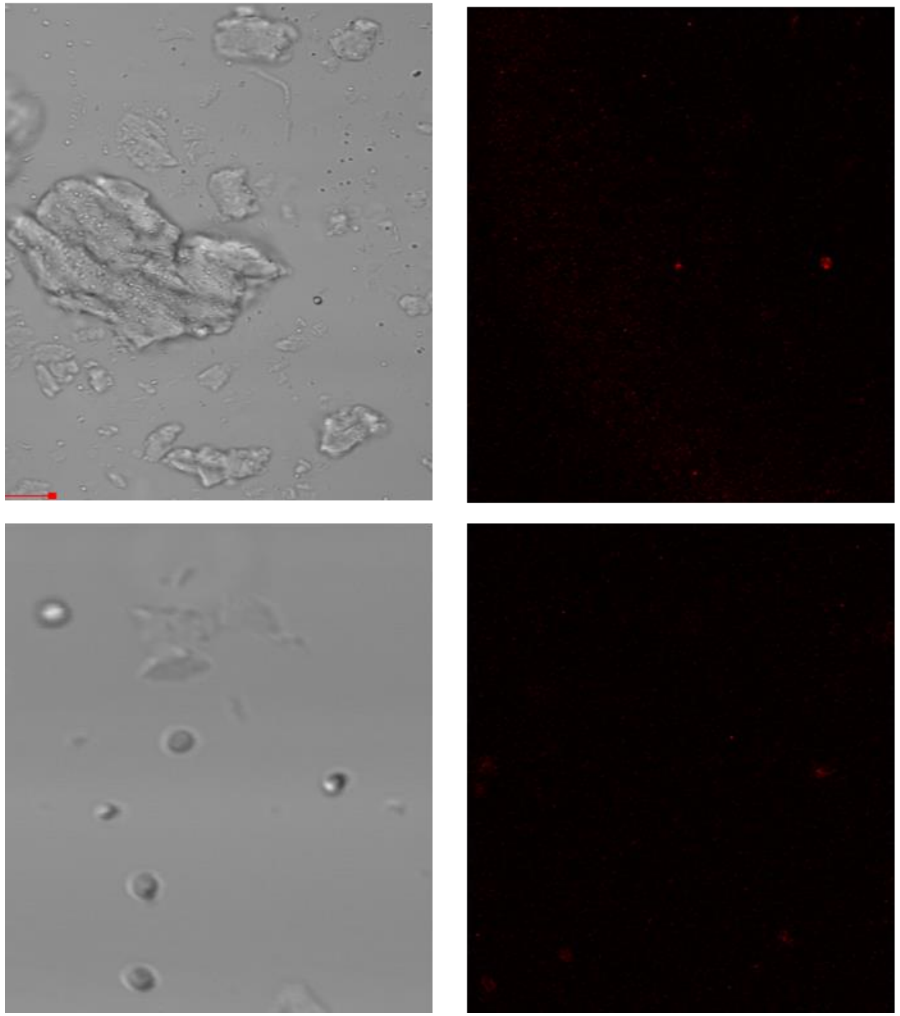

Whole Cyclo S3

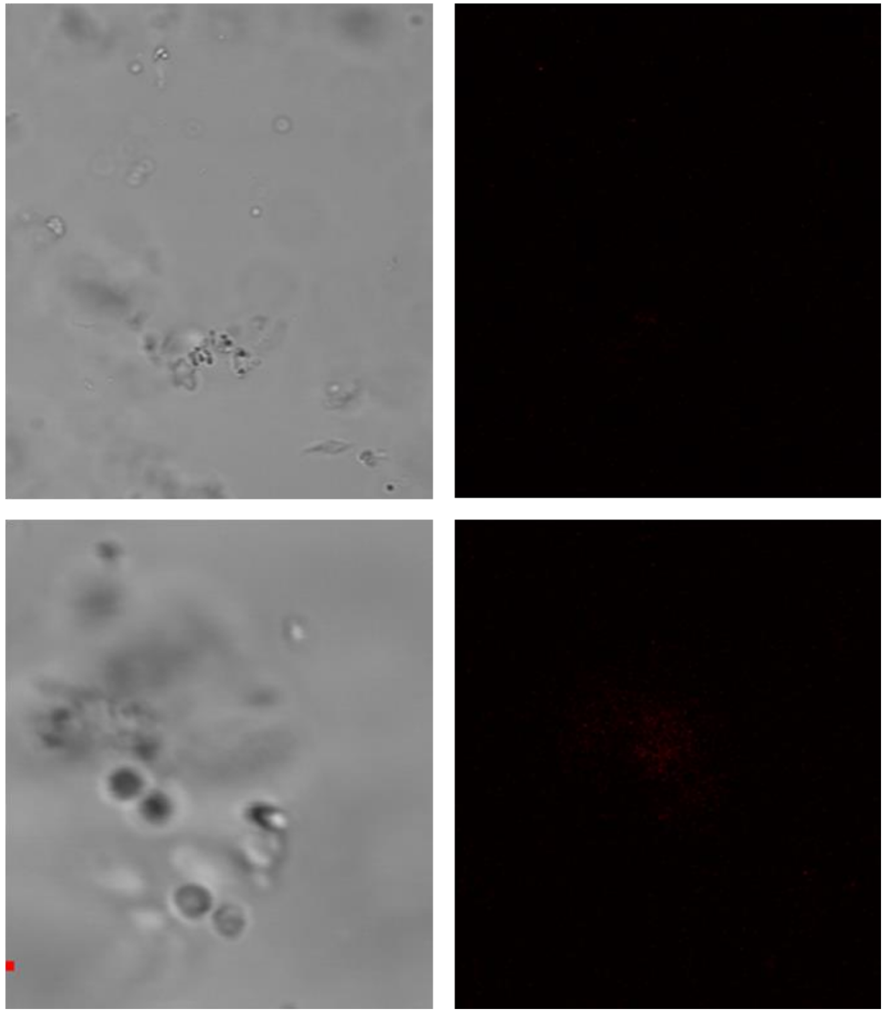

Assay Controls

No Aptamer (-)ve Control | **WP2-1 + Cyclospora (+)ve Control**

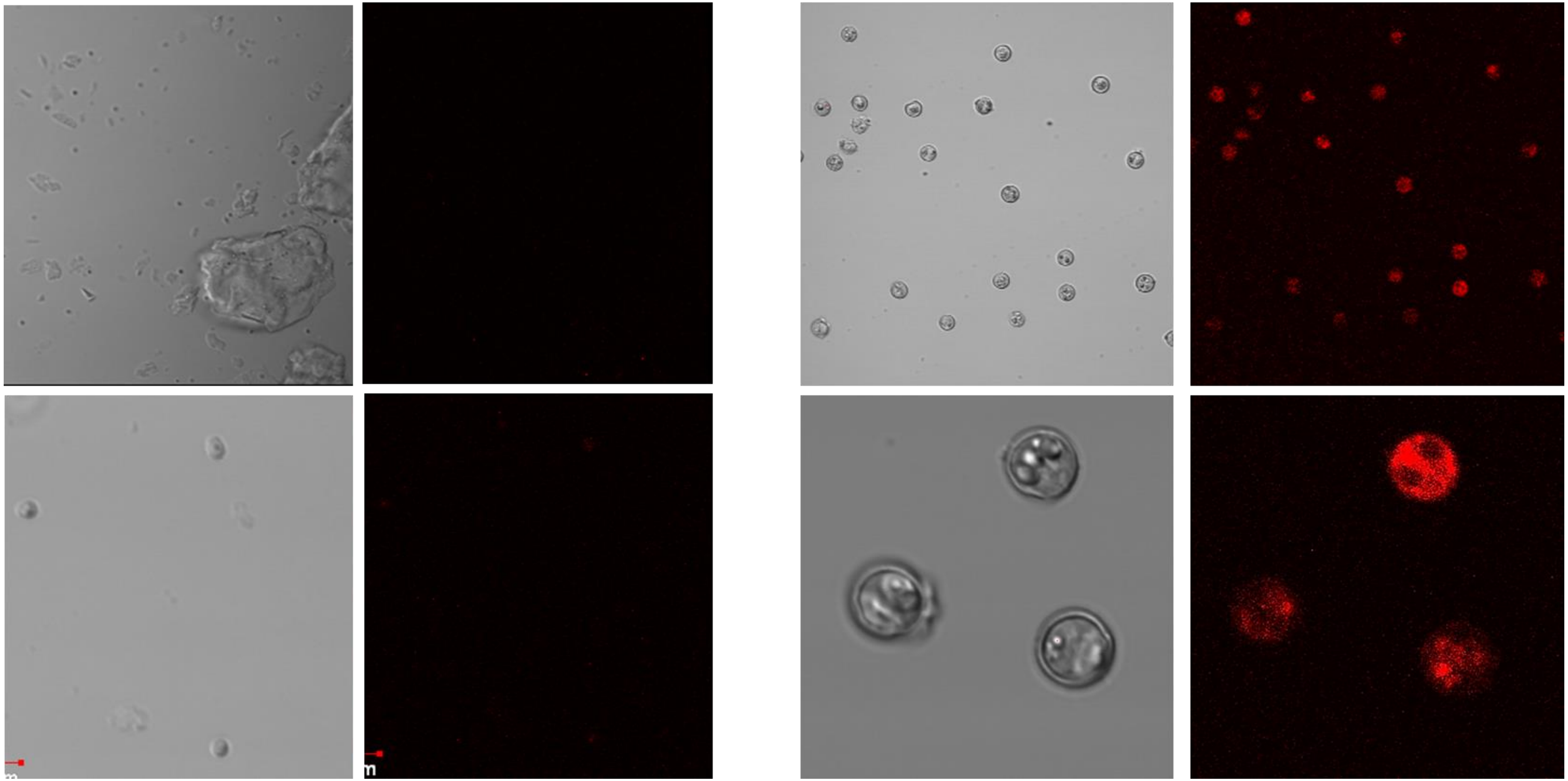

Conclusion: Giardia lamblia showed no positive signals with any of the aptamers.

Naeglaria Fowleri

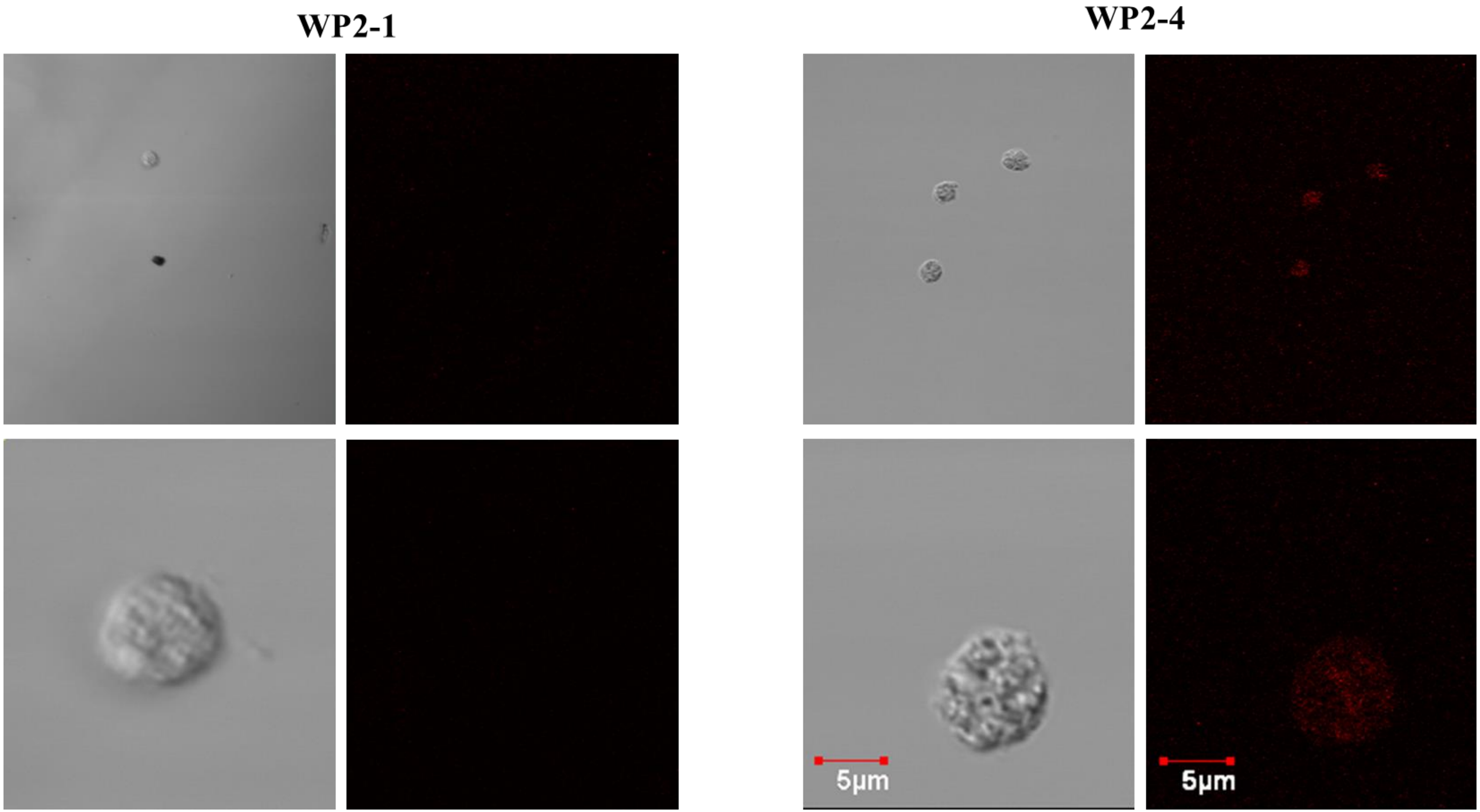

WP2-2

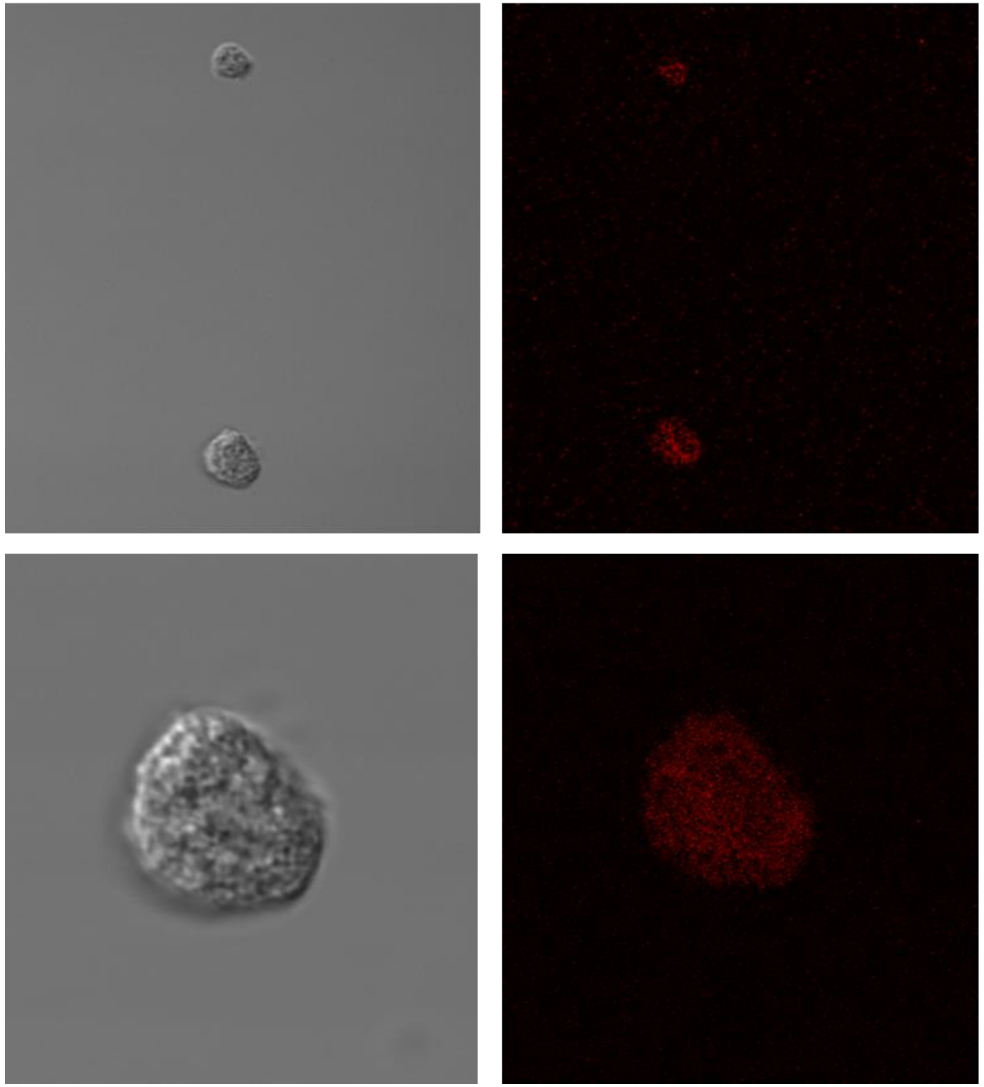

WP2-3

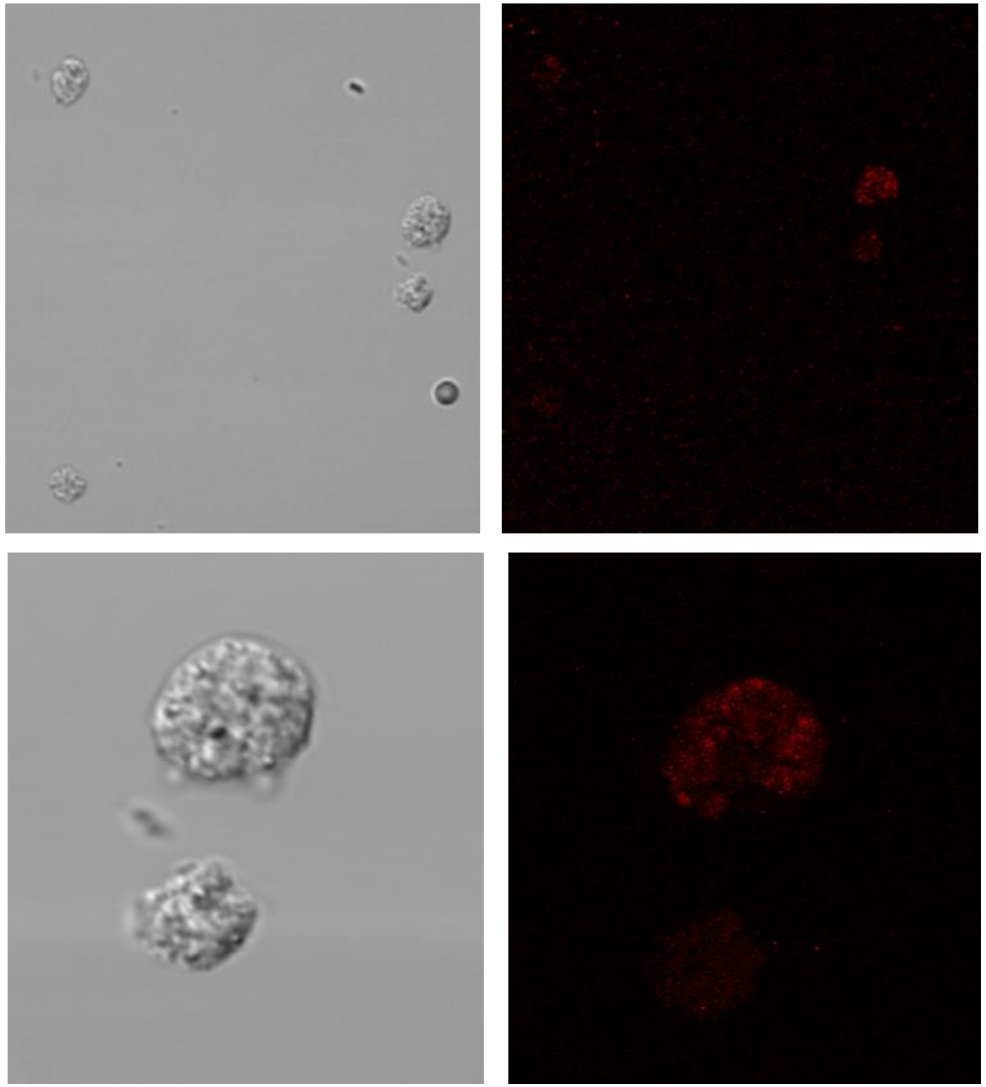

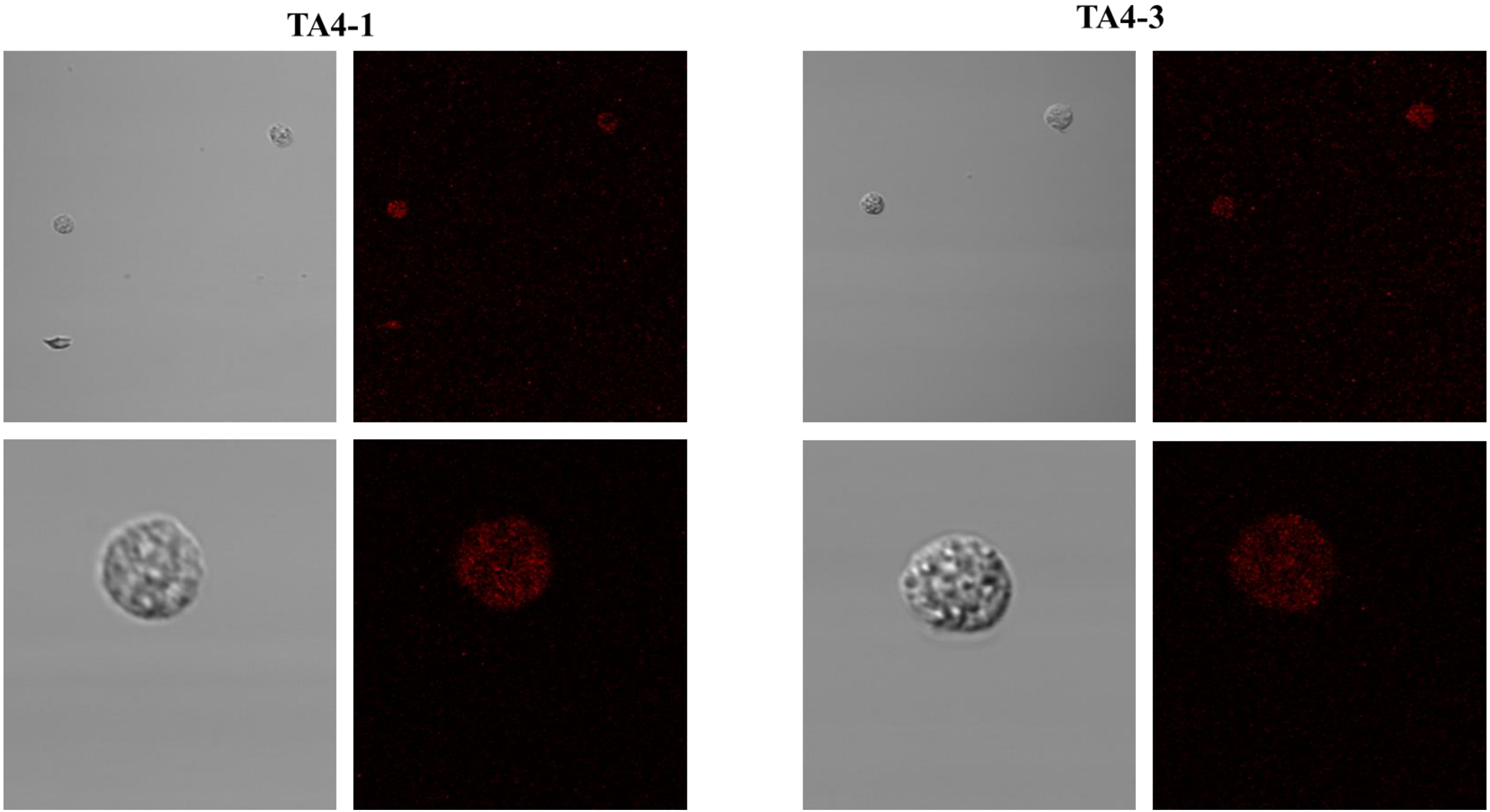
TA4-1
TA4-3

TA4-2

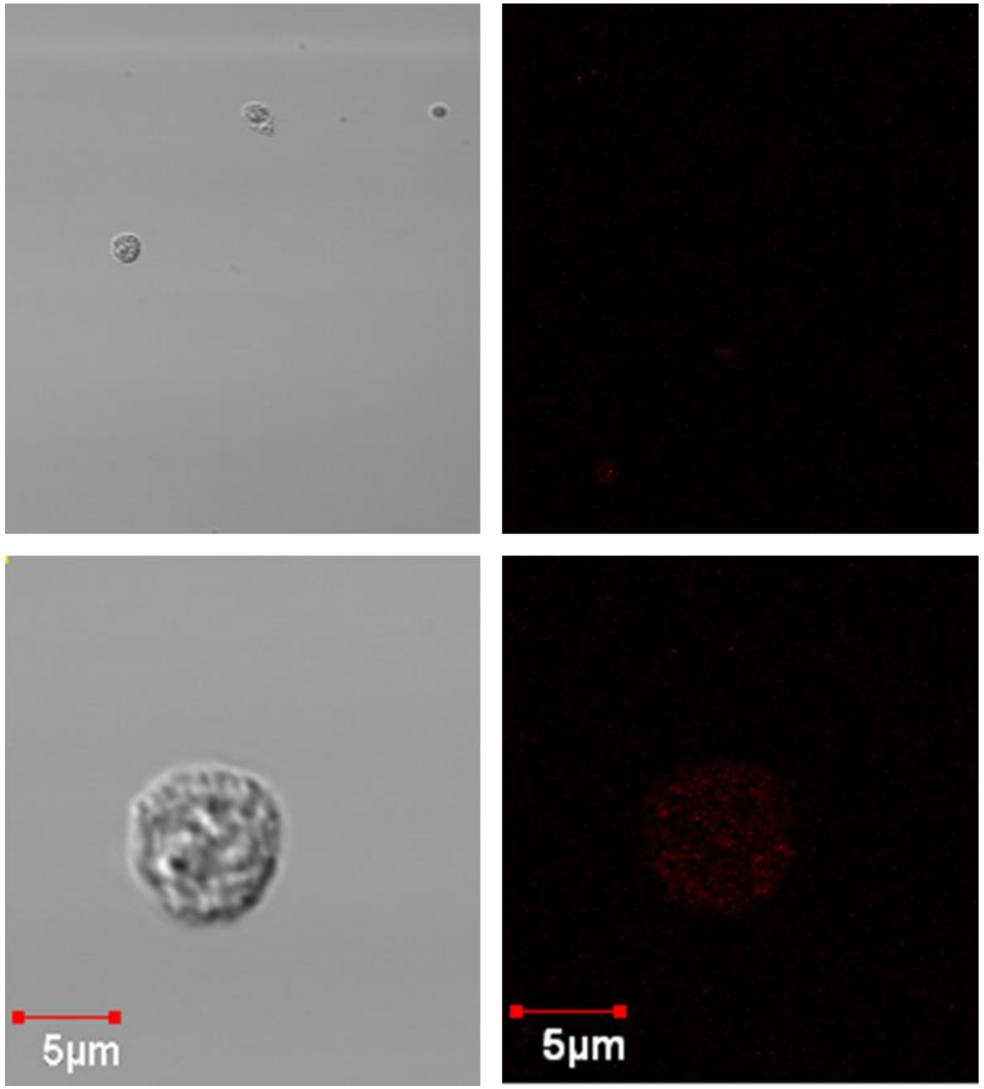

TA4-2

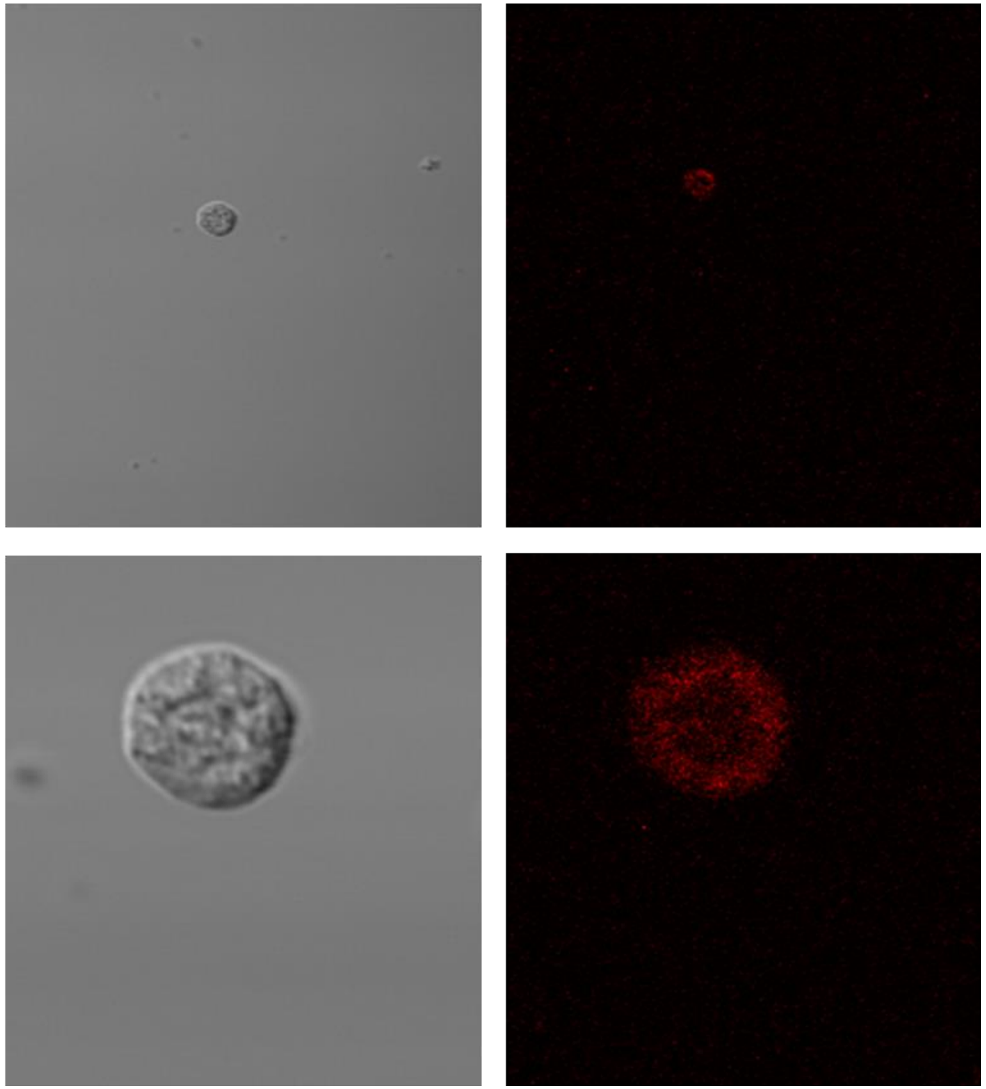

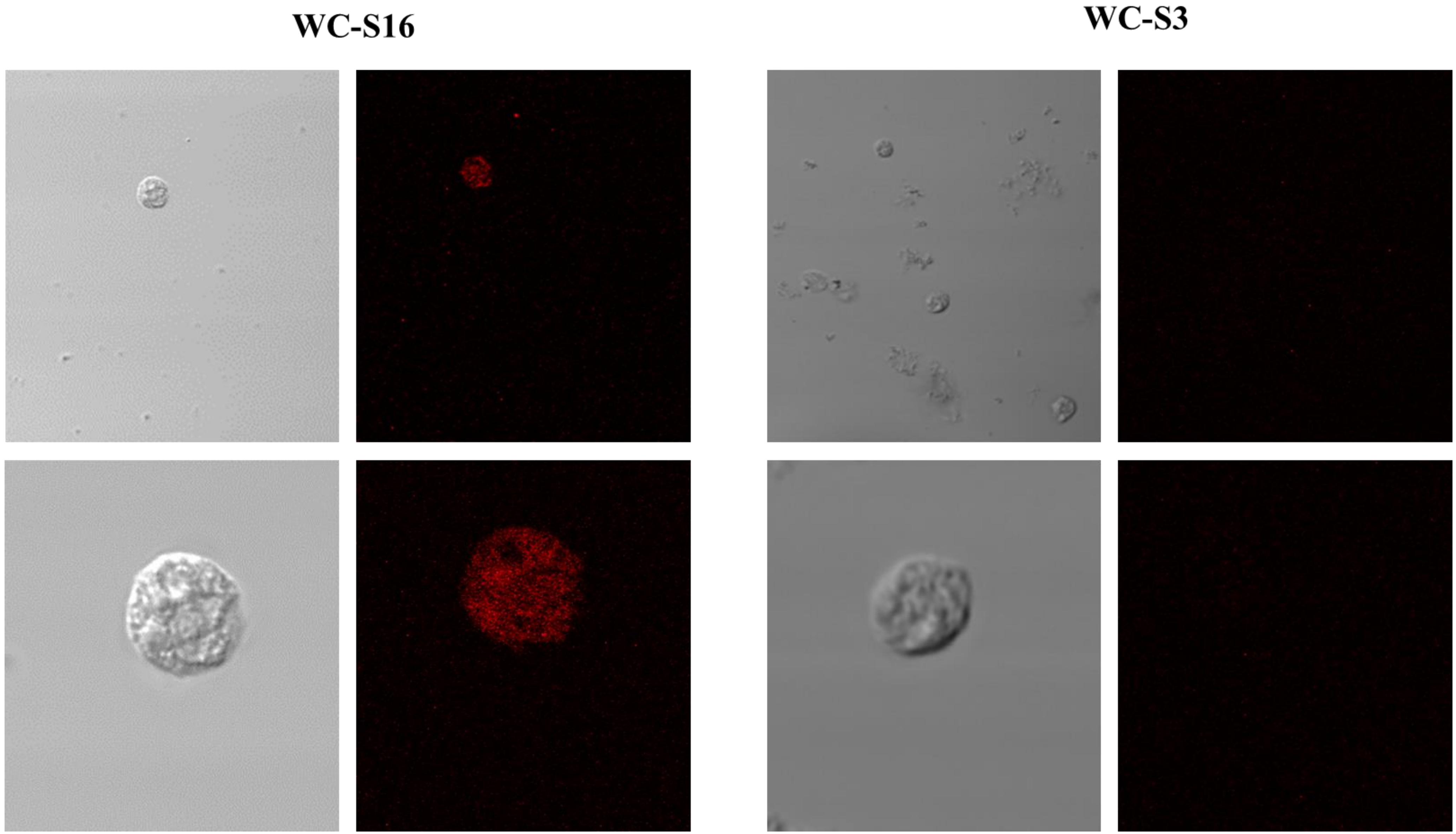
WC-S16
WC-S3

Assay Controls

No Aptamer
(-)ve Control

WP2-1 + Cyclospora
(+)ve Control

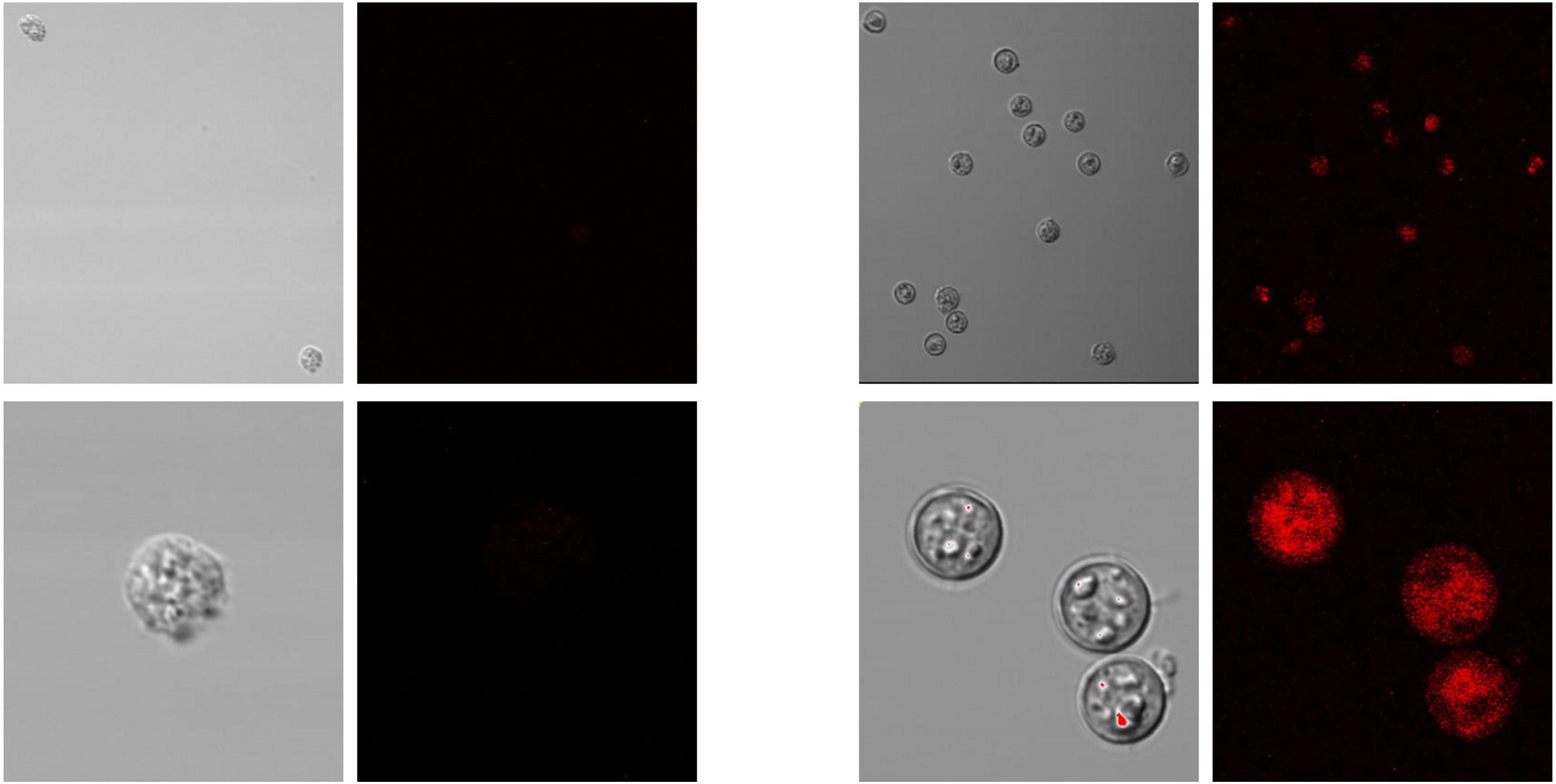

Conclusion: Except for WP2-2 and WC-S3, all aptamer showed a slight positive signals for Naeglaria fowleri, but signals were not nearly as strong as with Cyclospora.

Toxoplasma Gondii

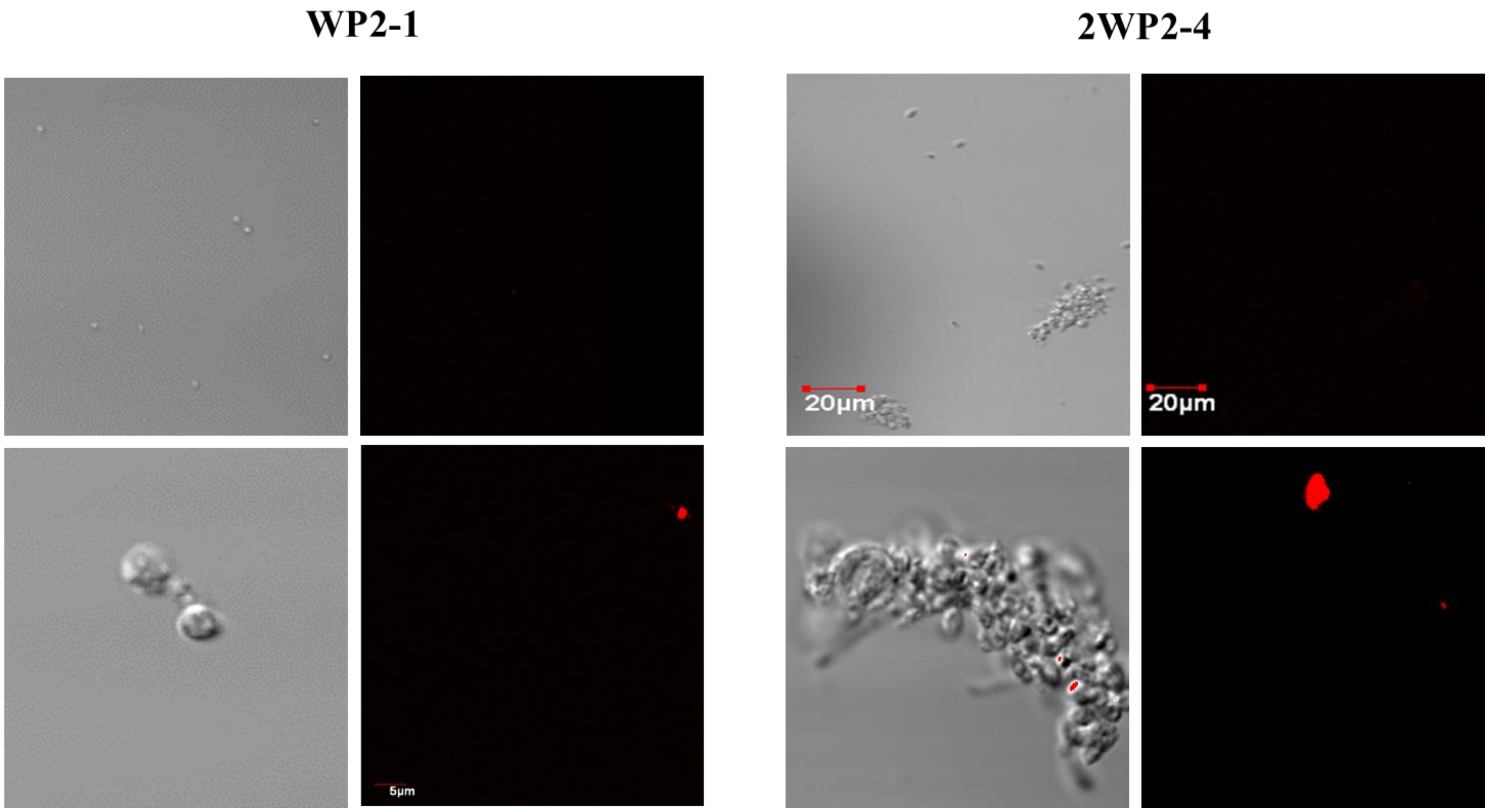

WP2-2

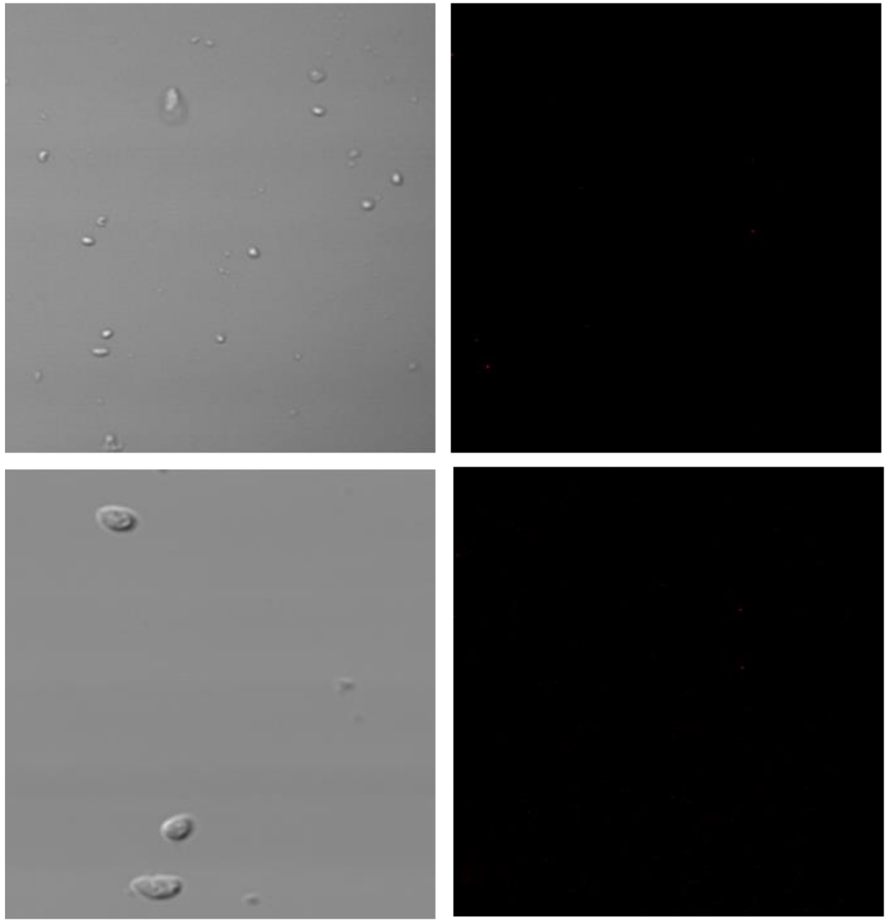

WP2-3

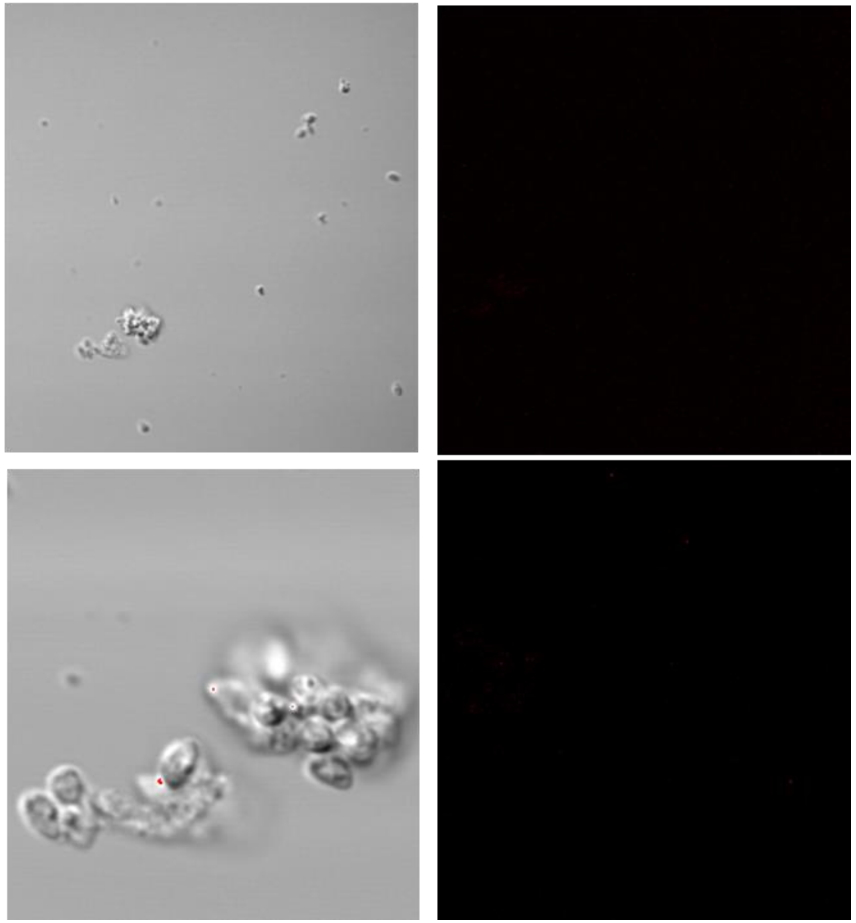

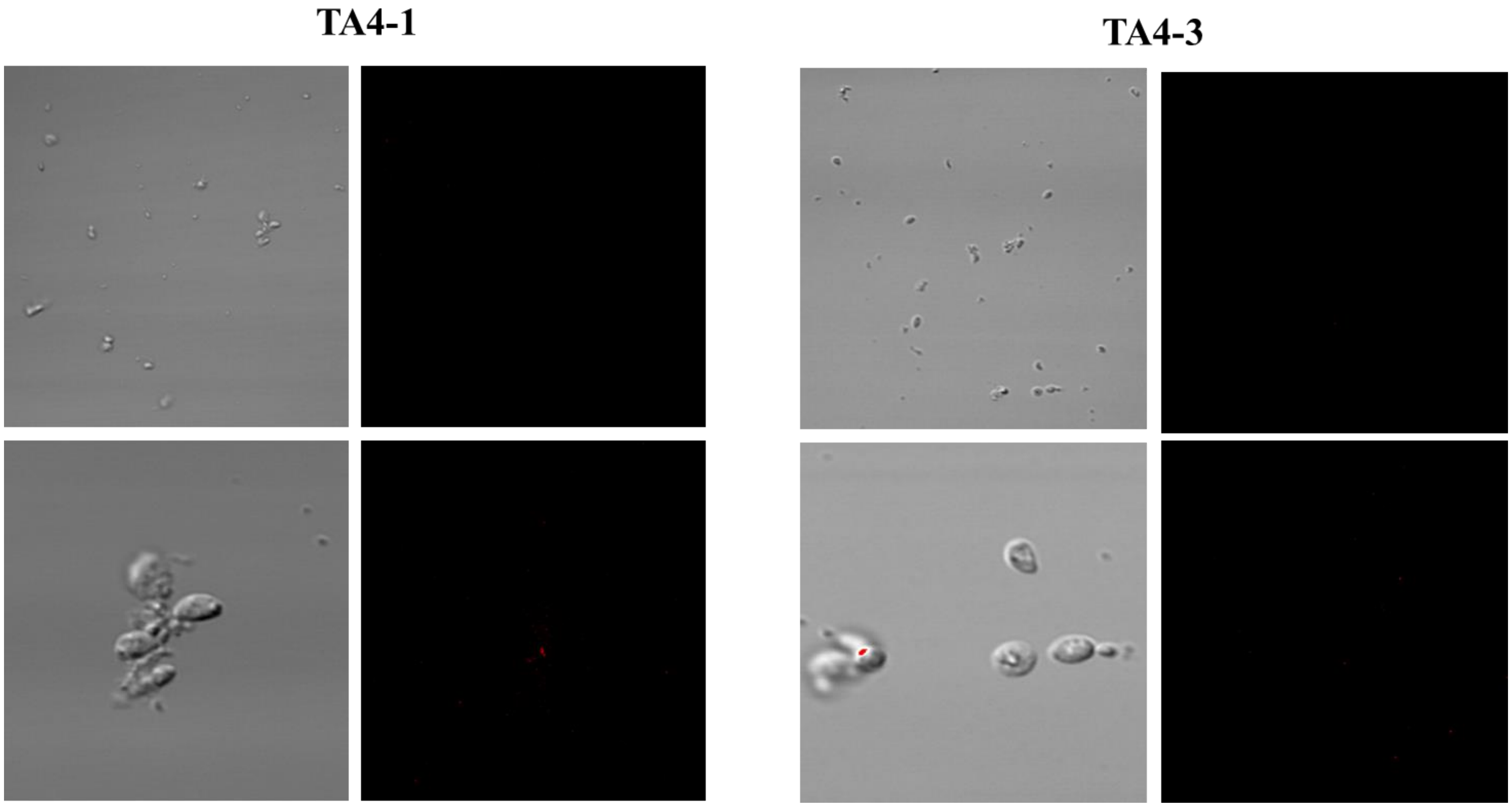
TA4-1
TA4-3

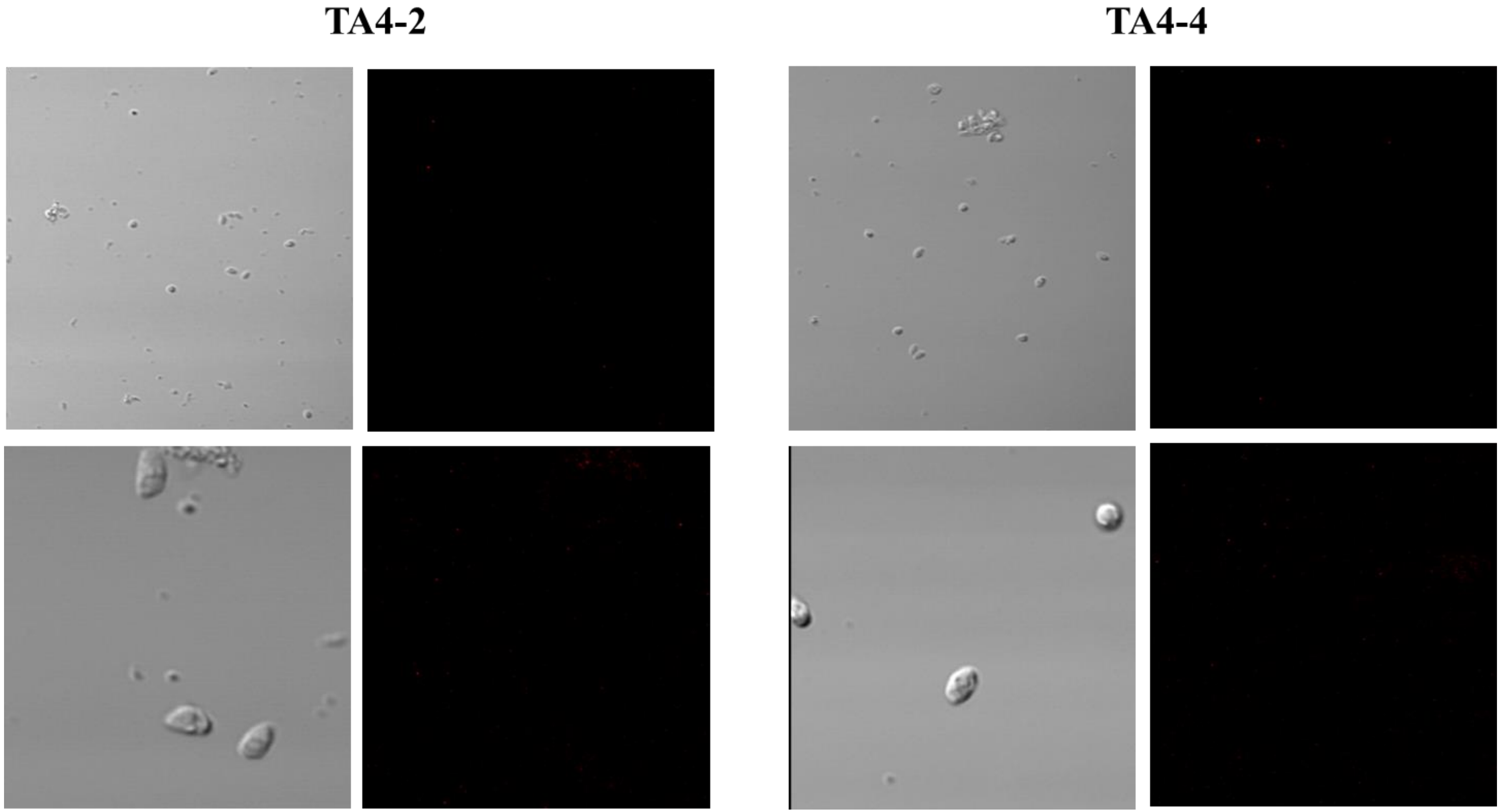
TA4-2
TA4-4

Whole Cyclo S16

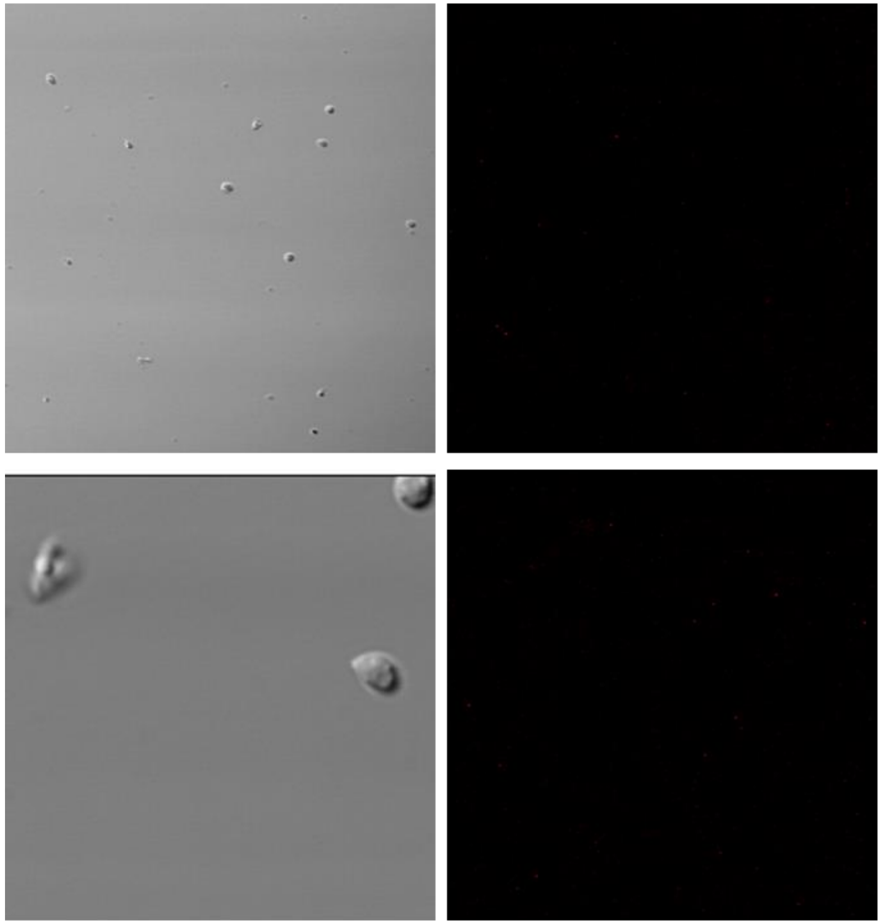

Whole Cyclo S3

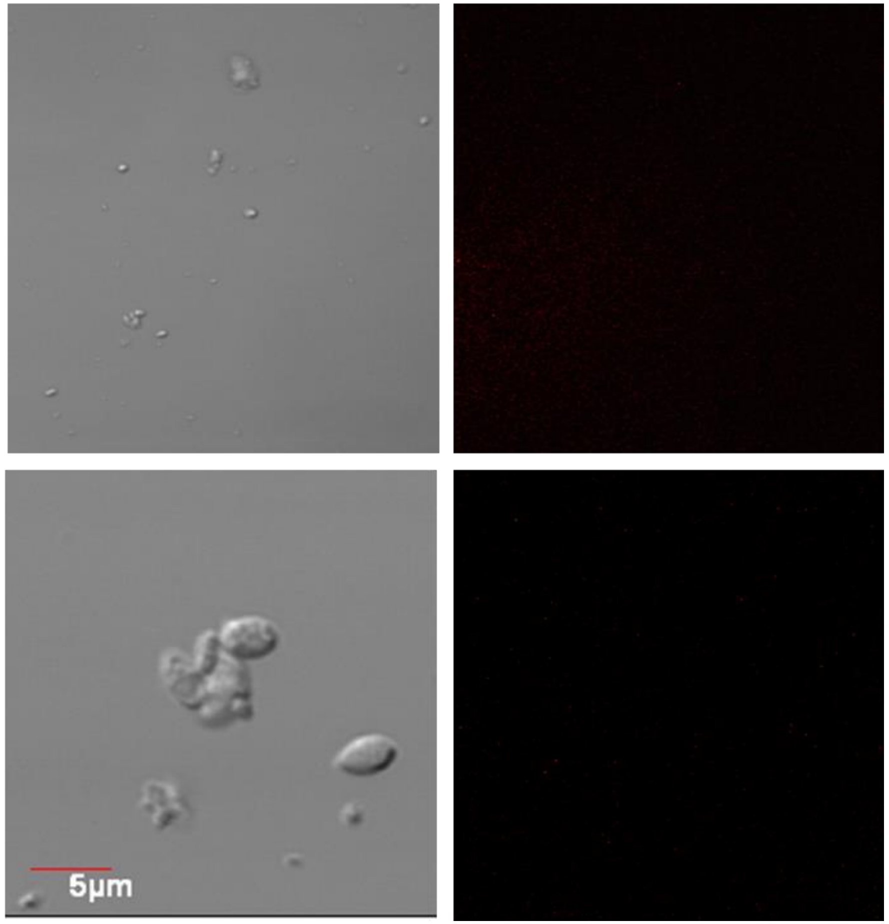

Assay Controls

No Aptamer (-)ve Control | **WP2-1 + Cyclospora (+)ve Control**

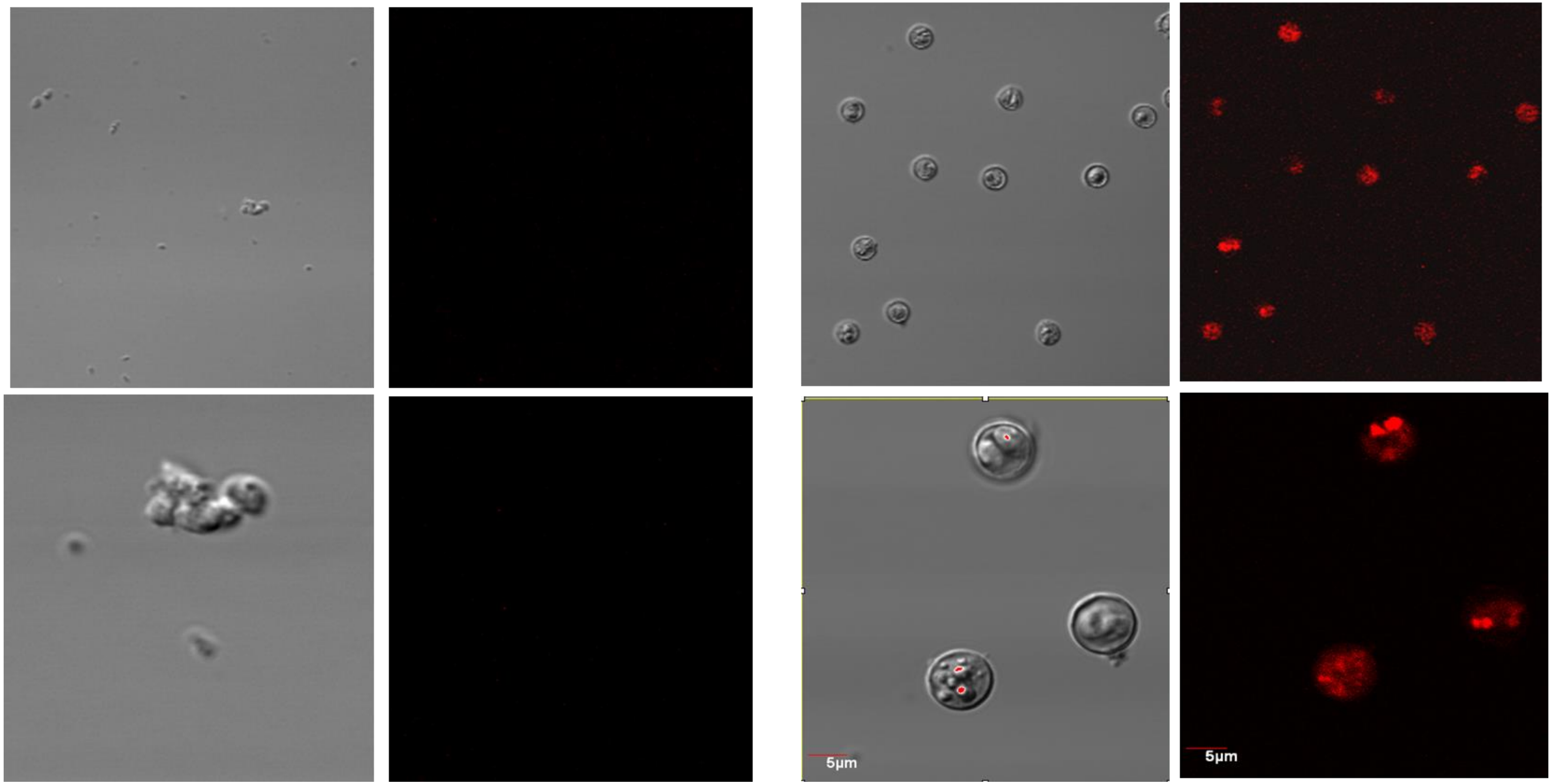

Conclusion: Toxoplasma gondii showed no positive signals with any of the aptamers.
